# A multi-demand operating system underlying diverse cognitive tasks

**DOI:** 10.1038/s41467-024-46511-5

**Published:** 2024-03-11

**Authors:** Weidong Cai, Jalil Taghia, Vinod Menon

**Affiliations:** 1 Department of Psychiatry & Behavioral Sciences, Stanford University School of Medicine, Stanford, CA USA; 2 Wu Tsai Neuroscience Institute, Stanford University, Stanford, CA USA; 3 Department of Information Technology, Uppsala University, Uppsala, Sweden; 4 Department of Neurology & Neurological Sciences, Stanford University School of Medicine, Stanford, CA USA

**Keywords:** Cognitive control, Attention, Dynamical systems

## Abstract

The existence of a multiple-demand cortical system with an adaptive, domain-general, role in cognition has been proposed, but the underlying dynamic mechanisms and their links to cognitive control abilities are poorly understood. Here we use a probabilistic generative Bayesian model of brain circuit dynamics to determine dynamic brain states across multiple cognitive domains, independent datasets, and participant groups, including task fMRI data from Human Connectome Project, Dual Mechanisms of Cognitive Control study and a neurodevelopment study. We discover a shared brain state across seven distinct cognitive tasks and found that the dynamics of this shared brain state predicted cognitive control abilities in each task. Our findings reveal the flexible engagement of dynamic brain processes across multiple cognitive domains and participant groups, and uncover the generative mechanisms underlying the functioning of a domain-general cognitive operating system. Our computational framework opens promising avenues for probing neurocognitive function and dysfunction.

## Abstract (web summary)

A consistent set of brain areas is engaged across diverse cognitive tasks. Here, the authors reveal a unifying latent brain state that predicts performance across seven tasks, linking a core control network to cognitive flexibility and adaptive behaviors.

## Introduction

The human brain is a flexible, yet stable, system that allows rapid and adaptive allocation of cognitive resources to meet moment-by-moment changes in task demands^1–7^. A converging body of evidence now points to a core set of distributed brain areas that are consistently engaged during diverse cognitive tasks^8–15^. This commonality naturally raises the critical and challenging question of how the same brain areas might underlie cognition across multiple task domains^16^. Addressing this question has the potential to uncover mechanisms underlying a multiple-demand, domain-general, functional system underlying cognition and identify transdiagnostic features of cognitive dysfunction in psychiatric and neurological disorders^17^. Here we use a state space hidden Markov model (HMM) to address this challenge. We identify common brain states that are dynamically engaged across seven different cognitive paradigms, across multiple participant cohorts, and demonstrate their behavioral relevance.

Surprisingly, a wide range of cognitive tasks have been found to elicit a common pattern of frontal and parietal network activity^18^, leading to the proposal that a common multiple-demand system may play an adaptive, domain-general, role to achieve task goals^16^. Evidence for the involvement of a network of frontal-parietal cortical regions that are commonly activated across tasks has emerged from a wide range of electrophysiological and fMRI studies^19,20^. However, the shared mechanisms by which such a multiple-demand system contributes to cognition across a broad range of tasks is not known as previous studies have primarily focused on identification of brain areas commonly activated across tasks and have lacked the quantitative rigor needed to link dynamic brain circuits across tasks. Crucially, activation of common brain areas does not necessarily imply engagement of a shared underlying mechanism by which the putative multiple-demand system operates across cognitive domains. An alternative hypothesis is that despite engagement of similar brain regions no common generative mechanisms are engaged across tasks. Identification of a common generative mechanism would provide strong evidence for a domain-general cognitive operating system.

Uncovering general dynamical mechanisms that contribute to multiple cognitive tasks independently in a quantitatively rigorous manner is challenging because of the lack of computational models for characterizing shared processes across tasks that vary along multiple cognitive dimensions and behavioral contingencies, as well as data acquisition protocols and participant cohorts. A critical issue here is that uncovering the operation of such a multiple-demand system requires analysis of latent dynamical processes that underlie different cognitive tasks.

State space models that capture dynamic changes in coactivation patterns across brain regions involved in cognitive control offer a powerful tool in this regard. Latent space models are now being widely explored as promising tools for analyzing rich patterns of electrophysiological activity across diverse neural systems and behaviors, as they do not depend on known relationships between neural activity and external experimental variables^21,22^. Latent state models characterize patterns of covariation across a neuronal population to reveal its internal state^22^. These models have proven useful for visualizing population-level neural activity, relating activity to behavior, and interrogating the dynamic mechanisms that mediate population-level computations^22–26^. Moreover, comparisons of large-scale neural activity across participants and different experimental tasks is challenging without models and rigorous computational methods for linking neural processes across two or more datasets. State space algorithms that probe a shared space where they can be directly compared, have proven to be valuable for comparing high-dimensional neural activities across times, subsets of neurons, and individuals^27^. Our models are tailored to achieve similar goals with human fMRI data.

Analogous to recent neurophysiological investigations, we use a latent space HMM which models covariation in fMRI activity with a Bayesian switching dynamical systems state space (BSDS) algorithm^28^. Specifically, brain states are characterized by a unique pattern of time-varying activation and functional connectivity linking regions of interest^28^. Importantly, these brain states do not occur at random, they are temporally correlated in a Markovian sense—the brain state at a given time depends on the state in the previous time instance. Different from conventional approaches based on external experimental variables, at each time point, a brain state *i* occurs with a probability *p(i)* that fluctuates over time, and a transition probability *A(i,j)* of transitioning from state *i* to state *j*. BSDS implements an unsupervised learning algorithm to determine brain states, their probability of occurrence at each time point, transition probability, and the mean activation and functional connectivity. Moreover, unlike conventional methods, BSDS does not directly compute time-varying activation and functional connectivity using arbitrary moving windows or impose temporal boundary associated with predefined task conditions. Instead, BSDS learns latent representations and states in a unified framework by optimization of single objective function within a Bayesian framework. Our generative model thus allowed us to infer time-varying activation and functional connectivity associated with each brain state with minimal assumptions. Importantly, the generative model implemented by BSDS provides posterior probability distributions of model parameters from one cognitive task which can be used as priors for estimating model parameters from other tasks, thereby facilitating analysis of a domain-general functional multiple-demand systems across cognitive domains and participant cohorts.

**Fig. 1:**
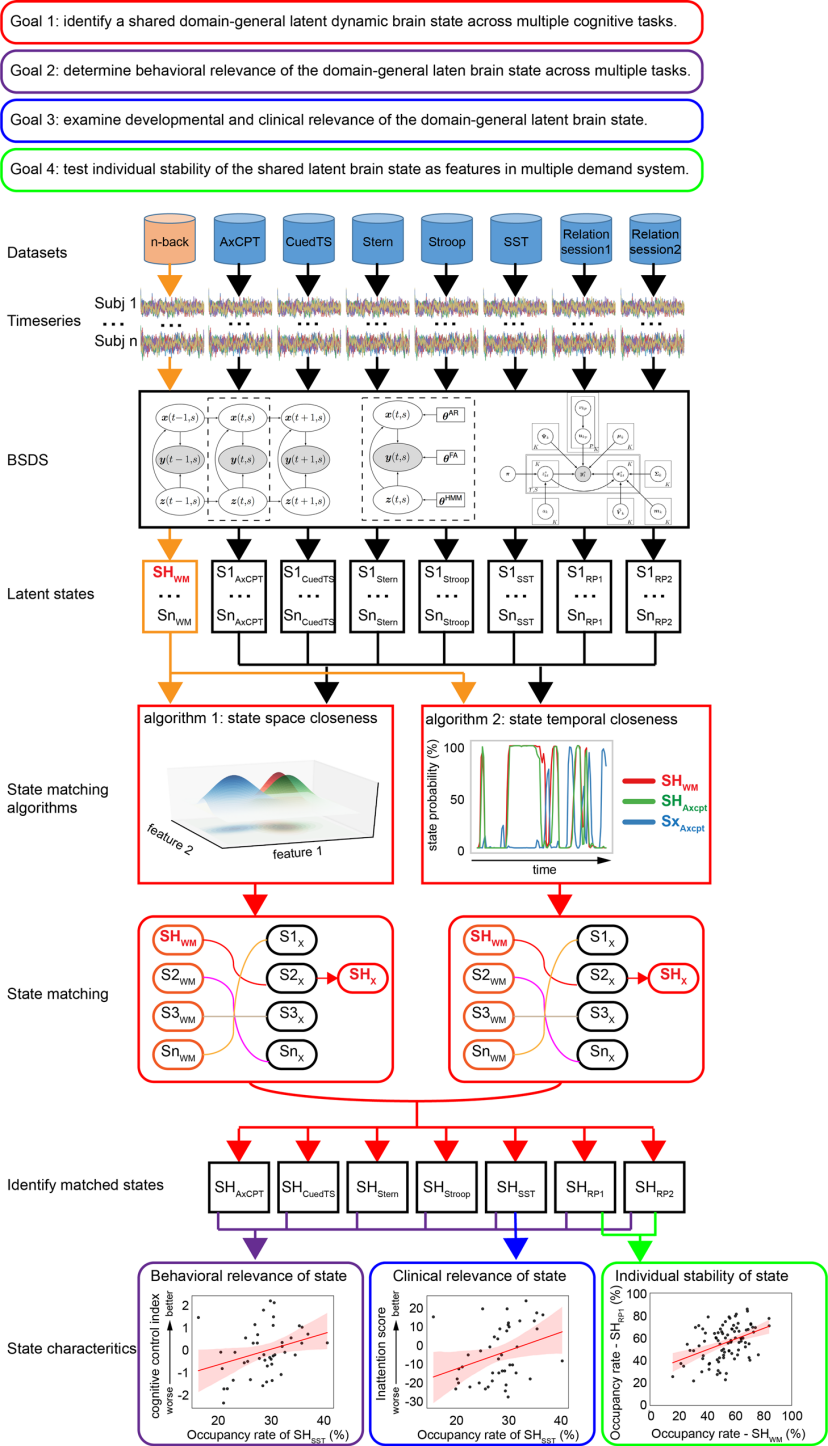
Overview of data analysis strategy and pipeline. We applied a Bayesian switching dynamic systems (BSDS) to time series extracted from brain regions constituting a multiple-demand system involved in cognitive control. We investigated latent brain states across seven different cognitive tasks and four different datasets. First, to demonstrate a shared latent brain state across cognitive domains, we matched task-specific latent brain states with the task-optimal latent brain state SH_WM_ associated with high-load working memory task condition. Two different state matching algorithms demonstrated convergent results. Second, the relationship between the shared latent brain state and cognitive performance in each task was investigated using both multivariate canonical correlation and univariate correlation analysis. Third, we examined the shared latent brain state and its relation to clinical measures of inattention in a developmental cohort. Fourth, we evaluated similarity of latent brain states within individuals across cognitive tasks. WM n-back working memory task, AxCPT Ax continuous performance task, CuedTS Cued task switching task, Stern Sternberg working memory task, Stroop Stroop interference task, SST Stop-signal task, RP Relation processing task. The regression estimate is presented with 95% confidence interval (shaded area).

The overarching aim of our study was to characterize shared latent brain mechanisms associated with a multiple-demand cognitive operating system across a wide range of cognitive tasks, and determine their association with individual differences in cognitive control abilities. To accomplish this, we characterized the Markovian properties of shared brain states by examining the probability of brain state occurrence, their transition probabilities, and associated activation and connectivity features in seven widely used cognitive task paradigms. We identified brain states in each task and examined their correspondence with brain states in a task canonical *n-back* working memory reference task^28^. Using the *n*-back working memory as a reference task, we asked whether task-optimal latent brain states that occur during the high cognitive load condition in the *n*-back task are also engaged during each of the other seven cognitive tasks. Our choice of the working memory task was motivated both by the fact that it is widely used to probe cognitive function and dysfunction^29,30^, and by our identification of optimal and non-optimal brain states associated with cognitive performance and decision-making^28^. Whether a latent brain state in an independent task matches an optimal working memory task brain state was determined by how close they were in their latent space parameters (Fig. 1).

Our first major goal was to characterize brain states in an independent set of four cognitive control tasks acquired as part of the Dual Mechanism of Cognitive Control (DMCC) study^31^. The DMCC study includes the AX continuous performance task (AxCPT), cued task switching task (CuedTS), Sternberg working memory task (Sternberg) and Stroop interference task (Stroop). All four cognitive tasks have been widely used to examine different aspects of cognitive control^32–34^. We hypothesized that our approach would uncover a common brain state across the four cognitive control tasks, thereby elucidating a shared circuit mechanism underlying the multiple-demand system in an adult cohort.

Crucially, we investigated whether brain states identified by our model in each of the four cognitive control tasks are behaviorally relevant. We assessed brain-behavior relations in relation to occurrence rates of brain states engaged during task performance. We then investigated whether the occurrence of the shared brain states across cognitive task domains was preferentially linked to task performance. Extending our previous finding that the inability to engage such a state was associated with poor performance in the working memory task^28^, we hypothesized that occurrence of the shared brain state underlying the multiple-demand system would be behaviorally relevant across the four cognitive control tasks.

Our third major goal was to investigate whether occurrence of the shared brain state is also developmentally relevant, and whether its occurrence could predict attention deficits characteristic of childhood Attention Deficit/Hyperactivity Disorder (ADHD), a neurodevelopmental disorder characterized by prominent deficits in cognitive control^35,36^. We examined whether the same generative model could be used to investigate latent brain states in typically developing children and children with ADHD performing a stop-signal task (SST), a standard paradigm for probing impulsivity and response inhibition^37^. We hypothesized that difficulty in engaging the shared latent brain state would predict cognitive control deficits and severity of inattention symptoms in children.

Finally, to investigate the broader application of our model beyond canonical cognitive control and working memory tasks, we investigated a relational processing (RP) fMRI task from the HCP^38^. Because the RP and *n*-back working memory tasks were performed in the same individuals, this allowed us to determine whether individuals have similar brain state features across tasks. We hypothesized that individuals would show similar latent brain state features across cognitive tasks, reflecting intra-individual stability of features in the multiple-demand cognitive system.

Our study builds on previous research using a putative multiple-demand system identified using a large sample from the Human Connectome Project (HCP)^28^. Replicability is a major challenge in neuroscience and a key goal here is to investigate the same brain regions, encompassing key nodes of the salience, default-mode, and lateral frontoparietal networks, which are consistently activated across various cognitive tasks^30,39,40^. Using reference generative models associated with a canonical working memory task, we then examine whether latent brain states from the reference task also play a role in the underlying cognitive processes of diverse tasks. We uncover a shared optimal dynamic brain state underlying a broad range of cognitive domains, tasks, across multiple participant cohorts and data acquisition protocols. Findings provide critical insights into the dynamical circuit mechanisms by which a common multiple-demand system contributes to a wide range of cognitive functions.

## Results

Our analysis strategy and data analysis pipeline are shown in Fig. 1 and Supplementary Fig. 1 (see also Methods for additional details). We leveraged a total of seven different experiments across a wide range of cognitive domains (Supplementary Table S1). An important feature of all fMRI datasets was that they were acquired at high temporal resolution, with sub-2-second sampling rates ranging from 0.49 to 1.2 s. This allowed us to capture rich temporal dynamics in fMRI data in each task and determine shared underlying latent structures and relate them to cognitive task performance.

In a previous study using an *n*-back working memory task with 122 participants from the HCP, we uncovered an optimal brain state for high load cognitive control (SH_WM_) which occurs preferentially during the 2-back, high-load, working memory task condition, over during the low-load and fixation task conditions^28^. We chose this task because the *n*-back task, and in particular the 2-back condition, requires sustained engagement of attention and cognitive control resources for achieving good task performance. We therefore used *n*-back task states as a template for analysis of shared latent states across tasks. The latent brain state was defined by unique patterns of activity and functional connectivity between key nodes of the salience, central executive, and default mode networks (SN, CEN and DMN, respectively), three large-scale cingulo-opercular and frontoparietal neurocognitive networks whose dynamic interactions play an essential role in cognition^28,41–43^. The ROIs included bilateral anterior insula (AI), middle frontal gyrus (MFG), frontal eye field (FEF), intraparietal sulcus (IPS) and dorsomedial prefrontal cortex (DMPFC), ventromedial prefrontal cortex (VMPFC) and posterior cingulate cortex (PCC).

State SH_WM_ was determined to be an “optimal latent brain state” because (1) it had higher occupancy rate in the 2-back, compared to the 0-back, task condition, where occupancy rate refers to a measure for how often a latent brain state occurs during a period of time, (2) it had higher occupancy rate than other latent brain states in the 2-back task condition, and (3) its occupancy rate predicted behavioral performance^28^. Based on these observations, we used the SH_WM_ as a reference state (Supplementary Fig. S2) and identified brain states in each of the other six cognitive tasks whose spatiotemporal features best matched SH_WM_. In other words, in each of the other six tasks *X*, we identified state SH_X_ which was matched to SH_WM_, the optimal state for the 2-back working memory task. Importantly, we then determined whether the likelihood of engaging such “matched optimal” states predicted cognitive control abilities, including individual differences in task-specific measures of cognitive task performance and clinical symptoms associated with cognitive control deficits.

### DMCC tasks: AxCPT

We conducted a parallel set of analyses on four different cognitive control tasks from the DMCC study, including the AxCPT, CuedTS, Sternberg, and Stroop tasks^31^.

The AxCPT is widely used to investigate cognitive control mechanisms associated with proactive and reactive control^32^. In this task, participants are presented with the letters A or B followed by letters X or Y (or not “X”), comprising AX, AY, BX, and BY pairs. They are asked to respond to the probe (“X”) only if it followed the contextual cue (“A”). Participants were to make another response to other cue–probe sequences (“A” then “Y,” “B” then “X,” or “B” then “Y”), each occurring with much lower probability than the target pair (“AX”). Analysis of behavioral performance revealed that RTs were significantly longer in the AY than AX trials (*t*_49_ = 17.44, *p* < 0.001*, Cohen’s d* = 4.933, *two-tailed paired t-test;* Supplementary Table S2) and in the BX than BY trials (*t*_49_ = 8.02*, Cohen’s d* = 2.268, *p* < 0.001, two-tailed paired *t*-test; Supplementary Table S2). Longer RT in AY and shorter RT in BX are associated with better proactive and reactive control, respectively^44,45^.

**Fig. 2:**
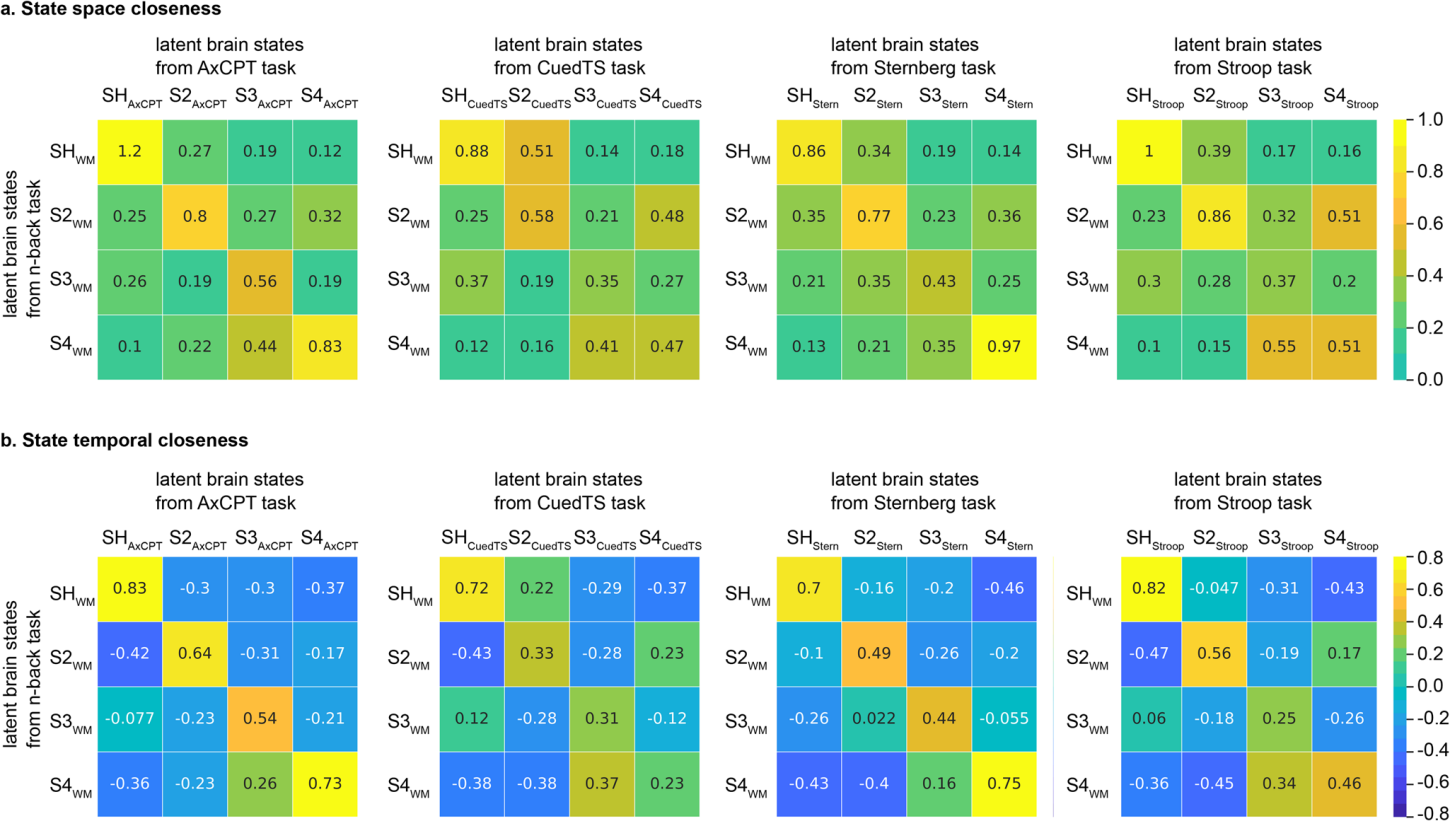
Shared latent brain state across four different dual mode of cognitive control (DMCC) tasks. BSDS uncovered 6 dynamic brain states in the AxCPT (*N* = 50). SH_AxCPT_ showed (**a**) the highest state space closeness (c = 1.2) and (**b**) highest state temporal closeness (*r* = 0.83) with SH_WM_. BSDS uncovered 6 dynamic brain states in the CuedTS task. SH_CuedTS_ showed (**a**) the highest state space closeness (c = 0.88) and (**b**) the highest state temporal closeness (*r* = 0.72) with SH_WM_. BSDS uncovered 4 dynamic brain states in the Sternberg working memory task. SH_Stern_ showed (**a**) the highest state space closeness (c = 0.86) and (**b**) highest state temporal closeness (*r* = 0.7) with SH_WM_. BSDS uncovered 5 dynamic brain state in the Stroop task. SH_Stroop_ showed (**a**) the highest state space closeness (c = 1) and (**b**) the highest state temporal closeness (*r* = 0.82) with SH_WM_. SH_WM_ refers to the high-load dynamic brain state in the n-back working memory task. In each task, the best matched four latent states are illustrated here. Color bars are the scales for state space closeness and state temporal closeness.

BSDS uncovered six latent brain states in the AxCPT task (Supplementary Fig. S3a). Next, we used two different algorithms to determine the correspondence between states in the AxCPT and *n*-back tasks. State temporal closeness measures the similarity of two latent brain states’ temporal profiles (i.e., posterior probability of brain states along time) in the same dataset (see details in methods), whereas state space closeness measures the similarity of two latent brain states’ space feature profiles (i.e., mean and covariance). High temporal correlation and high space closeness indicates better matching of states. Both algorithms identified a single unique state SH_AxCPT_ with the highest state-space closeness (*c* = 1.2, *p* = 0.005) and the highest temporal closeness (*r* = 0.83, *p* = 0.004, *Pearson’s* correlation) with SH_WM_ (Fig. 2). Detailed results from statistical analysis of state matching procedures across tasks are described in Supplementary Information (Supplementary Results and Supplementary Table S13). These results identify SH_AxCPT_ as a brain state matching SH_WM_, the task-optimal reference engaged during working memory.

**Fig. 3:**
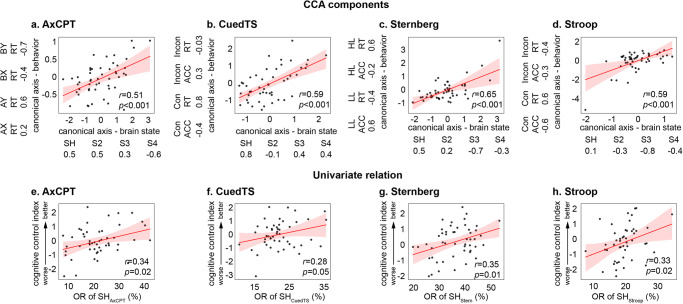
Latent brain states are associated with task performance in all four dual mode of cognitive control (DMCC) tasks. Multivariate CCA revealed significant correlations between occupancy rates of latent brain states and behavioral variables in all the DMCC tasks, including (**a**) AxCPT, (**b**) CuedTS, (**c**) Sternberg and (**d**) Stroop (*N* = 50). In each task, the component in which linear combination of behavioral variables that best represents general cognitive control was selected to investigate the relationship between latent brain state and behavioral performance. Weights of canonical components in each task are presented next to the axes and summarized in Supplementary Table S3. Univariate *Pearson*’s correlation revealed significant correlation between occupancy rate of the multiple-demand brain state (e.g., SH_AxCPT_) and cognitive control index in all the DMCC tasks, including (**e**) AxCPT, (**f**) CuedTS, (**g**) Sternberg task, and (**h**) Stroop. SH_AxCPT_, SH_CuedTS_, SH_Stern_ and SH_Stroop_ refers to the dynamic brain state that matches to SH_WM_ in the AxCPT, CuedTS, Sternberg and Stroop tasks, respectively. AxCPT Ax Continuous performance task, CuedTS Cued task switching task, ACC Accuracy, RT Reaction time, LL Low load, HL High load, Con Congruent; Incon Incongruent, OR Occupancy rate. The regression estimate is presented with 95% confidence interval (shaded area). Source data are provided as a Source data file. P values were not adjusted for multiple comparisons.

*CCA* We used CCA to investigate multivariate relations between brain states and behavioral measures on the AxCPT. We found a significant canonical correlation between occupancy rates of brain states and behavioral performance (*r* = 0.51, *p* < 0.001, *Pearson’s* correlation, Fig. 3a). The occupancy rate of SH_AxCPT_ had the highest positive weight in the brain component and, correspondingly, AY RT had the highest positive weights and BX RT had the most negative weight in the behavioral component, suggesting that increased occupancy rate of SH_AxCPT_ is associated with better task performance (Fig. 3a, Supplementary Table S3). Predictive modeling using CCA showed that predicted canonical brain state measures and predicted canonical behavioral measures were significantly correlated (*r* = 0.31, *p* = 0.02, *Pearson’s* correlation).

*Univariate analysis* We then examined the relation between SH_AxCPT_ and a single index of cognitive control in the AxCPT task. We used a normalized measure of the difference between RTs to “AY” and “BX” trials, a composite measure of proactive and reactive cognitive control processes associated with the AxCPT task^44,45^. We found that the occupancy rate of SH_AxCPT_ was significantly correlated with proactive cognitive control (*r* = 0.34, *p* = 0.02, *Pearson’s* correlation, Fig. 3e). No other state had statistically significant and positive contribution to cognitive control (Supplementary Table S10). This result demonstrates that greater engagement of SH_AxCPT_ is associated with better cognitive control.

### DMCC tasks: CuedTS

The CuedTS paradigm is widely used to investigate cognitive control in response to changing task rules^46,47^. Participants performed a letter-digit task in which they were cued to respond to either the letter or number in target stimuli which consisted of a letter-digit pair (e.g., “D 3”, or “1 A”). Based on the cue, participants had to either categorize the letter as a vowel or consonant, or categorize the digit as even or odd depending on the cue. In incongruent trials, the two stimuli activated competing cue-dependent responses (e.g., “A 3”) whereas in congruent trials, the response was cue-independent (e.g., “A 2”). Analysis of behavioral data revealed that RT on congruent trials was significantly shorter than on incongruent trials (*t*_*49*_ = 3.2, *p* = 0.002, *Cohen’s d* = 0.533, two-tailed paired *t*-test; Supplementary Table S2). Shorter RTs in incongruent trials and lower RT differences between congruent and incongruent trials are associated with better cognitive control ability in this task^46,48^.

BSDS uncovered six latent brain states in the CuedTS (Supplementary Fig. S3b). Using the two different state matching algorithms, we again identified a single unique SH_CuedTS_ with the highest state space closeness (*c* = 0.88, *p* = 0.03) and the highest state temporal closeness (*r* = 0.72, *p* = 0.02, *Pearson’s* correlation) with SH_WM_ (Fig. 2). Detailed results from statistical analysis of state matching procedures across tasks are described in Supplementary Information (Supplementary Results and Supplementary Table S13). These results identify SH_CuedTS_ as a brain state matching SH_WM_, the task-optimal state engaged during working memory.

*CCA* We first used CCA to investigate multivariate relations between brain state and behavioral measures in the CuedTS task. We found a significant canonical correlation between occupancy rate of brain states and behavioral performance (*r* = 0.59, *p* < 0.001, *Pearson’s* correlation, Fig. 3b). The occupancy rate of SH_CuedTS_ had the highest positive weight in the brain component and, correspondingly, incongruent RT had negative weights, suggesting that the increased occupancy rate of SH_CuedTS_ is associated with better task performance (Fig. 3b, Supplementary Table S3). Predictive CCA showed that the predicted canonical brain state measures and predicted canonical behavioral measures were significantly correlated (*r* = 0.35, *p* = 0.01, *Pearson’s* correlation).

*Univariate analysis* We then examined the relation between SH_CuedTS_ and a single index of cognitive control. We used RT differences between congruent and incongruent trials to measure the task-rule congruency effect^46,48^, in which larger values indicate higher levels of cognitive control. We found that the occupancy rate of SH_CuedTS_ was marginally significantly correlated with cognitive control (*r* = 0.28, *p* = 0.05, *Pearson’s* correlation, Fig. 3f). No other state had a statistically significant and positive contribution to cognitive control (Supplementary Table S10). This result demonstrates that greater engagement of SH_CuedTS_ is associated with better conflict resolution.

### DMCC tasks: Sternberg

The Sternberg task is widely used for probing maintenance and manipulation of information in working memory^49–51^. In this task, participants determine whether a probe matches a list of stimuli presented previously. High and low load conditions differ in the number of stimuli that need to be maintained in working memory. Participants showed significantly lower cognitive control in the high, compared to the low, load condition (*t*_*49*_ = 2.69, *p* = 0.01, *Cohen’s d* = 0.761, two-tailed paired *t*-test; Supplementary Table S2). Higher accuracy and shorter RT on high load trials are associated with better working memory capacity^49–51^.

BSDS uncovered four latent brain states in the Sternberg task (Supplementary Fig. S3c). Using two different state matching algorithms, we identified a single unique SH_Stern_ with the highest state space closeness (*c* = 0.86, *p* = 0.04) and the highest state temporal closeness (*r* = 0.7, *p* = 0.03, *Pearson’s* correlation) with SH_WM_ (Fig. 2). Detailed results from statistical analysis of state matching procedures across tasks are described in Supplementary Information (Supplementary Results and Supplementary Table S13). These results identify SH_Stern_ as a brain state matching SH_WM_, the task-optimal state engaged during working memory.

*CCA* We first used CCA to investigate multivariate relations between brain state and behavioral measures in the Sternberg task. We found a significant canonical correlation between occupancy rates of brain states and behavioral performance measures (*r* = 0.65, *p* < 0.001, *Pearson’s* correlation, Fig. 3c). The occupancy rate of SH_Stern_ had the high positive weights in the brain component, suggesting increased occupancy rate of SH_Stern_ is associated with better performance (Fig. 3c, Supplementary Table S3). Predictive CCA showed that the predicted canonical brain state measures and predicted canonical behavioral measures were significantly correlated (*r* = 0.41, *p* = 0.004, *Pearson’s* correlation).

*Univariate analysis* We then examined the relation between SH_Stern_ and a single index of cognitive control in the Sternberg task. We used an efficiency score (Accuracy/RT) to assess working memory function and create a standardized index of cognitive control^49–51^. We found that the occupancy rate of SH_Stern_ was correlated with cognitive control (*r* = 0.35, *p* = 0.01, *Pearson’s* correlation, Fig. 3g). No other state had a statistically significant and positive contribution to cognitive control (Supplementary Table S10). This result demonstrates that greater engagement of SH_Stern_ is associated with better cognitive control in maintaining task-relevant information in working memory.

### DMCC tasks: Stroop

The Stroop is a classic paradigm used to investigate inhibitory control and response conflict^34^. This task requires participants to speak out the font color in which a word is presented. The task involves congruent and incongruent trials, in which the word and the font color of the word are either the same or different, respectively. Analysis of behavioral data revealed that RTs were significantly lower in the incongruent, compared to congruent, trials reflecting greater cognitive control demands in the incongruent condition (*t*_*49*_ = 13.76, *p* < 0.001, *Cohen’s d* = 3.892, two-tailed paired *t*-test; Supplementary Table S2). Shorter RTs in incongruent trials and lower RT differences between congruent and incongruent trials are associated with better cognitive control in this task.

BSDS uncovered five latent brain states in the Stroop task (Supplementary Fig. S3d). Using two different state matching algorithms, we identified a single unique SH_Stroop_ with the highest state space closeness (*c* = 1, *p* = 0.005) and the highest state temporal closeness (*r* = 0.82, *p* = 0.008, *Pearson’s* correlation) with SH_WM_ (Fig. 2). Detailed results from statistical analysis of state matching procedures across tasks are described in Supplementary Information (Supplementary Results and Supplementary Table S13). These results identify SH_Stroop_ as a brain state matching SH_WM_, the task-optimal state engaged during working memory.

*CCA* We first used CCA to investigate multivariate relations between brain state and behavioral measures in the Stroop task. We found a significant canonical correlation between occupancy of brain states and behavioral performance measures (*r* = 0.59, *p* < 0.001, *Pearson’s* correlation, Fig. 3d). Importantly, occupancy rate of SH_Stroop_ had high positive weight in the brain component and, correspondingly, Incongruent RT had negative weight, suggesting increased occupancy rate of SH_Stroop_ associated with faster response in incongruent trials (Fig. 3d, Supplementary Table S3). Predictive CCA showed that the predicted canonical brain state measures and predicted canonical behavioral measures were significantly correlated (*r* = 0.41, *p* = 0.004, *Pearson’s* correlation).

*Univariate analysis* We then examined the relation between SH_Stroop_ and a single index of cognitive control in the Stroop task. Cognitive control was indexed using the difference in RT between congruent and incongruent trials as it is widely used to quantify individuals’ ability to resolve conflict and identify its neural basis^34,52,53^. We found that the occupancy rate of SH_Stroop_ was significantly correlated with cognitive control (*r* = 0.33, *p* = 0.02, *Pearson’s* correlation, Fig. 3h). No other state had a statistically significant and positive contribution to cognitive control (Supplementary Table S10). This result demonstrates greater engagement of SH_Stroop_ is associated with better cognitive control during the Stroop.

Together, these results demonstrate that latent brain states that are well matched to a shared SH_WM_ state in the 2-back working memory task, can be identified in four different cognitive control tasks in the DMCC and that their temporal properties are related to cognitive control in each task (summarized in Supplementary Table S8).

### Stop-signal task (SST)

We used Stop-signal task (SST) data from a cohort of 45 typically developing children and children with ADHD (9–12 years old) acquired at Stanford University. The SST is widely used to probe inhibitory control and its underlying neural mechanism^8,54–56^. Participants were asked to make motor responses to frequent Go signals. Occasionally, a Go signal was followed by a Stop signal, which required participants to withhold the prepotent Go response. Participants achieved a high accuracy on Go trials (94 ± 5%) and near 50% accuracy on Stop trials (51 ± 6%) (Supplementary Table S4). Importantly, the stop-signal reaction time (SSRT) estimated from the Race model quantifies one’s stopping speed^37,57^. Shorter SSRTs are associated with better inhibitory control.

**Fig. 4:**
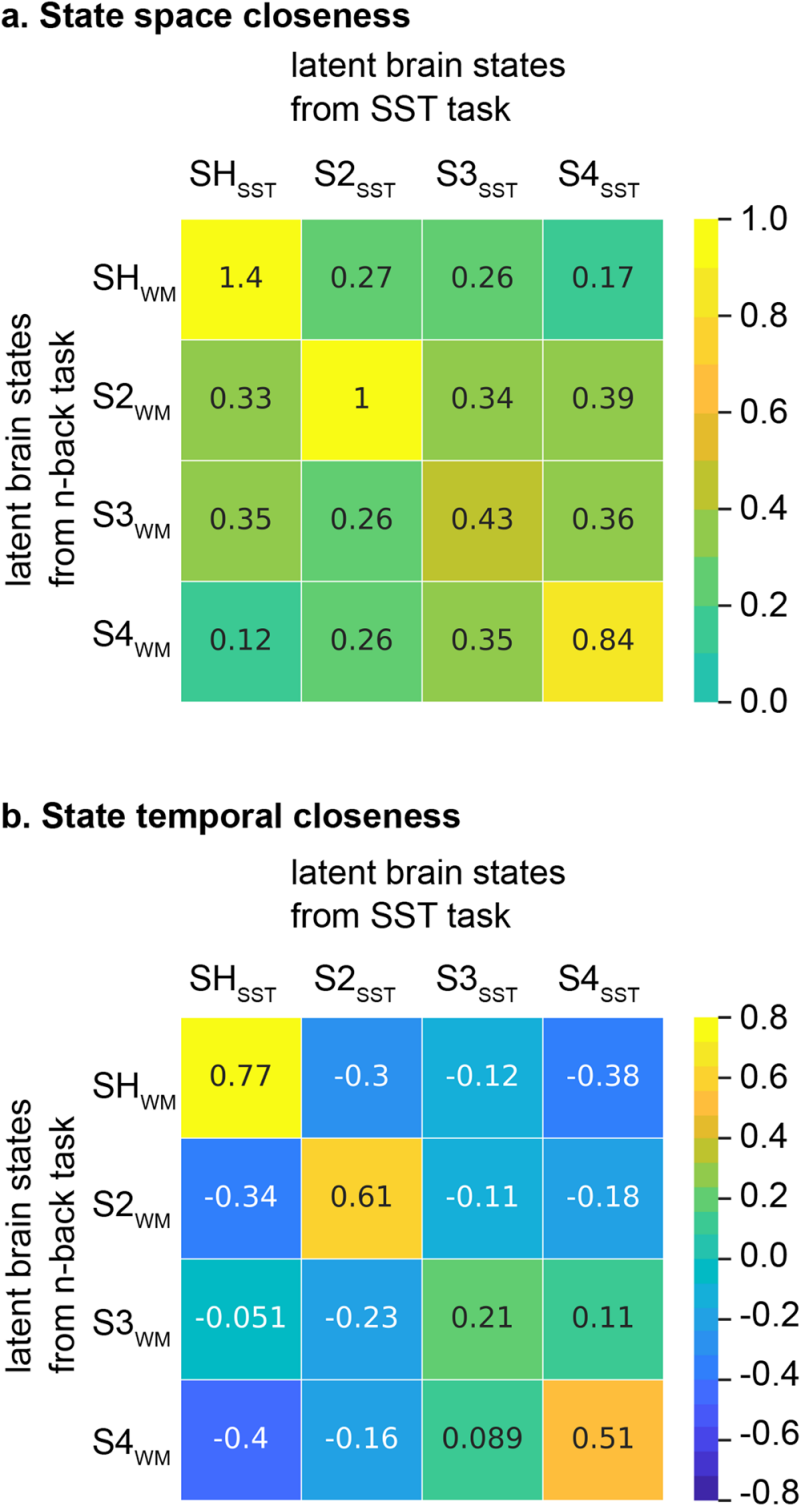
Shared latent brain states in the stop signal task (SST). **a** BSDS uncovered 4 dynamic brain states (*N* = 45). SH_SST_ showed the highest state space closeness (*r* = 1.4). **b** SH_SST_ also showed the highest state temporal closeness (c = 0.77) with SH_WM_. SH_WM_ refers to the high-load dynamic brain state in the n-back working memory task. SH_SST_ refers to the dynamic brain state that matches to SH_WM_ in the SST. Color bars are the scales for state space closeness and state temporal closeness.

BSDS uncovered four latent brain states in the SST (Supplementary Fig. S4). Using two different state matching algorithms, we again identified a single unique SH_SST_ with the highest state space closeness (*c* = 1.4, *p* = 0.005) and the highest state temporal closeness (*r* = 0.77, *p* = 0.008, *Pearson’s* correlation) with the SH_WM_ (Fig. 4). Detailed results from statistical analysis of state matching procedures across tasks are described in Supplementary Information (Supplementary Results and Supplementary Table S13). These results identify SH_SST_ as a brain state matching SH_WM_, the task-optimal state engaged during working memory.

**Fig. 5:**
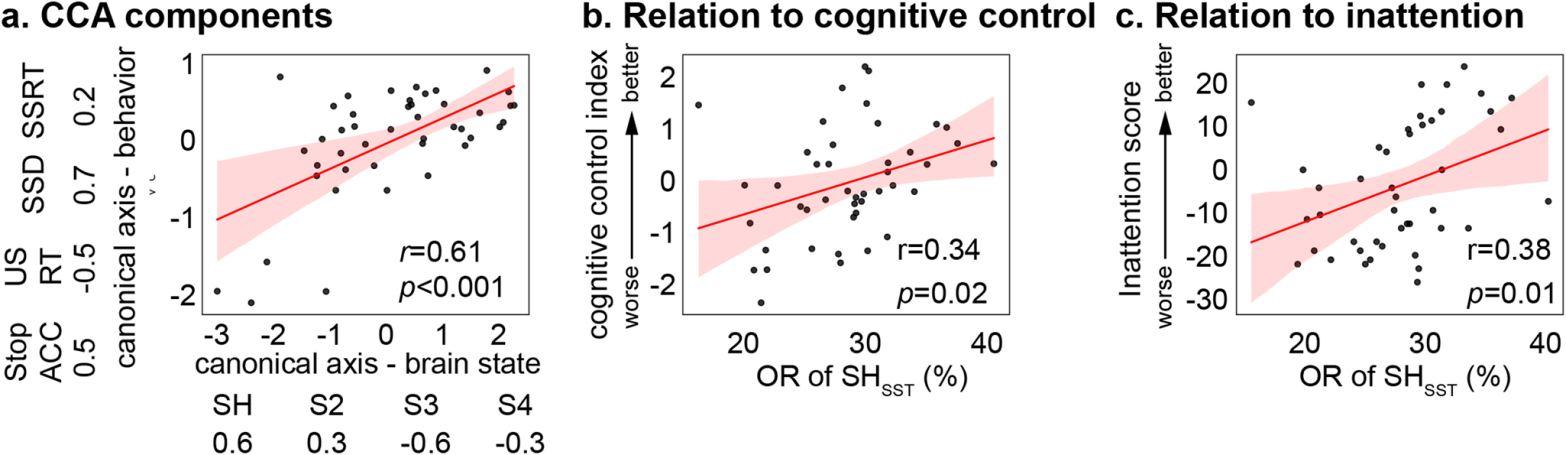
Latent brain states are associated with task performance and inattention symptoms in the stop signal task (SST). **a** Multivariate CCA revealed significant canonical correlations between occupancy rates of latent brain states and behavioral variables in SST (*N* = 45). The component in which linear combination of behavioral variables that best represents general cognitive control was selected to investigate the relationship between latent brain state and behavioral performance. Weights of canonical components in each task was summarized in Supplementary Table S5. **b** Univariate *Pearson*’s correlation analysis revealed that OR of SH_SST_ is significantly correlated with cognitive control index in the SST. **c** OR of SH_SST_ is significantly correlated with inattention scores from the SWAN. SH_SST_ refers to the dynamic brain state that matches to SH_WM_ in the SST. ACC Accuracy, RT Reaction Time, US Unsuccessful Stopping, SSD Stop Signal Delay, SSRT Stop Signal Reaction Time, SWAN Strengths and Weaknesses of ADHD-symptoms and Normal-behavior rating scale, OR Occupancy rate. The regression estimate is presented with 95% confidence interval (shaded area). Source data are provided as a Source data file.

*CCA* We first used CCA to investigate multivariate relations between brain state and behavioral measures in the SST. We found a significant canonical correlation between occupancy rates of brain states and behavioral performance measures (*r* = 0.61, *p* < 0.001, *Pearson’s* correlation, Fig. 5a). The occupancy rate of SH_SST_ had high positive weight in the brain component and, correspondingly, Stop Accuracy had high positive weight, suggesting increased occupancy rate of SH_SST_ is associated with better performance (Fig. 5a, Supplementary Table S5). Predictive CCA showed that the predicted canonical brain state measures and predicted canonical behavioral measures were significantly correlated (*r* = 0.43, *p* = 0.004, *Pearson’s* correlation).

*Univariate analysis* We then examined the relation between SH_SST_ and a single index of cognitive control in the SST. Cognitive control on the SST was indexed using the SSRT^37,57^. To facilitate comparison with all other tasks in this study, we used 1/SSRT so that larger values indicate better cognitive control. The occupancy rate of SH_SST_ was significantly correlated with this index of cognitive control (*r* = 0.34, *p* = 0.02, *Pearson’s* correlation) (Fig. 5b). No other state had a statistically significant and positive contribution to cognitive control (Supplementary Table S10). This result demonstrates that greater engagement of SH_SST_ is associated with faster stopping.

To investigate links with clinically relevant measures associated with ADHD, we investigated the relationship between the posterior probability of the matched state SH_SST_ and severity of inattention symptoms. We found that the posterior probability of SH_SST_ was significantly correlated with the inattention score (*r* = 0.38, *p* = 0.01, *Pearson’s* correlation, Fig. 5c). No other state had a statistically significant and positive contribution to the inattention score (Supplementary Table S11). This result demonstrates that greater engagement of SH_SST_ is associated with less severe inattention symptoms.

### Leave one ROI out analysis reveals key role of MFG across cognitive tasks

To quantify the impact of each brain region on the similarity between SH_WM_ and SH_X_, where *X* represents all other tasks, we conducted a leave one ROI out analysis. Specifically, we computed the change (delta) in the Kullback–Leibler divergence across model parameters between states SH_WM_ and SH_X_ before and after removing each brain region’s latent state features. 1/delta was used to quantify the effect of the virtual lesion on state similarity. We found that the MFG, encompassing the dorsolateral prefrontal cortex, has the highest impact on state similarity across the AxCPT, CuedTS, Sternberg, Stroop, and SST tasks (Supplementary Fig. S5). This result suggests that the MFG is a critical brain region underlying the shared high-load brain state across multiple cognitive tasks.

### Relational processing (RP) task from the human connectome project

Finally, we investigated the relational processing (RP) task to further characterize generalizability beyond the standard cognitive control and working memory tasks investigated above. BSDS was used to identify latent brain states in two different sessions (Sessions 1 and 2), each lasting 3 min, and to determine replicability of our findings. In the relational task condition, participants were asked to judge whether two pairs of objects differed along the same dimension. In the control condition, participants were asked to decide whether an object matched top objects on a pre-specified dimension. We used an efficiency score (Accuracy/RT) to quantify performance and standardize it to generate a composite measure of cognitive control (see Methods for details). In both sessions 1 and 2, participants showed lower performance during relational processing than the matching control task (session 1: *t*_*89*_ = 16.71, *p* < 0.001, *Cohen’s d* = 3.523, two-tailed paired *t*-test; session 2: *t*_*89*_ = 14.8, *p* < 0.001, *Cohen’s d* = 3.121, two-tailed paired *t*-test; Supplementary Table S6), suggesting that the relation processing task is more cognitively demanding.

**Fig. 6:**
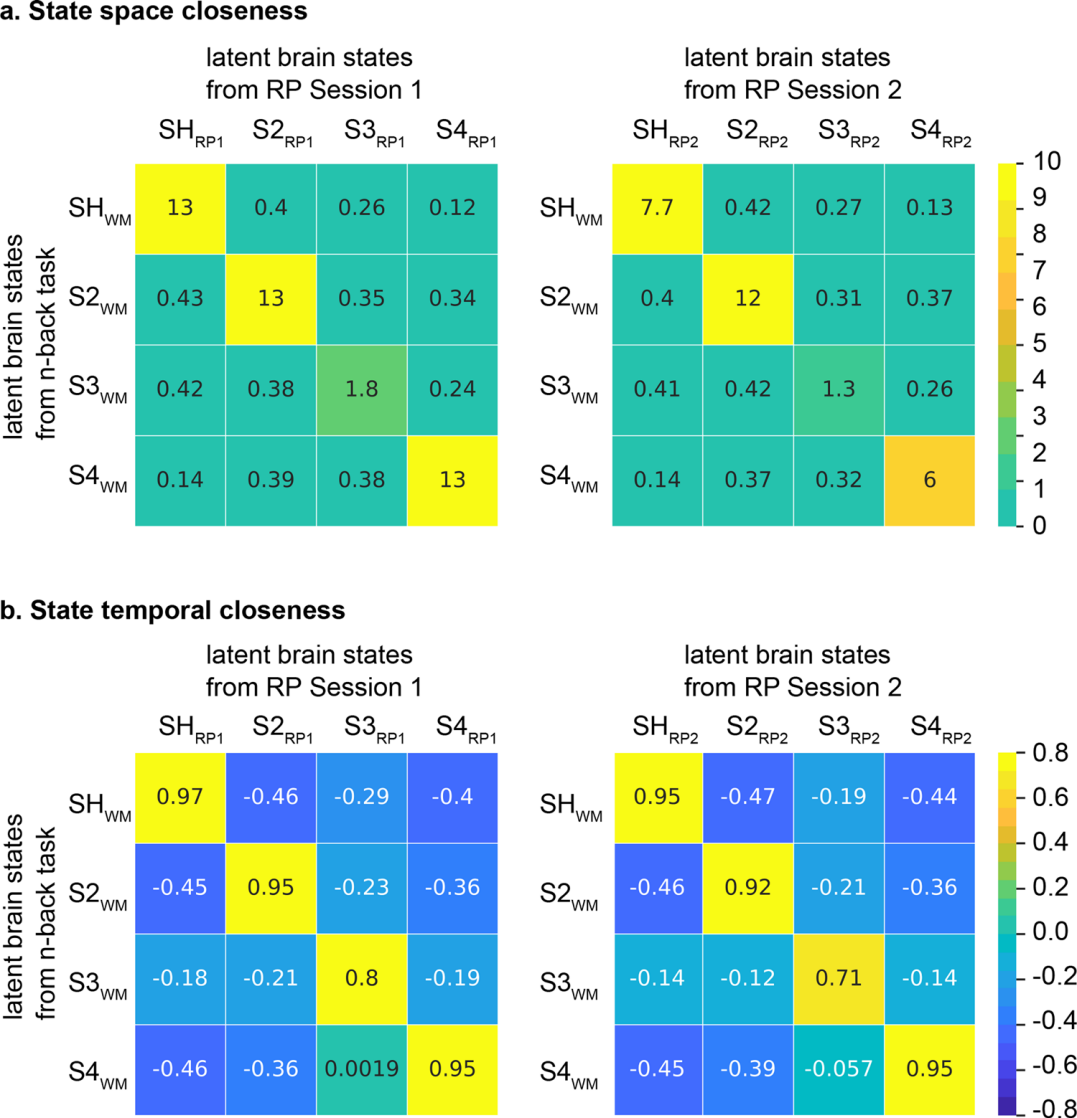
Shared latent brain states in the Relational Processing (RP) task. BSDS uncovered 4 dynamic brain states in both Sessions 1 and 2 (*N* = 90). SH_RP1_ has (**a**) the highest state space closeness (c = 13) and (**b**) the highest state temporal closeness (*r* = 0.97) with SH_WM_ in Session 1. SH_RP2_ has (**a**) the highest state space closeness (c = 7.7) and (**b**) the highest state temporal closeness (*r* = 0.95) with SH_WM_ in Session 2. SH_WM_ refers to the high-load dynamic brain state in the n-back task. SH_RP1_ and SH_RP2_ refers to the dynamic brain states that matches to SH_WM_ in the Relational task sessions 1 and 2, respectively. Color bars are the scales for state space closeness and state temporal closeness.

In Session 1 of the RP task, BSDS uncovered four latent brain states (Supplementary Fig. S6). State matching analysis identified a single unique SH_RP1_ with the highest state space closeness (*c* = 13, *p* = 0.005) and the highest state temporal closeness (*r* = 0.97, *p* = 0.004, *Pearson’s* correlation) with SH_WM_ in Session 1 (Fig. 6). These findings were replicated in Session 2 data. Again, BSDS revealed four latent brain states, with a single unique matched state SH_RP2_ that had the highest state space closeness (*c* = 7.73, *p* = 0.005) and the highest state temporal closeness (*r* = 0.95, *p* = 0.004, *Pearson’s* correlation) with SH_WM_ (Fig. 6). Detailed results from statistical analysis of state matching procedures across tasks are described in Supplementary Information (Supplementary Results and Supplementary Table S13). These results identify SH_RP1_ and SH_RP2_ as brain states matching SH_WM_, the task-optimal state engaged during working memory.

**Fig. 7:**
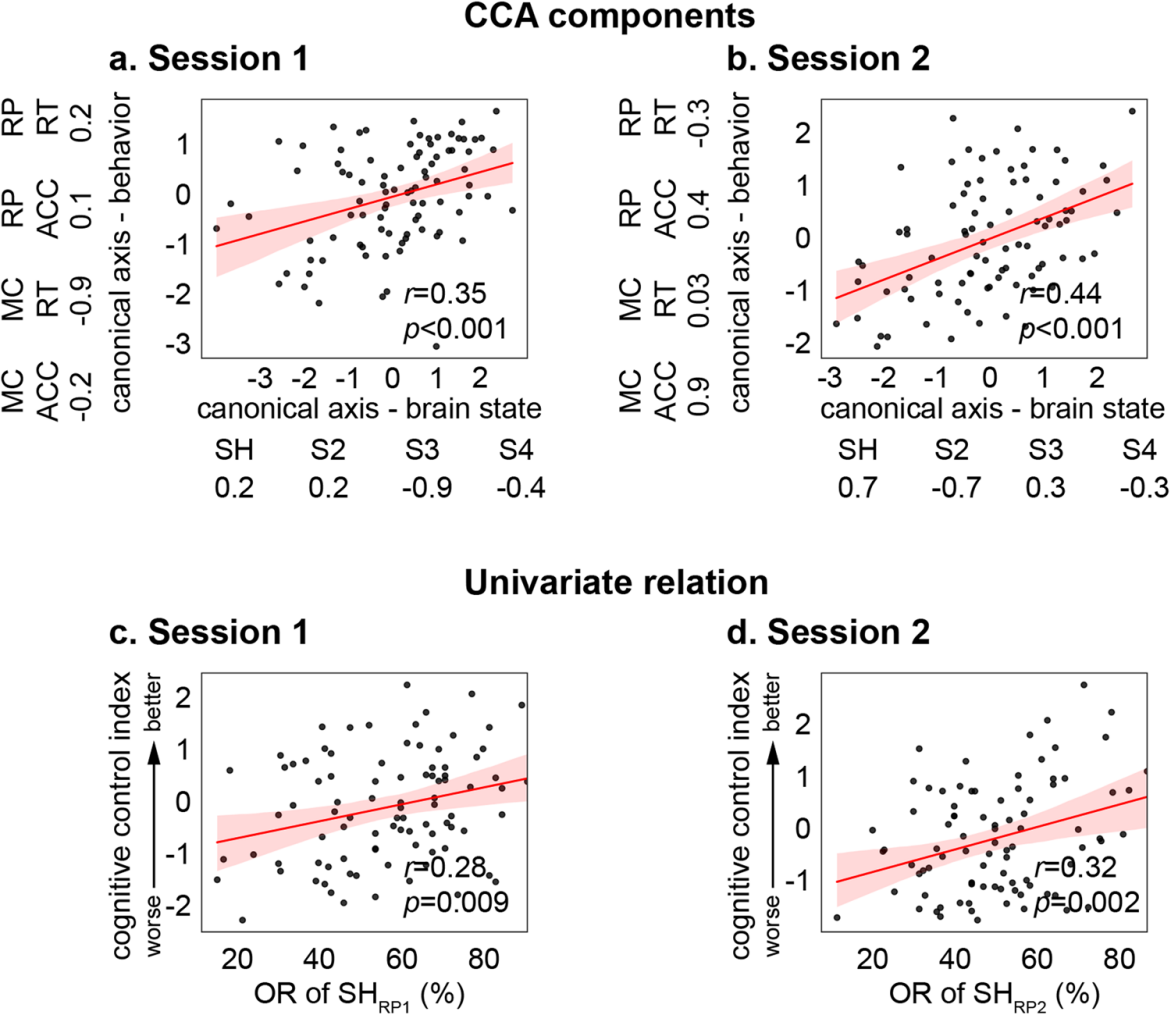
Latent brain states are associated with task performance in the Relational Processing (RP) task. Multivariate CCA revealed significant correlations between occupancy rates of latent brain states and behavioral variables in the HCP RP task session 1 (**a**) and 2 (**b**) (*N* = 90). In each task, the component in which linear combinations of behavioral variables that best represents general cognitive control was selected to investigate the relationship between latent brain state and behavioral performance. Weights of canonical components in each task are summarized in Supplementary Table S7. Univariate *Pearson*’s correlation analysis revealed that OR of SH_SST_ is significantly correlated with cognitive control index in the HCP RP session 1 (**c**) and 2 (**d**). SH_RP1_ and SH_RP2_ refers to the dynamic brain states that matches to SH_WM_ in the Relational task session 1 and 2, respectively. HCP Human Connectome Project, RP Relational Processing, MC Matching Control, ACC Accuracy, RT Reaction Time, OR Occupancy rate. The regression estimate is presented with 95% confidence interval (shaded area). Source data are provided as a Source data file. *P* values were not adjusted for multiple comparisons.

*CCA* We first examined multivariate patterns of the canonical component that best represents general cognitive control and the pattern of the canonical component in the latent brain state. We found a significant canonical correlation between latent brain states and behavioral performance (session 1: *r* = 0.35, *p* < 0.001, *Pearson’s* correlation, Fig. 7a, session 1: *r* = 0.44, *p* < 0.001, *Pearson’s* correlation, Fig. 7b). Importantly, the occupancy rate of SH_RP_ had high positive weights in the brain component in both sessions and, correspondingly, accuracy had positive weights, suggesting increased occupancy rate of SH_RP_ is associated with better task performance (Fig. 7, Supplementary Table S7). Predictive CCA showed that the predicted canonical brain state measures and predicted canonical behavioral measures in Session 2, but not Session 1, were significantly correlated (*r* = 0.31, *p* = 0.004, *Pearson’s* correlation).

*Univariate analysis* We then examined the relation between SH_RP_ and a single index of cognitive control in the relational processing task. Cognitive control on the task was indexed using task efficiency (Accuracy/RT). In Session 1, we found that the occupancy rate of SH_RP1_ was significantly correlated with cognitive control (*r* = 0.28, *p* = 0.009, *Pearson’s* correlation). This finding was replicated in Session 2 (*r* = 0.32, *p* = 0.002, *Pearson’s* correlation). No other state had a statistically significant and positive contribution to cognitive control (Supplementary Table S10). These results demonstrate that greater engagement of SH_RP_ is associated with better cognitive performance in the relational processing task, and furthermore, that this relation is reproducible.

### Shared brain state for cognition within individuals

Taking advantage of the HCP within-subjects design in which, the relational processing (RP) and the *n*-back working memory tasks were both performed by the same individuals, we then determined whether the temporal properties of the shared optimal brain state was reliable across participants. Specifically, we examined the relation between the occupancy rates associated with latent states SH_RP_ and SH_WM_. We found a significant correlation in both Session 1 (*r* = 0.43, *p* < 0.001, *Pearson’s* correlation; Supplementary Fig. S7a) and Session 2 (*r* = 0.24, *p* = 0.02, *Pearson’s* correlation; Supplementary Fig. S7b).

These results provide evidence for similarities in intra-individual latent state across distinct cognitive tasks.

### Model-based brain states vs. conventional activation and connectivity analyses

We conducted additional analyses examining the similarity of activation/deactivation and connectivity profiles determined by our BSDS brain-state model, and contrasted this with similarity measures from conventional general linear regression analyses (Supplementary Methods and Supplementary Fig. S8). In each of the seven cognitive tasks, we found that our BSDS model performed better at capturing common brain states across tasks than conventional general linear model-based activation and connectivity profiles (Supplementary Fig. S9).

### Robustness of state matching with respect to sample size in the *n*-back task

To examine the robustness of our findings with respect to the sample size used in generating the reference optimal latent brain state SH_WM_, we leveraged a larger sample (*N* = 415 participants) from the HCP *n*-back working memory task and repeated that analysis to identify the optimal latent brain state SH_WM415_ in the larger sample. In each cognitive task, we found that the state that best matched to SH_WM415_ was the same state that matched SH_WM_ (Supplementary Results and Supplementary Figs. S10–S13).

### Robustness of state matching with respect to reference tasks

To examine the robustness of our findings with respect to the choice of the reference tasks, we examined state matching using the HCP relational processing and SST tasks as reference tasks. In each cognitive task, we found that the state best matched to the optimal brain state was the same state that matched SH_WM_ (Supplementary Results and Supplementary Figs. S14–S17).

### Robustness of the main findings with respect to ROI selection

To examine the robustness of our findings with respect to the selection of ROIs, we used NeuroSynth to create a new set of ROIs based on meta-analysis of working memory and repeated the analyses to identify the optimal latent brain state in the HCP n-back task and labeled the optimal state as SH_META_. We then used BSDS and state-matching algorithms to determine the optimal brain state which matched SH_META_ in each cognitive task and determined whether the matched brain state was behaviorally significant. We found that, in each cognitive task, the state that best matched SH_META_ predicted task performance (Supplementary Results and Supplementary Figs. S18–S24).

### Discussion

We used a probabilistic dynamical systems model to investigate fundamental circuit mechanisms underlying the functioning of a multiple-demand system across multiple cognitive domains. Our computational modeling revealed a shared dynamic latent brain state engaged across diverse experiments and four data cohorts that have been widely used to investigate human cognition. Importantly, despite significant differences in experimental paradigms, data acquisition protocols, and participant cohorts, the temporal properties of brain states predicted cognitive task performance in each of the tasks. Moreover, the occurrence rates of the shared latent state also predicted behavioral performance. Furthermore, weak engagement of the shared brain state was related to inattention symptoms, suggesting that our generative model is also relevant for investigations of psychopathology. Together, our findings uncover a general dynamic brain state that is preferentially engaged during cognition, and demonstrates that functional circuits associated with the multiple-demand system can adaptively contribute to a wide range of cognitive functions^16^.

The first major goal of our study was to determine whether a shared brain state is engaged across a broad range of cognitive tasks within a common multiple-demand system. Meta-analyses of functional neuroimaging studies involving diverse cognitive tasks have reported the involvement of a common set of brain regions including insular, prefrontal, parietal and cingulate cortices^30,39,58,59^. A fundamental yet unaddressed question is whether common brain states are engaged across cognitive task domains. This question is especially challenging because cognitive tasks differ along many dimensions, they activate similar brain areas—identifying commonly activated brain areas does little to capture the underlying functional circuitry involved in these tasks. We used a machine learning algorithm to address this challenge.

A key aspect of our BSDS model is the identification of brain states based on joint modeling of activation and co-activation patterns^28^. Such models have the advantage that they do not depend on known relationships between neural activity and external experimental variables^21,22^. Our state space models jointly capture time-varying changes in activation and connectivity patterns across brain regions involved in cognitive control and, moreover, provide a rigorous computational approach for linking neural processes across two or more datasets. A key point to note here is that brain states inferred by our model reflect factors which can be explicitly modeled, such as experimentally-defined task conditions, as well as unobserved mental processes, such as a momentary lapse in attention, changes in motivation, alertness and fatigue which influence brain states^60–64^.

Crucially, generative model parameters derived by BSDS allowed us to map brain states across cognitive tasks and use state space dynamic measures for capturing shared features. In a previous study, we discovered an optimal brain state SH_WM_ associated with high-load cognitive demands during working memory^28^ and this served as a reference state for the present study. Crucially, we found that, in each of the seven other cognitive tasks, there was a shared brain state that had a highly similar profile as SH_WM,_ the optimal state in the working memory task.

Notably, correspondence between brain states across cognitive tasks was examined using two different analytic measures which yielded convergent findings (Fig. 1). The first approach was based on KL-divergence of multivariate latent variables independently derived from each task. In this formalism, alignment of brain states is captured by low KL-divergence between model parameters, including regional activation and inter-regional functional connectivity. Low KL-divergence reflects similar multivariate patterns of latent variables, indicating a strong match in brain states identified from the working memory task on the one hand, and each of the other tasks on the other.

A second, and much more challenging approach, was based on using generative aspects of the BSDS model. In this approach, model parameters associated with the reference state SH_WM_, the optimal *n*-back working memory task, were used as priors to estimate the posterior probabilities of SH_WM_ in each of the seven other cognitive tasks. Alignment of latent brain states was captured by a high correlation between temporal evolution of the brain states derived by applying BSDS independently or using model parameters associated with SH_WM_ from the working memory task. Remarkably, in each task, we found that brain states with high temporal correlation precisely matched the states derived using KL-divergence: the brain state that had the lowest KL-divergence also showed the highest temporal correlation. It is further noteworthy that our approach was able to detect a shared latent state in cognitive tasks that used block fMRI designs, but also in the case of five tasks that used fast event-related fMRI designs where the temporal evolution of brain states is much more complex and cannot be accurately estimated by conventional approaches.

In each of the seven cognitive tasks, BSDS captured common brain states better than conventional general linear models (Supplementary Fig. S9). While this common brain state is characterized by a high level of similarity in co-activation and functional connectivity patterns, each task has its own unique fingerprint. Identification of the common brain state across different cognitive tasks demonstrates that similar brain circuity is engaged when cognitive demands are high, regardless of the details of task context. Contrarily, when cognitive control is not needed, the multivariate pattern of activation and connectivity deviates from that in the shared high cognitive-load brain state. Moreover, these brain states fluctuate over time and are characterized by distinct task- and state-specific state transition probabilities.

The second major goal of our study was to investigate the behavioral relevance of brain states in each of the seven cognitive tasks. We tested the hypothesis that engagement of the shared latent state would be associated with better cognitive performance, and further isolate task-optimal brain states from non-optimal or task-irrelevant brain states. We examined the relationship between occurrence of latent brain state and behavioral performance using both multivariate and univariate approaches. Canonical correlation analysis revealed multivariate relations between brain states and behavioral measures in each of the seven cognitive tasks. Thus, the temporal pattern of dynamic fluctuations of brain states is highly behaviorally relevant.

Additionally, temporal properties of the shared brain state predicted performance in each of the seven tasks, highlighting the behavioral relevance of the shared brain state identified in the present study. Specifically, in each task, the occupancy rate of the shared brain state that matched SH_WM_, the optimal state in the *n*-back working memory task^28^, was significantly correlated with behavioral measures associated with cognitive control capacity specific to each task.

Each of the four DMCC tasks taps into a different aspect of cognitive control. The AxCPT is a widely used paradigm for probing dual mechanisms of proactive and reactive control^32^. We assessed proactive and reactive control by contrasting the “AY” and “BX” task conditions, as longer RT in “AY” is associated with implementation of proactive control and shorter RT in “BX” is associated with good reactive control^44,45^. A larger difference between “AY” and “BX” indicates greater proactive and reactive control. CCA revealed an association between brain state and behavioral measures. Aligned with CCA weight patterns, univariate analysis revealed that higher occupancy of the shared latent state SH_AxCPT_ was correlated with greater proactive and reactive control. Our findings suggest that optimal engagement of the shared latent state facilitates implementation of dual control processes.

The CuedTS allows investigation of cognitive control associated with response based on previously cued task rules^46,48^. This task has long been recognized as a critical paradigm to assess a core component of cognitive control—the ability to dynamically update task representations and configure attention and action systems for processing the upcoming target^31^. Incongruent trial conditions require participants to make cue-dependent decisions, whereas in congruent trials a response can be made independent of the cue. Good performance on incongruent trials relies on actively maintaining task-relevant stimulus-response association and inhibiting task-irrelevant stimulus-response association. CCA revealed the association between the high positive weight of SH_CuedTS_ and the positive weight of Incongruent Accuracy and negative weight of Incongruent RT. Aligned with CCA weight patterns, higher occupancy rate of the shared latent state SH_CuedTS_ was associated with greater cognitive control index (or reduced congruency effect). Our findings suggest that engagement of the shared latent brain state facilitates task preparation.

The Sternberg working memory task is a delayed match to sample paradigm, in which participants are presented with a set of stimuli to be remembered and then asked to judge whether a probe matches stimuli presented during the encoding phase^49,51^. The high load condition is particularly cognitively challenging because it requires participants to encode and maintain in memory a long list of stimuli (up to eight words). A composite measure based on both accuracy and reaction time during the high load condition was used to index cognitive control. CCA revealed an association between brain state and behavioral measures. Aligned with CCA weight patterns, we found a positive correlation between occupancy rate of the shared latent brain state SH_Stern_ and cognitive control, suggesting that the ability to engage SH_Stern_ predicts individual differences in cognitive control ability.

Finally, the Stroop task is a classic probe of conflict resolution and response inhibition^52^. CCA revealed an association between brain state and behavioral measures. Aligned with CCA weight patterns, occupancy rates of the shared brain state SH_Stroop_ were associated with the Stroop effect, suggesting that engagement of this state facilitates resolution of stimulus-response conflict.

In the SST, participants are required to withhold prepotent responses when presented with an infrequent stop signal. We applied the Race Model^37^ to compute SSRT, which provides a neurophysiologically validated estimate of stopping speed^65^. SSRT is a canonical measure of inhibitory control, and a core component of cognitive control. CCA revealed an association between brain state and behavioral measures. Aligned with CCA weight patterns, we found that the occupancy rate of SH_SST_ was correlated with SSRT, suggesting that the ability to engage SH_SST_ predicts individual differences in inhibitory control ability.

In the RP task, participants are required to first identify the dimensions along which stimuli differ in top and bottom rows of stimuli, and then determine whether the dimensions are the same or not. In comparison to the baseline matching control condition, in which participants determine which stimulus matches the target in a defined condition, the RP task requires extraction of abstract information from concrete external stimuli and maintenance of novel representations in working memory for decision-making based on task rules retrieved from long-term memory^66^. This task requires high-order cognitive control processes associated with establishing and switching task sets. CCA again revealed a multivariate relation between brain states and behavioral measures, and this relationship was further replicatd by univariate analysis. Critically, these findings were also replicated in across two different sessions, demonstrating the robustness and reproducibility of a task-optimal brain state during relational processing.

Our findings demonstrate that engagement of a shared task-general dynamic brain state predicts cognitive performance across diverse cognitive tasks. While each task differs in the specific cognitive processes engaged over time, they all require access to a core set of neural resources to meet increased cognitive challenges^30,58,67^. Our findings contribute to the understanding of the functioning of a multiple-demand system that was proposed based on a common set of brain regions activated during various cognitive tasks^16^. The existence of a common underlying latent process governing this system was previously unknown. Our findings shed light on this critical issue and reveal a common brain state that is activated across diverse cognitive control tasks, and its dynamic properties predict task performance regardless of task context. More generally, isolating task-optimal brain states from non-optimal or task-irrelevant brain states associated with cognitive performance provides information about dynamic neurocognitive processes engaged by a shared multiple-demand frontoparietal cortical system.

The third major goal of our study was to investigate generalizability of the multiple-demand system and shared brain states to a developmental cohort. We further sought to determine relevance to inattention symptoms associated with ADHD, a  prevalent neurodevelopmental disorder characterized by deficits in cognitive control^35,36,68–70^. Brain imaging studies have linked the disorder with abnormalities in activation and connectivity of frontoparietal cortical regions^71–77^. However, it is not known whether brain state models associated with a multiple-demand frontoparietal system derived from healthy adults are generalizable to children, and whether the temporal properties of a shared task-related brain state also predict cognitive deficits and clinical symptoms associated with ADHD. To address this, we used a stop signal task, a classic behavioral paradigm that has been widely used to investigate inhibitory control^37^, to investigate individual differences in children’s inhibitory control ability^78^, as well as inattention, a hallmark of cognitive deficits in children with ADHD^79^.

Our analysis revealed three key findings. First, our computational model showed that brain states are generalizable from adults to children. Our prior research on the developmental maturation of response inhibition in the stop signal task demonstrated that the extent to which children engage an adult-like template of multivariate brain activation patterns predicts their behavioral performance^8^. Building on these findings, we show here that the shared latent brain state present in adults across a wide range of cognitive control tasks is also present in children during the stop signal task. This suggests that children engage a shared dynamic process, not just similar brain activation patterns^8^. Second, we found a significant relation between SSRT, the model-based estimation of stopping speed, and the occupancy rate of SH_SST_, suggesting that weaker engagement of the shared latent state is related to weaker inhibitory control in children. Third, we found that lower occurrence of the task-optimal shared state was also related to the clinical symptoms of inattention. The identification of a shared latent brain state between children and adults provides a valuable template and analytic model for probing aberrant brain circuit dynamics underlying cognitive control and attentional deficits within the framework of a core multiple-demand system.

Each brain state is characterized by a distinct pattern of multivariate activity and inter-regional connectivity^28^. To determine the unique contributions of each brain region to the matched latent states from each task, we conducted a leave-one-ROI-out analyses and examined multivariate features of each brain region in relation to its impact on the similarity between SH_WM_ and SH from each task. Strikingly, multivariate features from the dorsolateral prefrontal cortex, in left and right MFG, exhibited the greatest similarity between SH_WM_ and SH in other cognitive control tasks. The dorsolateral prefrontal cortex is a crucial region underlying executive control functions in both human^13,16,39,80^ and non-human primates^81–83^. Furthermore, meta-analytic research of neuroimaging studies has found that the dorsolateral prefrontal cortex is a commonly activated region in a variety of different cognitive and attentional control tasks^30,39,58,84^, and is thought to be a subserve domain-general cognitive functions. Here, our findings reveal a central role for the dorsolateral prefrontal cortex in operation of the multiple-demand system not only during working memory, but also in the shared state engaged during a wide range of tasks that require cognitive control.

Although the relational processing (RP) task encompasses more complex cognitive operations beyond those employed by the canonical cognitive control task, BSDS still identified a shared latent state that matches the optimal latent brain state in the n-back working memory task. Given that SH_WM_ is a task-general dynamic brain state underlying cognition, an interesting question is whether engaging SH_WM_ during cognitive and attentional control tasks represents an individual’s unique latent dynamic neurobiological profile. To probe this question, we examined the occupancy rate of SH_WM_ during the n-back working memory task, and the occupancy rate of SH_RP_ during the relational processing task, from the same group of participants. Indeed, there was significant positive correlation between SH_wm_ and SH_RP_ across participants. This suggests that an individual who is more likely to engage SH_WM_ during a working memory task is also more likely to engage this shared brain state during other cognitive and attentional control tasks. Furthermore, by replicating this finding in two sessions of the RP task, we demonstrated the robustness of this finding. These results reveal a shared latent brain state across cognitive tasks within an individual, and suggest the potential for identifying individualized measures arising from dynamic brain processes across multiple cognitive tasks.

While our study presents critical insights into the dynamics of cognitive control and attention across seven different tasks, there are limitations warrant consideration and suggest avenues for future work. First, the task fMRI data primarily came from the Human Connectome and the Dual Mechanisms of Cognitive Control projects, generalizability to other datasets and populations needs to be examined. Second, our choice of Automatic Relevance Determination priors for high-dimensional variable selection was motivated by computational tractability and prior validation^28^ and by their broader use in the modern machine learning literature^85–87^. Nevertheless, it is important to acknowledge that the selection of priors and model inference in Bayesian modeling remains an area of active debate within the field. Alternative approaches, such as spike-and-slab priors^87,88^, may offer valuable insights and should be considered in future investigations. Third, our methodology was rigorously validated in previous research, including simulations and optogenetic stimulation, underscoring its reliability^28^. However, the neuroscience community has yet to reach a consensus regarding ideal biophysically realistic models for validating causal circuit dynamics. Future investigations should further validate state space models, such as ours, against a broader spectrum of biophysically-realistic simulations, extending beyond the scope of those explored in our prior work^28^.

Human cognition relies on dynamic brain mechanisms for implementing adaptive cognitive functions. Our study reveals latent brain mechanisms underlying the operation of a multiple-demand system across a wide range of cognitive task domains, and help shed light on a major unsolved problem in cognitive neuroscience. Our findings provide critical insights into dynamic brain mechanisms underlying human cognition, and our generative hidden Markov model-based computational framework opens promising avenues for probing neurocognitive function, as well as their disruptions in psychiatric and neurological disorders.

## Methods

### Ethics statement

Data acquisition for the Dual Mechanisms of Cognitive Control (DMCC) was approved by the Institutional Review Board of The Washington University in St. Louis. Data acquisition for the stop-signal task (SST) was approved by the Institutional Review Board of Stanford University. Data acquisition for the Human Connectome Project (HCP) was approved by the Institutional Review Board of The Washington University in St. Louis. Informed consent was obtained from all the participants or their legal guardians.

### Bayesian switching dynamical systems (BSDS) model

Bayesian switching dynamical systems (BSDS) is a powerful state-space generative model for uncovering latent brain state dynamics that may not necessarily be time-locked to experimental task conditions^28,41,89,90^. BSDS identifies brain states and their dynamic spatiotemporal properties in an optimal latent subspace. These properties allowed us to identify shared brain states across multiple cognitive tasks. In each task, the initial value for the number of possible latent brain states was set as 15. BSDS uses an Automatic Relevance Determination algorithm which regularizes the solution space and effectively prunes away redundant or superfluous features^28,91^ Details of the method are described in our previous study^28^ and in the Supplementary Method.

### BSDS analysis with priors

BSDS can be used to model latent brain states in new data using priors from model parameters from previously trained data. The BSDS model contains two sets of latent variables: global and local. Global latent variables are not a function of data instance (all variables in Equations 1–2 that are not a function of time). Local latent variables in BSDS on the other hand are a function of time and data sample. The main difference between these two variables is that the global variables can be transferred from one data to another. The local variables, however, need to be learned again for new data.

When using an existing BSDS model to fit the new data and estimate posterior probability of the states (latent state variables) in the new data, the global variables are directly transformed from the existing BSDS model and the local variables are learned using the new data given the global variables. In practice, only a single iteration of BSDS would suffice to learn the local latent variables for the new data.

### BSDS state matching

We developed two metrics to determine how well brain states are matched across tasks: state space closeness and state temporal closeness.

*State space closeness* Each brain state was defined by multivariate features in optimized latent space, activation level and covariance. Let S1_WM_ to S4_WM_ denote dynamic brain states obtained by applying BSDS to the HCP *n*-back task. We showed identified state S1_WM_ which dominated the 2-back high-load (SH) condition and predicted performance and decision-making process in the 2-back trials^28^. To simplify state names, without loss of generalization, we relabel S1_WM_ as SH_WM_. Now let S1_AxCPT_ to SN_AxCPT_ denote dynamic brain states obtained by applying BSDS to the AxCPT task. Each brain state is represented by a Gaussian distribution with mean and covariance associated with multivariate features in the latent brain state. The state space closeness (denoted as ***c***) is computed by one divided by the Kullback–Leibler divergence (KLD) between two states’ Gaussian distributions. Higher closeness or smaller KLD indicates that the two states are more similar in their multivariate features. If S1_AxCPT_ has the highest state space closeness with SH_WM_ among all the states obtained in the AxCPT task, S1_AxCPT_ is considered a state matching SH_WM_ and relabeled as SH_AxCPT_.

*State temporal closeness* Given the latent state model, ROI timeseries can, in turn, be represented as posterior probability time courses of estimated brain states. To confirm that SH_AxCPT_ is a state matching SH_WM_, we further examined whether SH_WM_ and SH_AxCPT_ have similar temporal evolutions if two different latent state models are fit to the same data. Let S1_AxCPT_ to SN_AxCPT_ denote dynamic brain states obtained by applying BSDS to the AxCPT task. BSDS estimates temporal posterior probability (TPP) of each brain state at each time point in the AxCPT task. TPP(S1_AxCPT_)_AxCPT_ to TPP(SN_AxCPT_)_AxCPT_ denote temporal posterior probability of S1_AxCPT_ to SN_AxCPT_ in the AxCPT task.

We then applied global model parameters associated with SH_WM_, S2_WM_, S3_WM_, and S4_WM_ from the HCP WM task to the AxCPT task to compute temporal posterior probability of latent brain state of the HCP WM task in the AxCPT task. Let TPP(SH_WM_)_AxCPT_ denote temporal posterior probability of SH_WM_ in the HCP AxCPT task. State temporal closeness (denoted as ***r***) is measured by the Pearson’s correlation between each pair of temporal posterior probabilities in the TPPs from two task. We hypothesize that, if SH_AxCPT_ and SH_WM_ have the highest state space closeness, TPP(SH_AxCPT_)_AxCPT_ and TPP(SH_WM_)_AxCPT_ should have the highest state temporal closeness.

This process was repeated for each of the seven cognitive tasks to identify states that best matched SH_WM_ in each task.

*Statistical significance of space closeness* We used permutation testing in which space closeness was computed across two randomly drawn states from the n-back working memory task and the cognitive control task (e.g., AxCPT). The two states were selected from the same or different tasks, which is a random process. This permutation was repeated 100 times to generate a distribution of space closeness from which the significance (*p* value) of the space closeness between SH_WM_ and SH of another task, e.g., SH_AxCPT_, was computed. The permutation was performed only 100 times because of the limited number for random combination of states and preclude inflation of *p* values.

*Statistical significance of temporal closeness* We used the same permutation procedure as described above to compute temporal closeness between two randomly selected brain states. The permutation was repeated from 100 times to generate a distribution of temporal closeness from which the *p* value of the temporal closeness between SH_WM_ and SH of another task, e.g., SH_AxCPT_, are computed.

### Temporal and spatial metrics of dynamic brain states

Measures extracted from BSDS include occupancy rate and temporal evolution of latent brain state, and mean and covariance of states.

### Leave one ROI out analysis on state space closeness

To evaluate the critical contribution of each brain region to the similarity of brain states between tasks, we conducted a leave one ROI out analysis. Each time, we removed all the features from one ROI in the state space in SH_WM_ and SH_X_ from another task X (e.g., SH_AxCPT_) and computed the change (delta) of the KLD before and after removing the ROI. Then, the impact of the lesion on the state similarity was quantified using 1/delta. We repeated the procedure for all the ROIs in each task. The larger with 1/delta value, the greater contribution the ROI has on the similarity between brain states. Statistical significance estimation is described in Supplementary Methods.

### Human fMRI datasets: HCP dataset

N-back working memory task We used the high-load dynamic brain state from the HCP N-back task identified from our previous study^38^, as the reference state for all other cognitive tasks in the present study. The same sample (122 individuals) from the previous study was used in the current study.

Relational processing (RP) task We selected 90 individuals from the 122 who had also participated in the *n*-back study^38^. The following criteria were used: (1) complete behavioral and brain imaging data in two different acquisition sessions; (2) range of head motion in any translational and rotational direction less than 1 voxel; (3) average scan-to-scan head motion less than 0.25 mm.

Task details are described in Supplementary Methods and Supplementary Fig. 1.

### Human fMRI datasets: DMCC dataset

We used the Dual Mechanism of Cognitive Control (DMCC) dataset^31^ which contains four different task paradigms for probing cognitive control: (1) AX continued performance task (AxCPT), (2) Cued task switching task (CuedTS), (3) Sternberg working memory task (Sternberg) and (4) Stroop interference task (Stroop). We used data from 50 individuals (19–42 years old, 31 F/19 M) out of a total of 89 participants in the DMCC dataset based on the following criteria: (1) complete behavioral and brain imaging data; (2) range of head motion in any translational and rotational direction was less than 1 voxel in all the tasks; (3) average scan-to-scan head motion was less than 0.25 mm in all the tasks.

Task details are described in Supplementary Methods and Supplementary Fig. 1. Additional details can be found in online descriptions of the DMCC^31,92^.

### Human fMRI datasets: Stanford dataset

Stop-signal task (SST) Forty-five children with ADHD or TD children (9–12 years old, 22 F/23 M) completed the SST task during MRI scanning. Task details are described in Supplementary Methods and Supplementary Fig. 1.

Clinical symptoms of Inattention were assessed using the Strengths and Weaknesses of ADHD-symptoms and Normal-behavior (SWAN) rating scale^93^.

### Task performance statistical test

Details are described in Supplementary Methods.

### fMRI acquisition

DMCC Functional scans were acquired on a 3 T Siemens Prisma with a 32-channel head coil, without in-plane acceleration (iPat = none). CMRR multiband sequences were used, TR = 1200 ms, TE = 33 ms, flip angle = 45°, in-plan resolution = 2.4 mm and multiband factor = 4.

SST fMRI data were acquired on a 3 T GE Signa scanner using a 32 channel head coil at the Richard M Lucas Center for Imaging at Stanford. Functional images of 42 axial slices were acquired using the multiband gradient-echo planar imaging with the following parameters: TR = 490 ms; TE = 30 ms; flip angle = 45°, in-plane resolution = 3 mm and multiband factor = 6.

HCP fMRI data were acquired using a multiband, gradient-echo planar imaging with the following parameters: TR = 720 ms, TE = 33.1 ms, flip angle = 52°; in-plane resolution = 2 mm and multiband factor = 8.

### fMRI preprocessing

For the four DMCC tasks and the HCP *n-*back and RP tasks, we downloaded minimally preprocessed fMRI data. DMCC data was preprocessed using *fMRIPrep pipeline*^31^, including head motion correction, correction of susceptibility-derived distortion, slice-timing correction, registration and normalization. HCP data was preprocessed using *fMRIVolume pipeline*^94^, including correction of gradient-nonlinearity-induced distortion, realignment for motion correction, registration, and normalization in 2 mm MNI space. Details of the preprocessing steps in DMCC and HCP datasets are described in previous studies^31,94^. We applied spatial smoothing with a Gaussian kernel of 6 mm FWHM in the minimally preprocessed DMCC and HCP data to improve signal-to-noise ratio as well as anatomical correspondence between individuals^28^.

For the SST, fMRI data were preprocessed using SPM12, including realignment, slice-timing correction, co-registration, normalization and smoothing^8^.

The same smoothing parameters were used in the HCP, DMCC and SST datasets.

### Region of interest (ROI) and time series

Load-dependent ROIs were determined using the HCP n-back task on the contrast of interest: 2-back versus 0-back, including 9 load-positive (2-back > 0-back) ROIs: bilateral anterior insula (AI), bilateral middle frontal gyrus (MFG), bilateral frontal eye field (FEF), bilateral intraparietal sulcus (IPS) and dorsomedial prefrontal cortex (DMPFC), and 2 load-negative (2-back <0-back) ROIs: ventromedial prefrontal cortex (VMPFC) and posterior cingulate cortex (PCC)^28^ (Supplementary Fig. S2a). Each ROI was a 6-mm radius sphere centered at the corresponding peak voxel. See Supplementary Table S12 for MNI coordinates of the ROIs.

Time series of the 1st eigenvalue was extracted using Marsbar (https://marsbar-toolbox.github.io/) from each ROI per subject in each dataset. A multiple linear regression approach with 6 realignment parameters (3 translations and 3 rotations) was applied to time series to reduce head-motion-related artifacts and resulting time series was further linearly detrended and normalized.

### Relation between brain states and behavioral performance

To characterize the relation between brain states and behavioral performance in different tasks, we first used multivariate canonical correlation analysis (CCA) to investigate multivariate relations between brain state and cognitive performance measures in each task^95,96^. The robustness of the CCA model was also tested using a predictive model with leave-one-out cross validation. Details of the CCA variable setting and prediction analyses are described in Supplementary Methods. We then used *Pearson*’s correlation (two-tailed) to investigate the link between the occupancy rate of the shared multiple-demand state and task-specific measures of cognitive control abilities (Supplementary Table S9).

### Relation between brain states and clinical symptoms

To characterize the relation between the shared multiple-demand brain state and attentional deficits, we used *Pearson’s* correlation (two-tailed) to examine the relation between occupancy rate of SH_SST_ and the severity of inattention symptoms in children assessed using the SWAN rating scale.

### Reporting summary

Further information on research design is available in the Nature Portfolio Reporting Summary linked to this article.

### Supplementary information

Supplementary Information

Reporting Summary

### Source data

Source data

## Data Availability

All original data reported in this study are publicly available on Zenodo: https://zenodo.org/records/10702914. Source data are provided with this paper.

## Code availability

Functional MRI data preprocessing and statistical analyses were performed on the SPM12 and FSL 6, and Matlab 2020. BSDS and State Matching Code can be accessed at Github (https://github.com/scsnl/Cai_Multiple_Demand_System_2023) and Zenodo (10.5281/zenodo.10660076)^97^.
