# Supplementary material for: A multi-demand operating system underlying diverse cognitive tasks
Source: Nat Commun. 2024 Mar 11;15:2185. doi: 10.1038/s41467-024-46511-5 (PMC10928152; doi:10.1038/s41467-024-46511-5)
Supplement: Supplementary file 1 — Supplementary Information [file 41467_2024_46511_MOESM1_ESM.pdf]

Supplementary Information

A multi-demand operating system underlying diverse cognitive tasks

Weidong Cai[1,2], Jalil Taghia[3], Vinod Menon[1,2,4]

(1) Department of Psychiatry & Behavioral Sciences
Stanford University School of Medicine
Stanford, CA, United States

(2) Wu Tsai Neuroscience Institute
Stanford University
Stanford, CA, United States

(3) Department of Information Technology
Uppsala University
Uppsala, Sweden

(4) Department of Neurology & Neurological Sciences
Stanford University School of Medicine
Stanford, CA, United States

Content

I. Supplementary Methods
   a. fMRI datasets
   b. State space modeling using BSDS
   c. Statistical significance in leave one ROI out analysis
   d. General linear model analysis of task-related activation
   e. gPPI analysis of task-related functional connectivity
   f. Comparison of state space models with conventional GLM and gPPI analyses
   g. Canonical correlation analysis and prediction model
   h. Univariate brain-behavior correlation analysis
   i. Power analysis

II. Supplementary Results
   a. Statistical significance of state matching
   b. Statistical significance of MFG role in leave one ROI out analysis
   c. Robustness of state matching with respect to sample size of the N-back task
   d. Robustness of state matching with respect to reference cognitive tasks
   e. Robustness of the main findings with respect to ROI selection

III. Supplementary Figures

IV. Supplementary Tables

V. Supplementary references

I. Supplementary Methods

A. fMRI datasets

HCP datasets

N-back working memory task We used the high-load dynamic brain state from the HCP N-back task identified from our previous study [1], as the reference state for all other cognitive tasks in the present study. The same sample from the previous study was used in the current study. We selected 122 individuals (ages: 22-36 years old, 79 female/43 male) from 500 subjects (HCP Q1-Q6 Data Release) based on the following criteria: (1) range of head motion in any translational and rotational direction less than 1 voxel; (2) average scan-to-scan head motion less than 0.25 mm; (3) performance accuracy greater than 50% in each task block and session; and (4) right handedness.

The HCP N-back working memory task combines the category specific representation task and the *n*-back working memory task in a single task across two sessions. Participants were presented with blocks of trials that consisted of pictures of faces, places, tools and body parts. Within each session, the 4 different stimulus types were presented in separate blocks. Furthermore, within each session, half of the blocks are 2-back working memory and half are 0-back working memory task. In the 2-back working memory task blocks, subjects were requested to determine whether the current stimulus matches the stimulus in two presentations of stimuli prior within the same block. In the 0-back working memory task blocks, subjects were requested to determine whether the current stimulus matches the target that was presented in the beginning of each block (cue). A 2.5 second cue indicated the task type (and target for 0-back task) at the beginning of each block. Each task session contained 8 task blocks (10 trials of 2.5 seconds each, for 25 seconds) and 4 fixation ("rest") blocks (15 seconds). On each trial, the stimulus was presented for 2 seconds, followed by a 0.5 second inter-trial-interval (ITI).

Relational processing (RP) task We selected 90 individuals from the 122 who had also participated in the *n*-back study [1]. The following criteria were used: (1) complete behavioral and brain imaging data in two different acquisition sessions; (2) range of head motion in any translational and rotational direction less than 1 voxel; (3) average scan-to-scan head motion less than 0.25 mm.

The RP task involved a relational processing task and a control condition. In the relational processing condition, participants were presented two pairs of objects, with one pair at the top of the screen and the other pair at the bottom. Participants were required to determine whether the top pair of objects and the bottom pairs of objects are different in the same dimension or not. For example, the top pair differed in texture and the bottom pair differs in shape. In the control task, there were two objects presented at the top of the screen, one object at the bottom of the screen and a word in the middle to indicate dimension (e.g.ge. "shape" or "texture"). Participants were told to judge whether the bottom object matches either of the top objects in that dimension. Each condition had 3 blocks and each block lasts 18 seconds. There were 4 trials in each relational processing block and 5 trials in each control matching block. In the relational condition, the stimuli were presented for 3500 ms, with a 500 ms ITI. In the matching condition, the stimuli were presented for 2800 ms, with a 400 ms ITI.

DMCC dataset

We used the Dual Mechanism of Cognitive Control (DMCC) dataset [2] which contains four different task paradigms for probing cognitive control: (1) AX continued performance task (AxCPT), (2) Cued task switching task (CuedTS), (3) Sternberg working memory task (Sternberg) and (4) Stroop interference task (Stroop). We used data from 50 individuals (19-42 years old, 31F/19M) out of a total of 89 participants in the DMCC dataset based on the following criteria: (1) complete behavioral and brain imaging data; (2) range of head motion in any translational and rotational direction was less than 1 voxel in all the tasks; (3) average scan-to-scan head motion was less than 0.25 mm in all the tasks.

AxCPT AxCPT is a continuous performance task which includes 4 different standard (Go) trial types: AX, AY, BX, and BY, and No-Go stimuli. Participants were presented with the letters A or B followed by letters X or Y (or not "X"), comprising AX, AY, BX, and BY pairs. They are asked to respond to the probe ("X") only if it followed the contextual cue ("A"). Participants were to make another response to other cue–probe sequences ("A" then "Y," "B" then "X," or "B" then "Y"), each occurring with much lower probability than the target pair ("AX"). The proportions of trial types were set to ensure equal frequencies of A-cue and B-cue trials. There were 40% AX, 10% AY, 10% BX and 40% BY trials. No-Go stimuli occurred with low frequency (16.7%). Participants were instructed to withhold response on these trials. Presentation time of cues and probes was 500 msec. Probes were accompanied by a white rectangular border, presented 250 ms prior to probe onset. The cue-probe delay interval was 4 seconds, placing demand on goal (context) maintenance processes. The full condition includes 216 trials (72 AX, 18 AY, 18 BX, 72 BY, 18 A-No-Go, 18 B-No-Go).

CuedTS Participants performed a letter-digit task in which they were cued to respond to either the letter or number in target stimuli which consisted of a letter-digit pair (e.g., "D 3", or "1 A"). Based on the cue, participants had to either categorize the letter as a vowel or consonant, or categorize the digit as even or odd depending on the cue. The Cued task switching task consists of sixteen different stimuli (1A, A1, 2A, A2, 1B, B1, 2B, B2, 3E, E3, 4D, D4, 5H, H5, 6I and I6). Each trial began with a 300 ms fixation cue ("+") in the center of the screen. Following the fixation is a 500 ms task cue ("attend number"/"attend letter") presented in red. If the cue said "Attend Number", participants decided if the number is odd or even. If the cue said "Attend Letter", participants decided whether the letter was a consonant or vowel. A 4000 ms CTI followed. In incongruent trials, the two stimuli activated competing cue-dependent responses (e.g. "A 3") whereas in congruent trials, the response was cue-independent (e.g. "A 2"). Within each scanning run, 3 task blocks alternated with 4 resting fixation blocks (30 sec duration); in each task block, the inter-trial interval was varied randomly (across 3 step sizes).

Sternberg task In this task participants determined whether a probe matches a list of stimuli presented previously. High and low load conditions differed in the number of stimuli that need to be maintained in working memory. The Sternberg working memory task included 4 different memory set list-lengths (5, 6, 7 and 8 items). The full condition included 90 trials. The proportions were 5-item: 40%, 6-item: 10%, 7-item: 20%, 8-item: 30%. The 5-item and 6–item trials denoted low load (LL) trials and the 7-itme and 8-item trials denoted high load (HL) trials. The retention interval was 4 seconds after which a probe was presented. On half the trials the probe matched one of the stimuli presented during the encoding period.

Stroop task The Stroop Interference task is a color-word task, in which stimuli are words presented in different colored fonts and participants are asked to name the color of the font in which the words are presented. The task included congruent and incongruent trials. In congruent trials, the word name and font color were the same (e.g., the word RED presented in

red font). In incongruent trials, the word name and font color were different (e.g. the word RED presented in green). The full condition includes 72 congruent and 144 incongruent trials.

Stanford dataset

Stop-signal task (SST) Forty-five children with ADHD or TD children (9-12 years old, 22F/23M) completed the SST task during MRI scanning. Each participant performed two runs of the SST with 96 trials per run. Participants were instructed to respond as quickly as possible to green arrows (Go Signal) with their right pointer or middle finger based on the direction of the arrow. In 33% of the trials, after a variable delay, the green arrow turned red (Stop Signal), indicating that the participant should cancel their (prepotent) response. The delay between the Go Signal and the Stop Signal, the SSD varied across trials in a step-wise fashion and was adjusted dynamically to individual performance: beginning at 165ms, it decreased by 33ms for a failed stop, and increased by 33ms for a successful stop.

Task performance statistical test

The effect of cognitive control load was tested using paired t-test between AY and AX, BY and BX conditions in the AxCPT; between Incongruent and Congruent conditions in the CuedTS; between high and low load conditions in the Stern; between Incongruent and Congruent conditions in the Stroop; between relational and match conditions in the RP (Supplementary Table S2 and S6). Data met the assumptions of the statistical tests, including normality and equal variances.

B. State space modeling using BSDS

BSDS generative model

Here we briefly describe the BSDS model [3]. Let $\boldsymbol{y}_t^s$ denote a D-dimensional vector of observed fMRI measurements in time t and for subject s. Further, let $\boldsymbol{z}_t^s$ denote a 1-of-K discrete vector of latent state variables of a hidden Markov model (HMM) with elements $z_{kt}^s, \forall k = 1, \dots, K.$ Two consecutive time instances are dependent via a first-order Markov chain through an HMM. Specifically, probability distribution of $\boldsymbol{z}_t^s$ depends on the state of the previous latent variable $\boldsymbol{z}_{t-1}^s$ through a conditional distribution $p(\boldsymbol{z}_t^s \mid \boldsymbol{z}_{t-1}^s, \boldsymbol{A}) = \prod_{k=1}^K \prod_{j=1}^K A_{jk}^{z_{t-1,j}^s z_{tk}^s}$ for all $t > 1$ represented by the transition probabilities $\boldsymbol{A},$ where $A_{jk} \equiv p\left(z_{tk}^s = 1 \mid z_{t-1,j}^s = 1\right)$, and a marginal distribution $p(\boldsymbol{z}_1^s \mid \boldsymbol{\pi}) = \prod_{k=1}^K \pi_k^{z_{1k}^s}$ represented by a vector of initial probabilities $\boldsymbol{\pi}$ where $\pi_k \equiv p(z_{1k}^s = 1)$ [4]. Next, we assume that at a given mode of the system given by the latent state $z_{kt}^s = 1$, observed vector $\boldsymbol{y}_t^s$ is generated via a state space model in form of

Equations 1-2:

$$\boldsymbol{y}_t^s = \boldsymbol{U}_k \boldsymbol{x}_{kt}^s + \boldsymbol{\mu}_k + e_{kt}, \qquad \forall t \mid z_{kt}^s = 1, \tag{1}$$
$$\boldsymbol{x}_{kt}^s = \overline{\boldsymbol{X}}_{kt}^s \vec{\boldsymbol{V}}_k + \boldsymbol{\epsilon}_{kt}, \qquad \forall t \mid z_{kt}^s = 1. \tag{2}$$

Line 1 of the model represents a probabilistic factor analysis model [5, 6] where $\boldsymbol{U}_k$ is a $D \times P$ dimensional linear transformation matrix that transforms data to a subspace of lower dimensionality, $P < D$, described using a P-dimensional vector of latent space variables $\boldsymbol{x}_{kt}^s$ mediated by an overall bias $\boldsymbol{\mu}_k$ and a measurement noise $\boldsymbol{e}_{kt} \sim \mathcal{N}(\boldsymbol{0}, \boldsymbol{\Psi}_k)$. Line 2 represents an autoregressive (AR) process of order R defined on the latent space variables of the factor

analysis model [7]. $\vec{\boldsymbol{V}}_k$ is a vector of AR coefficients. $\overline{\boldsymbol{X}}_{kt}^{s} = \mathrm{diag}(\overline{\boldsymbol{x}}_{kt}^{s})$ is a block diagonal isotropic matrix with elements of $\overline{\boldsymbol{x}}_{kt}^{s} = ({\boldsymbol{x}_{k,t-1}^{s}}^{\mathrm{T}}, {\boldsymbol{x}_{k,t-2}^{s}}^{\mathrm{T}}, \ldots, {\boldsymbol{x}_{k,t-R}^{s}}^{\mathrm{T}})$ representing latent space variables from the previous R time frames where T indicates the transpose operator. $\boldsymbol{\epsilon}_{kt} \sim \mathcal{N}(\boldsymbol{m}_k, \boldsymbol{\Sigma}_k)$ models the remaining error term in latent space. An AR process of a first order, $R = 1$, is defined on the representations of the observations in the latent subspace, $\boldsymbol{x}_{kt}^{s}$. Variables $(\boldsymbol{\Psi}_{\boldsymbol{k}}, \vec{\boldsymbol{V}}_k, \boldsymbol{m}_k, \boldsymbol{\Sigma}_k)$ are global latent variables which are not a function of time t. Detailed theoretical derivations are provided in our previous study [3].

Advantages of BSDS modelling

The BSDS model has the following key advantages over other approaches:
First, BSDS does not require arbitrary sliding windows, nor does it impose temporal boundaries associated with predefined task conditions – this is contrast to previous approaches for characterizing dynamic brain connectivity in fMRI data which rely on *ad hoc* procedures for determining critical parameters, such as the window length and number of brain states, which are known to greatly influence the estimation of dynamic brain states and connectivity [8]. In contrast, BSDS uses a Bayesian framework to automatically regulate model complexity and directly estimates the optimal number of latent states.

Second, BSDS applies HMM to state space variables of the observed fMRI data, resulting in a parsimonious model of generators underlying the observed data. BSDS applies HMM to latent space variables generated by an autoregressive process, resulting in greater robustness in state identification.

Third, BSDS allowed us to uncover brain states and their dynamic spatiotemporal properties, including probability and sequence of state transitions as well as inter-regional functional connectivity associated with each brain state, in an optimal subspace. In our previous study [3], we demonstrated that BSDS is robust to noisy and abrupt local changes in fMRI, resulting in more accurately identifying brain states and their temporal dynamic properties than conventional data driven approaches.

Finally, a novel aspect of our study is its use a generative model of brain dynamics derived one task to identify similar states in other tasks. Our generative model allows us to investigate the correspondence between brain states across tasks and test the hypothesis that a latent brain state that is optimal for behavioral performance during working memory [3] also occurs in other cognitive tasks, and predicts behavior.

C. Statistical significance in leave one ROI out analysis

To evaluate the statistical significance of leave one ROI out effect, we build a distribution of leave one ROI out effects from all brain states, from which the significance level (*p* value) for the effect of leave one ROI out in each ROI in the SH state (e.g. $\mathrm{SH}_{\mathrm{AxCPT}}$) was obtained. An FDR correction (p<0.05) was applied to correct for multiple comparisons.

D. General linear model analysis of task-related activation

A general linear model (GLM) analysis was used to determine task-related activation in each of seven cognitive control tasks [9]. Six motion parameters were entered as covariates of no interest, and both canonical hemodynamic response function (HRF) and its time-derivative were used to convolve the stimulus function to form task regressors.

AxCPT included seven task regressors: ANG, BNG, AX, AY, BX, BY and error. The contrast of interest is AY versus AX.

CuedTS included three task regressors: Congruent, Incongruent and error. The contrast of interest is Incongruent versus Congruent.

Sternberg included three task regressors: Low load, High load and error. The contrast of interest is HL versus LL.

Stroop included three task regressors: Congruent, Incongruent and error. The contrast of interest is Incongruent versus Congruent.

SST included four task regressors: Go Correct (Go), Go Error, Successful Stop (SuccStop), and Unsuccessful Stop (UnsuccStop). The contrast of interest is SuccStop versus Go.

RP sessions 1 and 2 included three task regressors: Match, Relational Processing (Relation) and error. The contrast of interest is Relation versus Match.

Task-related activation for each contrast of interest was extracted in each ROI used in the BSDS analysis: bilateral AI, MFG, FEF, IPL and DMPFC, PCC and VMPFC.

E. gPPI analysis of task-related functional connectivity

Seed-based generalized psychophysiological interaction (gPPI) was used to determine task-related functional connectivity [10]. Seeds were placed in the bilateral AI, MFG, FEF, IPL and DMPFC, the same ROIs used in the BSDS analysis. The gPPI model consisted of a physiological variable (the raw time series of a seed), multiple psychological variables (hemodynamic response function convolved main effect of condition of interest), and multiple interaction variables (deconvolved raw time series of the seed multiplied by main effect of condition of interest, and then convolved with the hemodynamic response function). Task-related connectivity was computed for each ROI and contrast of interest resulting in an 11x11 matrix with each column representing a seed and each row representing a target.

F. Comparison of state space models with conventional GLM and gPPI analyses

In each of the seven cognitive tasks, we examined whether state-space models generated better model fits with the reference *n*-back working memory task than conventional GLM [9] and gPPI [10] analyses. For state-space models, we assessed the similarity in mean activity and covariance patterns between SH_{WM} and the best matched brain state SH_X in each task *X* using *Pearson*'s correlation. Similarly, in the case of conventional GLM and gPPI analyses, the similarity in activation/deactivation and gPPI connectivity patterns between the *n*-back working memory task and each of the seven other cognitive control tasks was determined using *Pearson*'s correlation.

G. Canonical correlation analysis and prediction model

To characterize the relation between brain states and behavioral performance in different tasks, we first used canonical correlation analysis (CCA) to investigate multivariate relations between brain state and cognitive performance measures in each task [11, 12]. In each cognitive task, the occupancy rates of the latent brain states were the set of X variables and behavioral measures

were the set of Y variables. For the AxCPT, behavioral variables included RT in AX, AY, BX and BY trials. For the CuedTS, behavioral variables included accuracy and RT in congruent and incongruent trials. For the Sternberg, behavioral variables included accuracy and RT in high-load and low-load trials. For the Stroop, behavioral variables included accuracy and RT in congruent and incongruent trials. For the SST, behavioral variables included stop accuracy, unsuccessful stop RT, stop-signal delay and stop-signal reaction time. CCA was implemented using python scikit-learn package (https://scikit-learn.org/stable/modules/generated/sklearn.cross_decomposition.CCA.html).

A prediction model based on the CCA was examined using leave-one-out cross validation procedure such that one subject's data was used as the test set and the rest of subjects' data were used as the training set. The training set was then used to train a canonical correlation model and weights from the trained model were applied on the test set to generate predicted canonical variables of X and Y in the test set. This produce repeated N times (N is the sample size) such that each subject's data was used exactly once as a test set. The model performance was evaluated using correlation between predicted canonical variables of X and predicted canonical variables of Y across all the subjects.

H. Univariate brain-behavior correlation analysis

We used *Pearson*'s correlation to examine the association between the occupancy rate of brain state and task-specific measures of cognitive control abilities. Individual data points were excluded in the analyses if they were 3 standard deviations or more away from the means.

I. Power analysis

We performed a power analysis based on the state-behavior relationship identified from our previous study [3]. If we set alpha at p=0.05, a sample size of 39 will provide power of 0.8 to detect the effect of interest.

II. Supplementary Results

Statistical significance of state matching

Space closeness was significant between SH_{WM} and SH_{AxCPT} (p=0.01) in the AxCPT, between SH_{WM} and SH_{CuedTS} (p=0.01) in the CuedTS, between SH_{WM} and SH_{Stern} (p=0.05) in the Sternberg and between SH_{WM} and SH_{Stroop} (p=0.01) in the Stroop. Space closeness was also significant between SH_{WM} and SH_{SST} (p=0.01) in the SST. Space closeness was marginally significant between SH_{WM} and SH_{RP1} (p=0.1) in RP Session 1 and significant between SH_{WM} and SH_{RP2} (p=0.04) in RP Session 2 (**Supplementary Table S13**).

Temporal closeness was significant between SH_{WM} and SH_{AxCPT} (p=0.01) in the AxCPT, between SH_{WM} and SH_{CuedTS} (p=0.01) in the CuedTS, between SH_{WM} and SH_{Stern} (p=0.01) in the Sternberg task and between SH_{WM} and SH_{Stroop} (p=0.01) in the Stroop. Temporal closeness was also significant between SH_{WM} and SH_{SST} (p=0.05) in the SST. Temporal closeness was significant between SH_{WM} and SH_{RP1} (p=0.01) in RP Session 1 and significant between SH_{WM} and SH_{RP2} (p=0.01) in the RP Session 2. (**Supplementary Table S13**).

Statistical significance of MFG role in leave one ROI out analysis

In the AxCPT, CuedTS and Stroop tasks, the left and right middle frontal gyrus (MFG) had a significant impact on state similarity ($p<0.05$, FDR corrected). In the in the Sternberg task, the left MFG had a significant impact on the state similarity ($p<0.05$, FDR corrected). In the SST, the left MFG had marginally significant impact on state similarity ($p=0.065$, FDR corrected). Taken together, across all the tasks, the MFG, encompassing the dorsolateral prefrontal cortex, had the highest impact on state similarity (**Supplementary Figure S5**), suggesting that this region is the most important and consistent brain region whose dynamic features contribute to a shared high-load brain state across cognitive tasks.

Robustness of state matching with respect to sample size of the N-back task

To examine the robustness of our findings with respect to the sample size used in generating the reference optimal latent brain state SH_{WM}, we leveraged a larger sample (415 subjects) from the HCP 1200 n-back working memory task and repeated the same BSDS analysis to identify the optimal latent brain state SH_{WM415}. Specifically, we applied similar data inclusion criteria as in our previous study with a more lenient behavioral criterion (accuracy > 50%) allowing more subjects to be included. The selection criteria were: (1) range of head motion in any translational and rotational direction less than 1 voxel; (2) average scan-to-scan head motion less than 0.25 mm; (3) performance accuracy per session >50%; (4) criterion (1)–(3) met in both Sessions 1 and 2 separately; (5) right handed subjects, and (6) subjects are unrelated. This leads to the final sample size of 415 subjects (29±4 years old, 22-36 years old, 225 F/190 M).

We applied the same data analysis procedures as in our previous sample. We determined the optimal latent brain state in the n-back working memory task by its dominant occupancy rate in the 2-back working memory blocks and its positive correlation with 2-back task accuracy (**Supplementary Figure S10**). This optimal latent brain state was labeled as SH_{WM415}, to differentiate the optimal latent brain (SH_{WM}) state reported previously [3].

Next, we used two state-matching algorithms to determine the correspondence between states in each cognitive task and *n*-back tasks. Noteworthy, here the key question is whether the state matched to the optimal brain state in the n-back working memory task (from HCP 415 subjects) is the same state matched to the optimal brain state in the n-back working memory task reported in our previous study, which is generated using HCP 122 subjects. To facilitate a straightforward comparison, the new brain states from the n-back working memory task (from HCP 415 subjects) were labeled as SX_{WM415}, in particular the optimal brain state was labeled as SH_{WM415}, and the brain states from all the other cognitive control tasks remained the same labels, which are matched to the SH_{WM}. We found that SH_{AxCPT}, SH_{CuedTS}, SH_{Stern}, SH_{Stroop}, SH_{SST}, SH_{RP1} and SH_{RP2} matches to SH_{WM415} in each corresponding cognitive task and results from two state-matching algorithm are converging (**Supplementary Figure S11-13**).

In summary, we replicated the key state matching results such that the state matched to SH_{WM415} is the same state matched to SH_{WM} in each cognitive task (**Supplementary Figures S11-S13**). Results further demonstrate the robustness and generalizability of our findings.

Robustness of state matching with respect to reference cognitive tasks

To examine the robustness of our findings with respect to the choice of reference cognitive task, we tested state matching using alternative cognitive tasks as reference.

First, we used the HCP RP task as the reference task. The optimal latent brain state in the Relational Processing task was determined by the occupancy rate of the latent brain state and

relation to its cognitive control efficiency score (Accuracy/RT) (**Supplementary Table S9**). This optimal brain state in the RP task was the same state that best match with SH_{WM}, i.e. SH_{RP}. Next, using SH_{RP} as the reference state, we examined whether the state that matched to SH_{RP} was the same state matched to SH_{WM}. To simplify the comparison with original results, the same state labels were used in these analyses. **Supplementary Figure S14** shows the state matched to SH_{RP} in each DMCC task. **Supplementary Figure S15** shows the state matched to SH_{RP} in the SST. It turns out that, in each cognitive task, the state matched to SH_{RP} was the same state that matched SH_{WM}.

Second, we repeated the same analysis using the SST as the reference task. The optimal latent brain state in the SST was determined by the occupancy rate of the latent brain state and correlation with 1/SSRT, the cognitive control performance index of the SST (**Supplementary Table S9**). The optimal brain state in the SST was the same state that best matched SH_{WM}, i.e. SH_{SST}. Next, using SH_{SST} as the reference state, we examined whether the state matched to SH_{SST} was the same state matched to SH_{WM}. To simplify the comparison with original results, the same state labels were used in these analyses. **Supplementary Figure S16** shows the state matched to SH_{SST} in each DMCC task. **Supplementary Figure S17** shows the state matched to SH_{SST} in the RP task. Again, it turns out that, in each cognitive task, the state matched to SH_{SST} was the same state that matched SH_{WM}.

In summary, we replicated the key state matching results such that the state matched to the optimal brain state in other reference cognitive tasks was the same state matched to the SH_{WM}. Results further demonstrate the robustness and generalizability of our findings.

Robustness of the main findings with respect to ROI selection

To examine the robustness of the main findings with respect to ROI selection, we conducted additional analysis using ROIs from an independent meta-analysis. We used NeuroSynth with key term “working memory”, which produces a meta-analytic brain map of 1091 studies (https://neurosynth.org/). We selected 9 ROIs from the meta-analysis, including bilateral anterior insula, middle frontal gyrus, frontal eye field, intraparietal lobule and right dorsomedial prefrontal cortex, which match the ROIs used in the main analysis. Also, because the meta-analysis did not include deactivated regions, posterior cingulate cortex and ventromedial prefrontal cortex of the DMN in the main analysis were included in the new ROI set (**Supplementary Figure S18a**) in order to keep the same number of ROIs (N=11) to match dimensionality and the same approximate cognitive systems. We then tested whether the optimal latent brain state in the n-back working memory task with ROI derived from the meta-analysis also plays behaviorally significant role in other cognitive tasks.

First, we applied BSDS to probe latent brain dynamics in the HCP n-back working memory task. Here the same sample of 122 subjects was used in the analysis. BSDS uncovered 4 distinct latent brain state (**Supplementary Figure S18b**). We determined the optimal latent brain state in the n-back working memory task by its dominant occupancy rate in the 2-back working memory blocks and its positive contribution to accuracy in the 2-back task condition (r=0.38, p<0.001, **Supplementary Figure S18d**). Here, the optimal latent brain state is labeled as SH_{META}, to differentiate the optimal latent brain (SH_{WM}) state reported in our previous study.

Next, we applied BSDS to probe latent brain dynamics in each of the four DMCC cognitive control tasks independently. BSDS uncovered 5 latent brain states in the AxCPT, 6 latent brain states in the CuedTS, 5 latent brain states in the Sternberg and 6 latent brain states in the

Stroop. Two state-matching algorithms were used to determine the state that matches SH_{META}, which is labeled as SH_{AxCPT}, SH_{CuedTS}, SH_{Stern}, SH_{Stroop} in each DMCC task, respectively (**Supplementary Figure S19**).

We then examined whether the latent brain state that matches SH_{META} plays an important role in cognitive control in each DMCC task. We conducted the same brain-behavior analyses, including multivariate CCA and univariate *Pearson's* correlation analysis. CCA revealed significant canonical correlations between occupancy rate (OR) of latent brain states and key behavioral measures in each DMCC task (**Supplementary Figure S20a-d**). Univariate analysis revealed that OR of SH_{AxCPT} is significantly and positively correlated with cognitive control index in the AxCPT (r=0.29, p=0.04); OR of SH_{CuedTS} is positively correlated with cognitive control index in the CuedTS with marginal significance (r=0.25, p=0.08); OR of SH_{Stern} is significantly and positively correlated with cognitive control index in the Sternberg (r=0.37, p=0.007); and OR of SH_{Stroop} is significantly and positively correlated with cognitive control index in the Stroop (r=0.28, p=0.04) (**Supplementary Figure S20e-h**).

Next, we applied BSDS to probe latent brain dynamics in the SST independently. BSDS uncovered 4 latent brain states in the SST. Two state-matching algorithms were used to determine the state that matches SH_{META}, which is labeled as SH_{SST} (**Supplementary Figure S21**).

We then examined whether the latent brain state that matches SH_{META} plays an important role in the SST. We conducted the same brain-behavior analyses, including multivariate CCA and univariate *Pearson's* correlation analysis. CCA revealed significant canonical correlations between OR of latent brain states and key behavioral measures in the SST (**Supplementary Figure S22**). Univariate analysis revealed that OR of SH_{SST} is significantly and positively correlated with cognitive control index in the SST (r=0.32, p=0.03) (**Supplementary Figure S22**).

Finally, we applied BSDS on the RP task. In both sessions of the RP task, BSDS uncovered 4 latent brain states. Two state-matching algorithms were used to determine the states that match SH_{META}, which are labeled as SH_{RP1} and SH_{RP2} in session 1 and 2, respectively (**Supplementary Figure S23**).

We examined whether the latent brain state that matches SH_{META} plays an important role in each session of the RP task. We conducted the same brain-behavior analyses, including multivariate CCA and univariate *Pearson's* correlation analysis. CCA revealed significant canonical correlations between OR of latent brain states and key behavioral measures in each session of the RP task (**Supplementary Figure S24a**). Univariate analysis revealed that OR of SH_{RP1} is significantly and positively correlated with cognitive control index in the session 1 (r=0.25, p=0.02) and OR of SH_{RP2} is significantly and positively correlated with cognitive control index in the session 2 (r=0.28, p=0.01) (**Supplementary Figure S24b**).

In summary, we replicated the key results in the main analysis using ROIs derived from the meta-analysis, demonstrating the robustness of our findings with respect to the selection of ROIs.

III. Supplementary Figures

Supplementary Figure S1. Schematic overview of AxCPT, CuedTS, Sternberg, Stroop, SST and RP tasks.

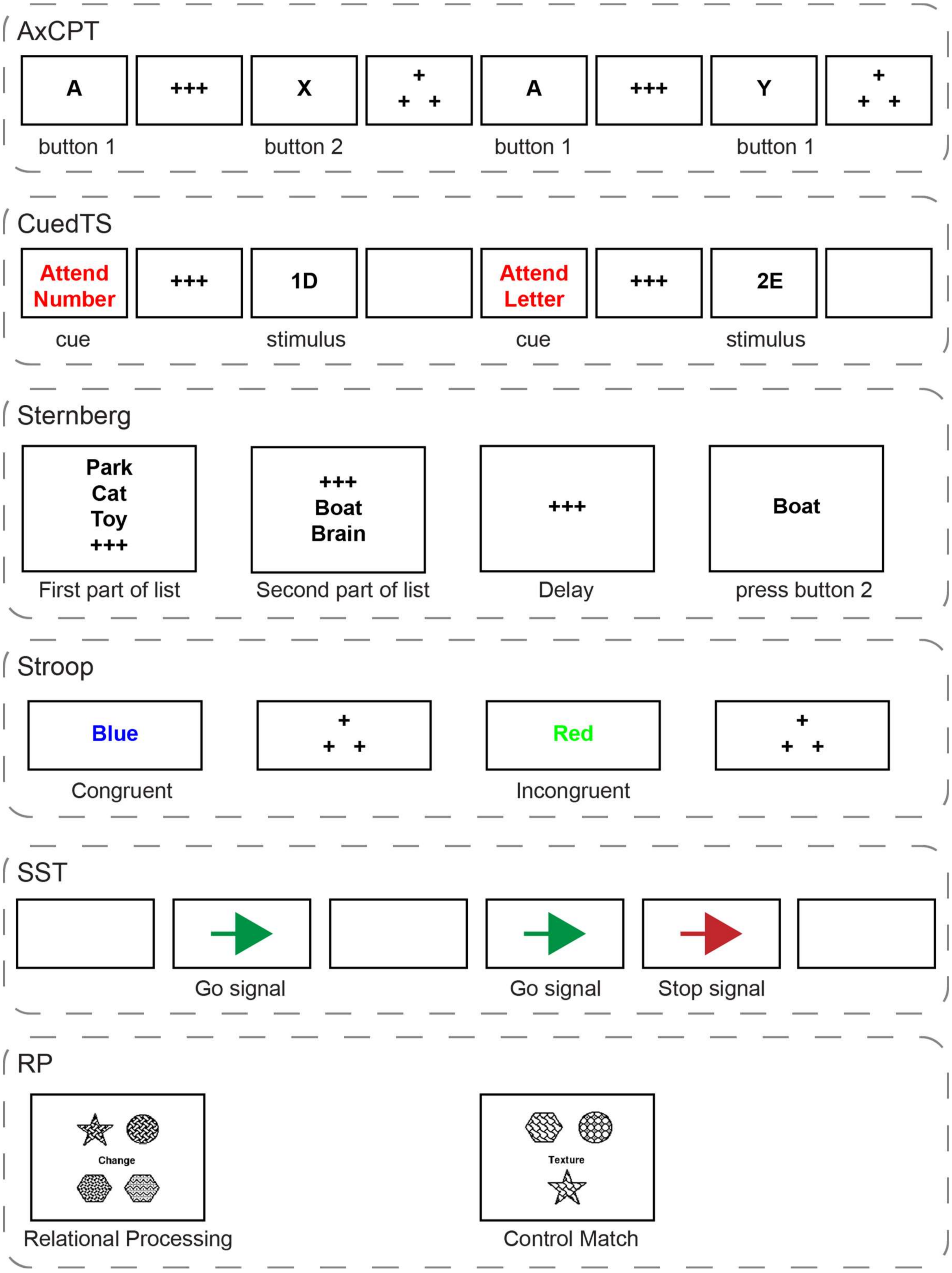

Supplementary Figure S2. Brain network model with nodes in the Salience Network (SN), Frontal-Parietal Network (FPN) and Default Mode network (DMN) (a): 1, left anterior insula (lAI); 2, right anterior insula (rAI); 3, dorsomedial prefrontal cortex (DMPFC); 4, left middle frontal gyrus (lMFG); 5, right middle frontal gyrus (rMFG); 6, left frontal eye field (lFEF); 7, right frontal eye field (rFEF); 8, left intraparietal lobule (lIPL); 9, right intraparietal lobule (rIPL); 10, posterior cingulate cortex (PCC) and 11, ventromedial prefrontal cortex (VMPFC). Multivariate features associated with SH_{WM}, the task-optimal reference *n*-back working memory task. **(b)** Mean and **(c)** Covariance. Source data are provided as a Source data file.

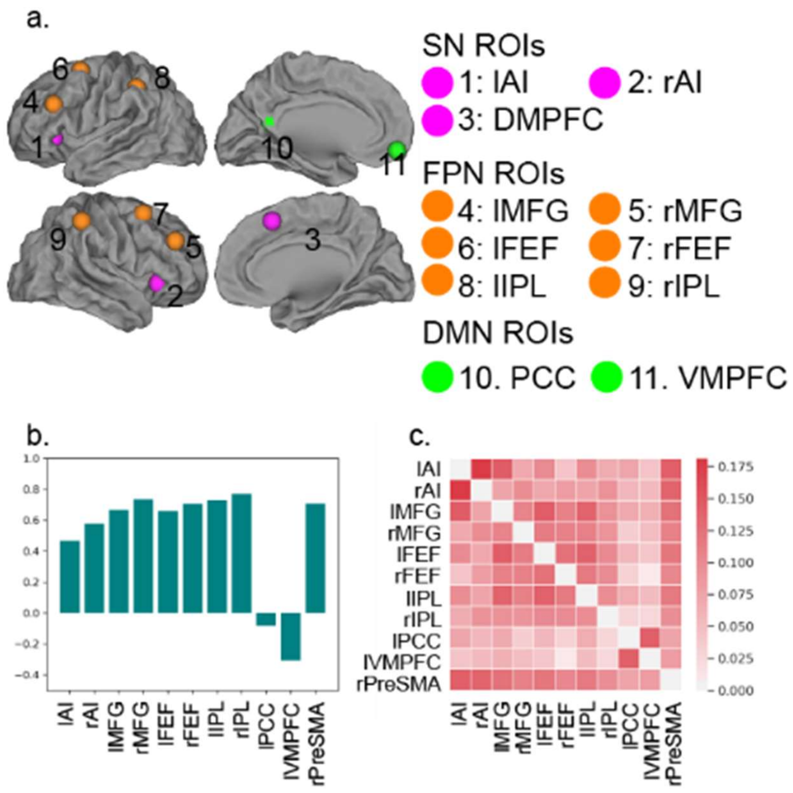

Supplementary Figure S3. Brain states in each of the four dual models of cognitive control (DMCC) tasks and their relation to the reference state SH_{WM}. Each state is characterized by a unique activation/deactivation and connectivity pattern (N=50). (**a**) In the AxCPT, SH_{AxCPT} is the brain state that matches SH_{WM}; the other states $S1_{AxCPT}$, $S10_{AxCPT}$, $S7_{AxCPT}$, $S5_{AxCPT}$, $S8_{AxCPT}$ do not match SH_{WM}. (**b**) In the CuedTS, SH_{CuedTS} is the brain state that matches SH_{WM}; the other states $S7_{CuedTS}$, $S9_{CuedTS}$, $S3_{CuedTS}$, $S10_{CuedTS}$, and $S5_{CuedTS}$ do not match SH_{WM}. (**c**) In the Sternberg, SH_{Stern} is the brain state that matches SH_{WM} whereas $S5_{Stern}$, $S4_{Stern}$, and $S2_{Stern}$ are the other states. (**d**) In the Stroop, SH_{Stroop} is the brain state that matches SH_{WM}; the other states $S6_{Stroop}$, $S3_{Stroop}$, $S5_{Stroop}$, and $S1_{Stroop}$ do not match SH_{WM}. Source data are provided as a Source data file.

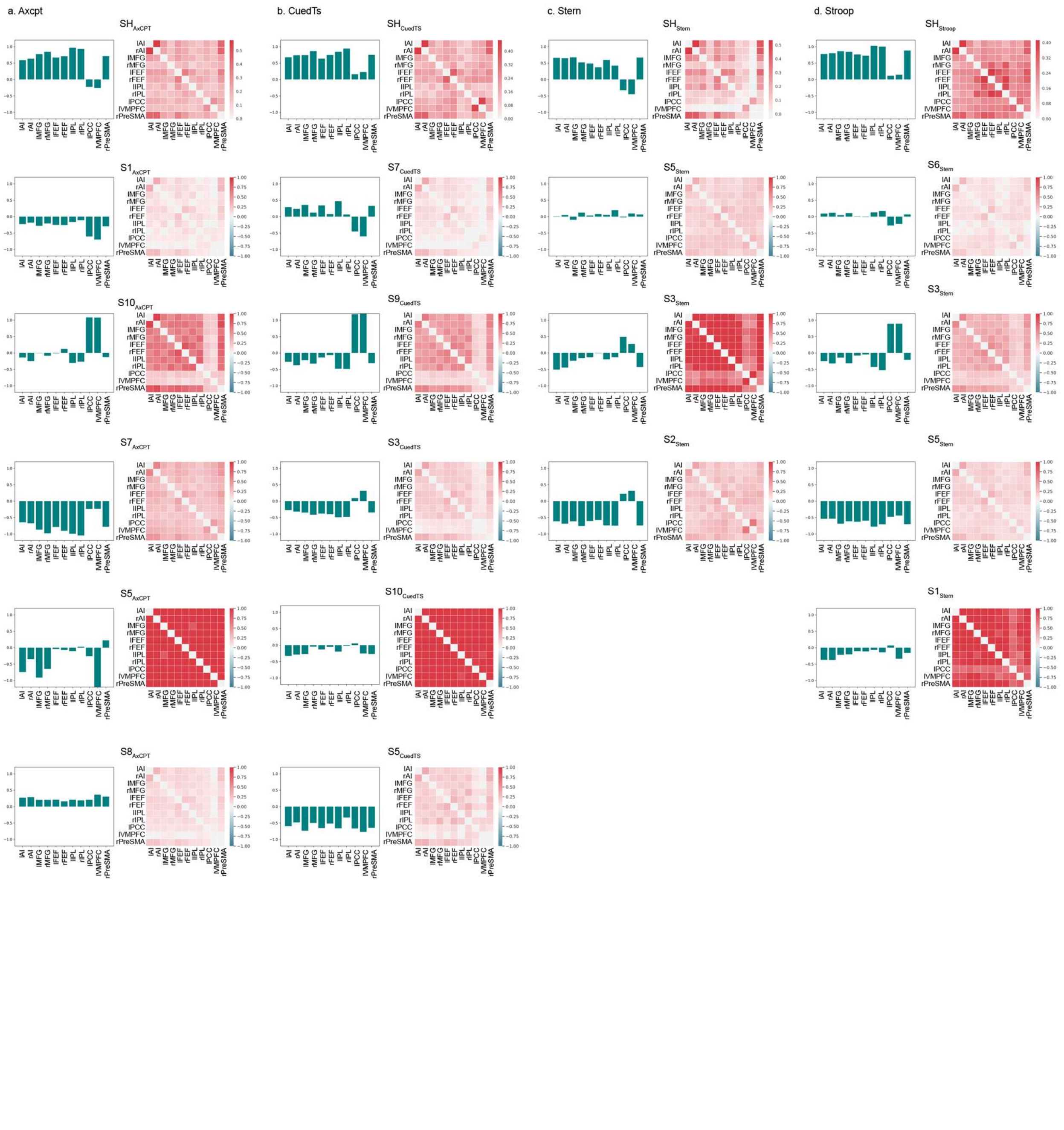

Supplementary Figure S4. Brain states in SST and their relation to the reference state SH_{WM} (N=45). Each state is characterized by a unique activation/deactivation and connectivity patterns. SH_{SST} is the brain state that matches SH_{WM}; the other states $S10_{SST}$, $S3_{SST}$, and $S8_{SST}$ do not match SH_{WM}. Source data are provided as a Source data file.

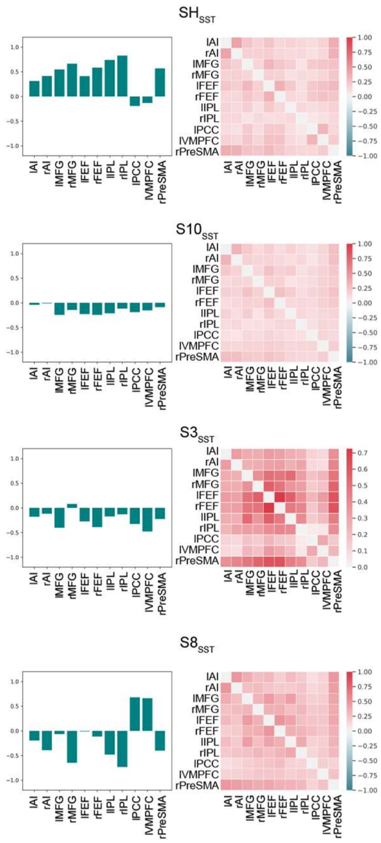

Supplementary Figure S5. **Leave one ROI out analysis reveals critical role of middle frontal gyrus (MFG).** The MFG was the single most important brain region whose dynamic features contribute to shared latent brain states across cognitive tasks. (**a**) SH_{AxCPT}, (**b**) SH_{CuedTS}, (**c**) SH_{Stern}, (**d**) SH_{Stroop}, and (**d**) SH_{SST}. Source data are provided as a Source data file.

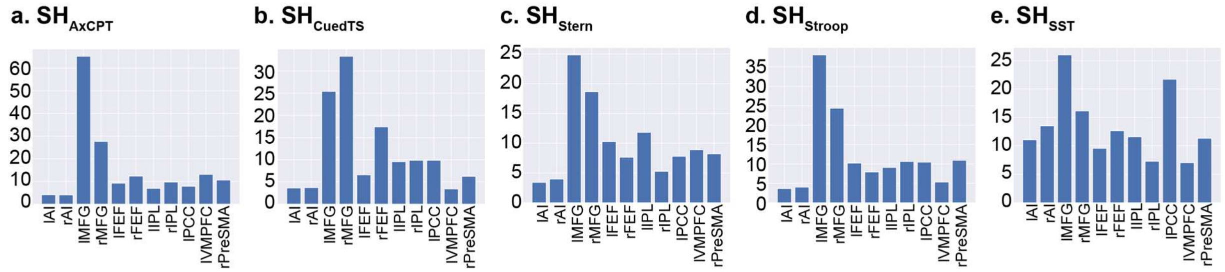

Supplementary Figure S6. Brain states in the RP task and their relation to the reference state SH_{WM} (N=90). Each state is characterized by a unique activation/deactivation and connectivity patterns. (**a**) In Session 1, SH_{RP1} is the brain state that matches SH_{WM}, $S15_{RP1}$, $S12_{RP1}$, and $S6_{RP1}$ are the other states. (**b**) In Session 2, SH_{RP2} is the brain state that matches SH_{WM}; the other states $S11_{RP2}$, $S6_{RP2}$, and $S9_{RP2}$ do not match SH_{WM}. Source data are provided as a Source data file.

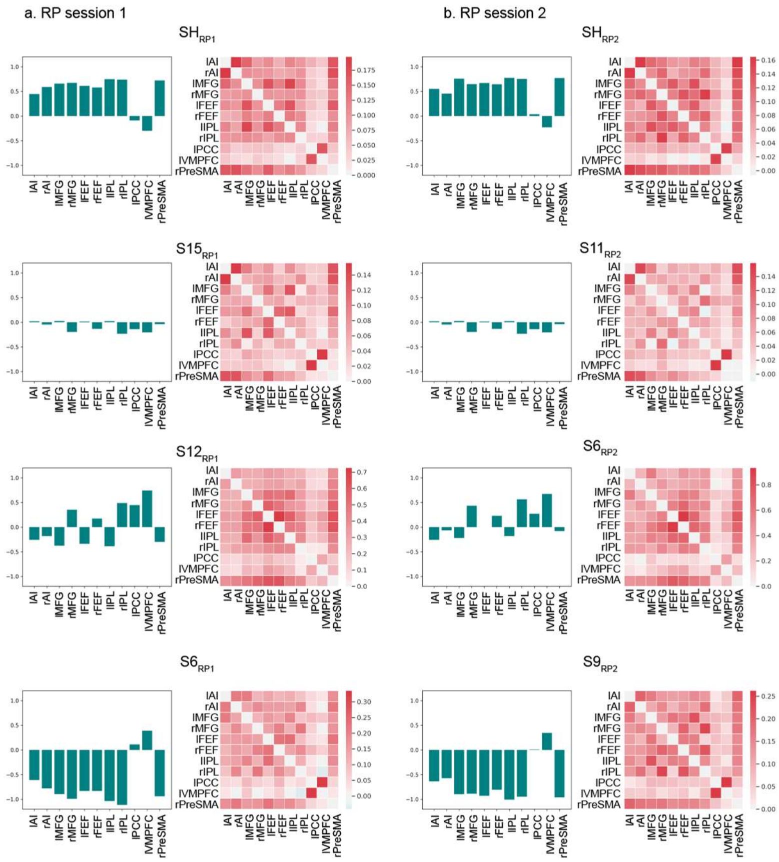

Supplementary Figure S7. **Shared latent brain states within individuals across sessions during the relation processing task. (a)** Individuals (N=90) who have the higher occupancy rates of the SH_{WM} during the n-back working memory task also have the higher occupancy rates SH_{RP1} in Session 1 (r=0.43, p<0.001, *Pearson's* correlation). **(b)** Findings were replicated in Session 2 with SH_{RP2} (r=0.24, p=0.02, *Pearson's* correlation). The regression estimate is presented with 95% confidence interval (shaded area). P values were not adjusted for multiple comparisons. Source data are provided as a Source data file.

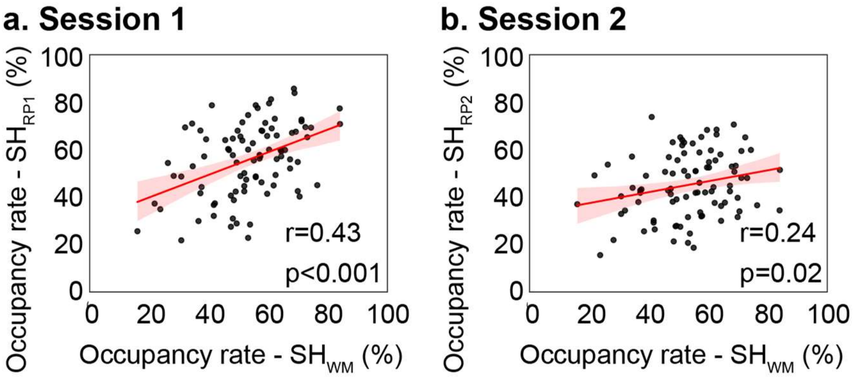

Supplementary Figure S8. Activation and connectivity patterns in each of the four dual models of cognitive control (DMCC) tasks (N=50). Contrasts of interest include (**a**) 2back *minus* 0-back in the *n*-back working memory task; (**b**) AY *minus* AX in the AxCPT task; (**c**) Incongruent *minus* Congruent in the CuedTS task; (**d**) High load (HL) *minus* Low load (LL) in the Sternberg task; (**e**) Incongruent *minus* Congruent conditions in the Stroop; (f) SuccStop *minus* Go in the SST task, (**g**) Relation Processing *minus* Match Control in the RP task sessions 1 and 2. Data are presented as mean values +/- SEM. Source data are provided as a Source data file.

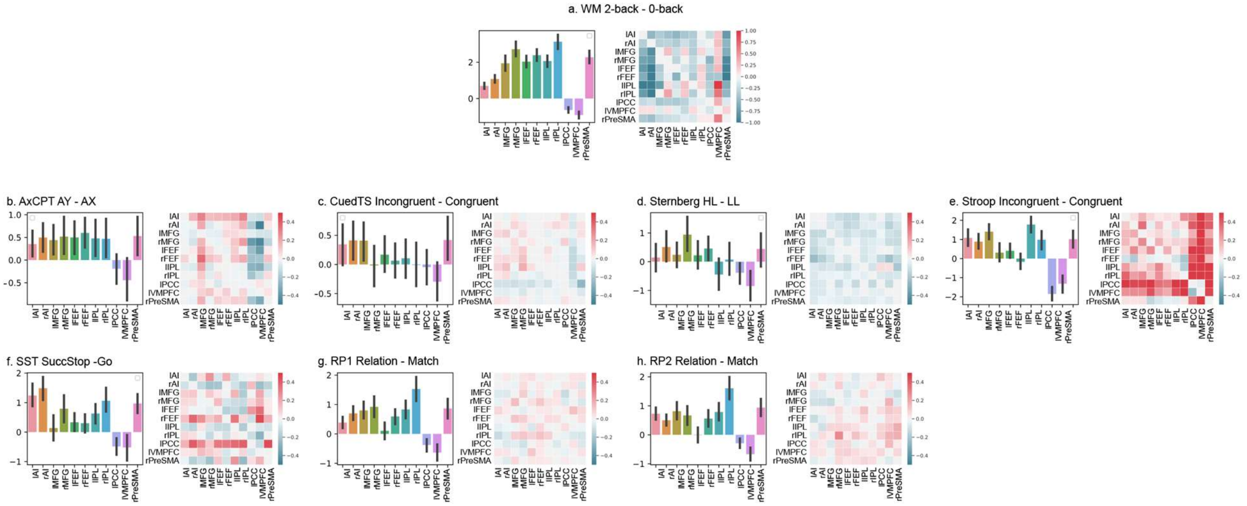

Supplementary Figure S9. (**a**) Similarity of activation/deactivation measures between the reference *n*-back working memory task and each of the other seven cognitive tasks using BSDS state-space models (blue) and conventional GLM (orange). **(b)** Similarity of connectivity measures between the reference *n*-back working memory task and each of the other seven cognitive tasks using BSDS state-space models (blue) and conventional GLM (orange). Across all seven cognitive tasks BSDS-derived models generated better model fits with the reference *n*-back working memory task than conventional GLM and gPPI analyses. AxCPT: AY-AX; CuedTS: Incongruent-Congruent; Stern: HL-LL; Stroop: Incongruent-Congruent; SST: SuccStop-Go; RP1: Relation Processing session 1; RP2: Relation Processing session 2. Source data are provided as a Source data file.

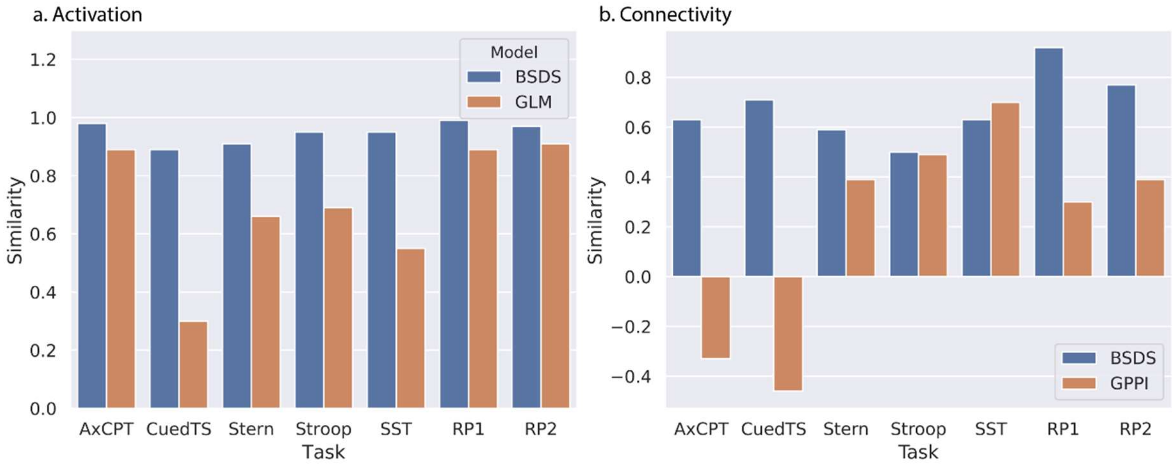

Supplementary Figure S10. **Latent brain states uncovered from the HCP n-back working memory task with 415 subjects. (a)** Temporal evolution of the five latent brain state identified in each of the 415 subjects. **(b)** Corresponding task waveforms of the three task conditions in the n-back WM task-0-back, 2-back and fixation blocks-are shown in the same layout. **(C)** OR of the optimal latent brain state for the 2-back condition (SH_{WM415}) was significantly correlated with performance accuracy in the 2-back condition (r=0.27, *p*<0.001, *Pearson's* correlation). The regression estimate is presented with 95% confidence interval (shaded area). Source data are provided as a Source data file.

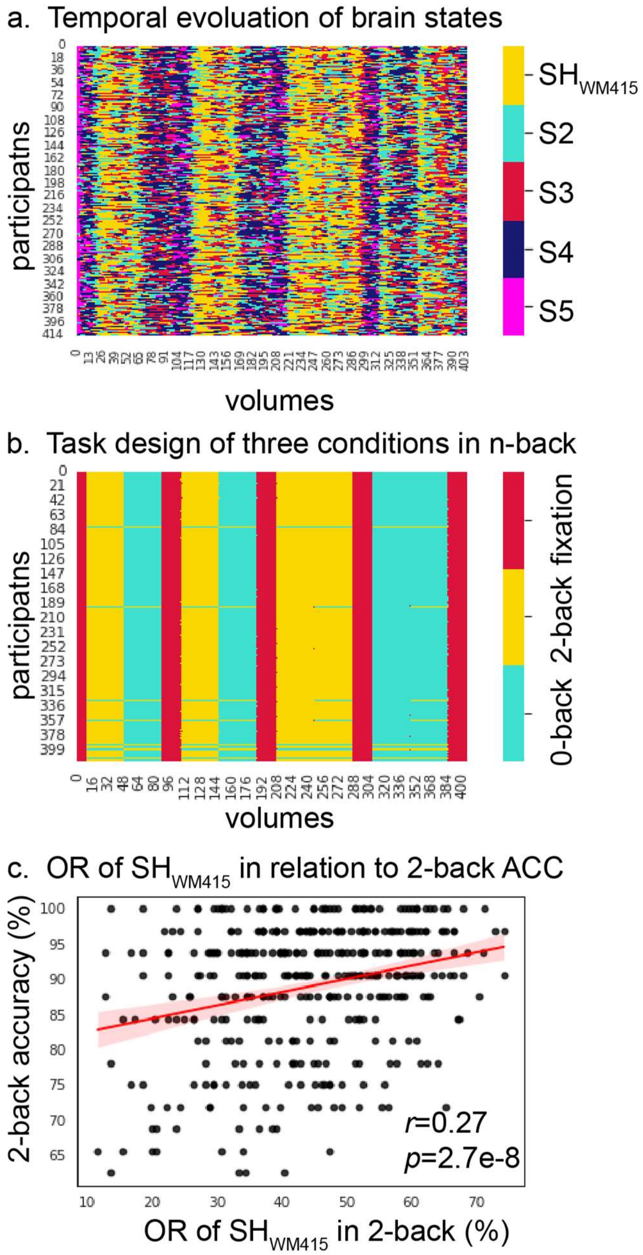

Supplementary Figure S11. **Shared latent brain state across four different dual mode of cognitive control (DMCC) tasks using reference SH_{WM415} reference states. (a,b)** BSDS uncovered 6 dynamic brain states in the AxCPT (N=50). SH_{AxCPT} showed the highest state space closeness (c=1.2) and highest state temporal closeness (r=0.8) with SH_{WM415}. BSDS uncovered 6 dynamic brain states in the CuedTS task. SH_{CuedTS} showed the highest state space closeness (c=1.1) and the highest state temporal closeness (r=0.8) with SH_{WM415}. BSDS uncovered 4 dynamic brain states in the Sternberg working memory task. SH_{Stern} showed the highest state space closeness (c=0.78) and highest state temporal closeness (r=0.62) with SH_{WM415}. BSDS uncovered 5 dynamic brain state in the Stroop task. SH_{Stroop} showed the highest state space closeness (c=1.2) and the highest state temporal closeness (r=0.9) with SH_{WM415}. SH_{WM415} refers to the high-load dynamic brain state in the n-back working memory task from a HCP cohort of 415 subjects. In each task, four best matched latent states are illustrated here. Color bars are the scales for state space closeness and state temporal closeness.

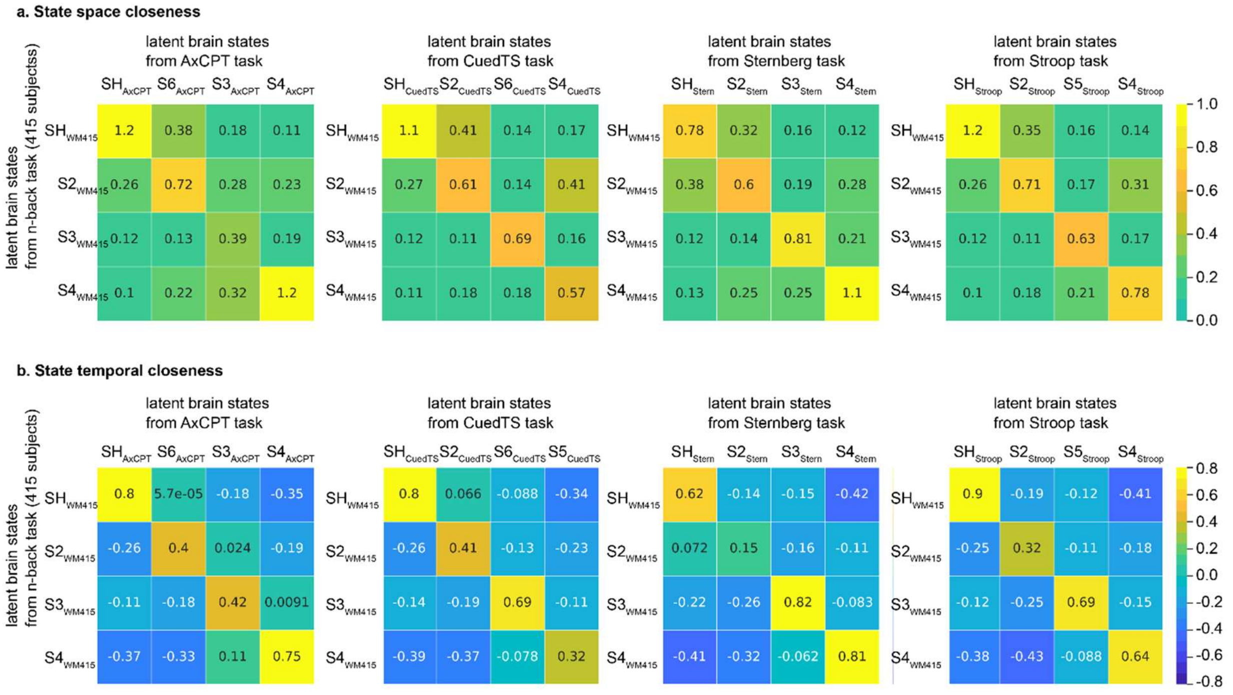

Supplementary Figure S12. **Shared latent brain states in the SST using the SH_{WM415} reference state. (a)** BSDS uncovered 4 dynamic brain states (N=45). SH_{SST} showed the highest state space closeness (r=1.7). **(b)** SH_{SST} also showed the highest state temporal closeness (c=0.75) with SH_{WM415}. SH_{WM415} refers to the high-load dynamic brain state in the n-back working memory task from a HCP 1200 cohort of 415 subjects. Color bars are the scales for state space closeness and state temporal closeness.

a. State space closeness

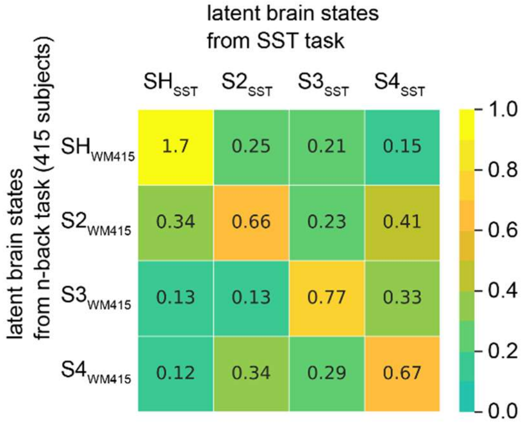

b. State temporal closeness

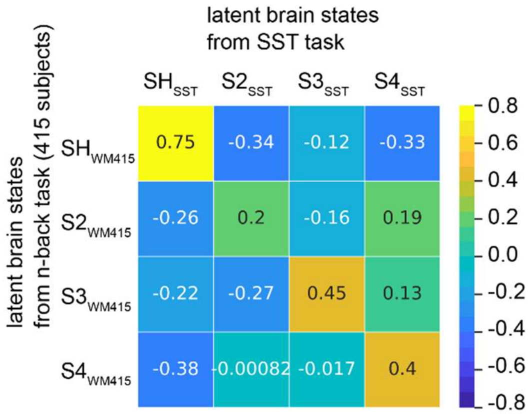

Supplementary Figure S13. **Shared latent brain states in the RP task using SH_{WM415} reference states. (a, b)** BSDS uncovered 4 dynamic brain states in both Sessions 1 and 2 (N=90). SH_{RP1} has the highest state space closeness (c=5) and the highest state temporal closeness (r=0.92) with SH_{WM415} in Session 1. SH_{RP2} has the highest state space closeness (c=5.3) and the highest state temporal closeness (r=0.94) with SH_{RP} in Session 2. SH_{WM415} refers to the high-load dynamic brain state in the n-back working memory task from a HCP 1200 cohort of 415 subjects. Color bars are the scales for state space closeness and state temporal closeness.

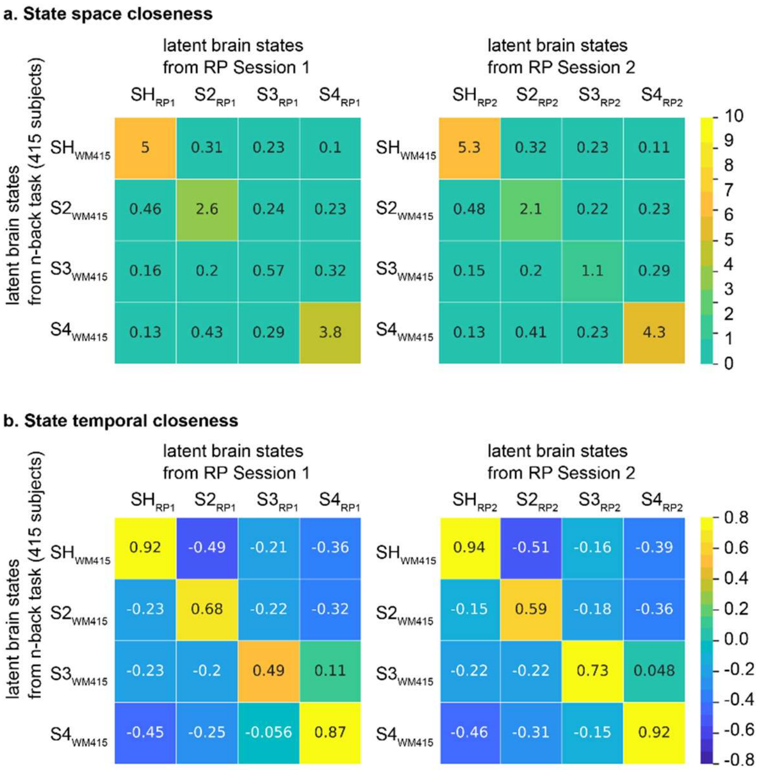

Supplementary Figure S14. **Shared latent brain state across four different dual mode of cognitive control (DMCC) tasks using reference states SH_{RP} from the RP task. (a,b)** BSDS uncovered 6 dynamic brain states in the AxCPT (N=50). SH_{AxCPT} showed the highest state space closeness (c=1.2) and highest state temporal closeness (r=0.82) with SH_{RP}. BSDS uncovered 6 dynamic brain states in the CuedTS task. SH_{CuedTS} showed the highest state space closeness (c=0.96) and the highest state temporal closeness (r=0.79) with SH_{RP}. BSDS uncovered 4 dynamic brain states in the Sternberg working memory task. SH_{Stern} showed the highest state space closeness (c=0.88) and highest state temporal closeness (r=0.66) with SH_{RP}. BSDS uncovered 5 dynamic brain state in the Stroop task. SH_{Stroop} showed the highest state space closeness (c=1.1) and the highest state temporal closeness (r=0.85) with SH_{RP}. SH_{RP} refers to the high-load dynamic brain state in the RP task. In each task, the best matched four latent states are illustrated here. Color bars are the scales for state space closeness and state temporal closeness.

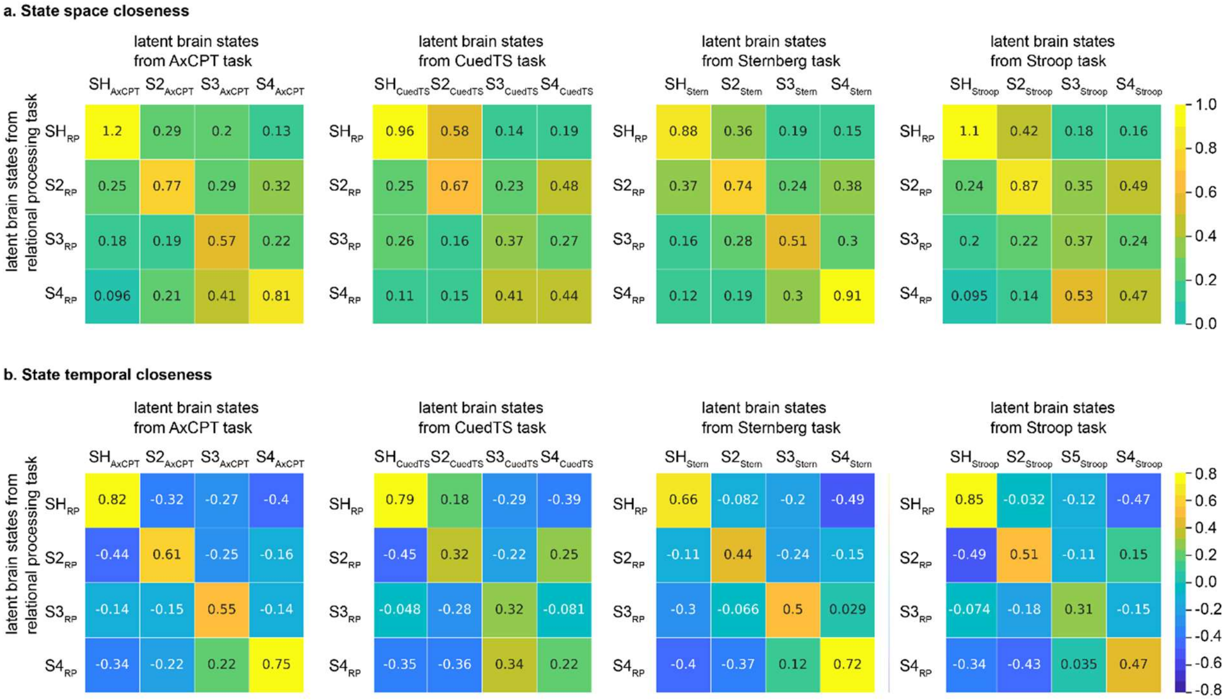

Supplementary Figure S15. **Shared latent brain states in the SST using reference states SH_{RP} from the RP task. (a)** BSDS uncovered 4 dynamic brain states (N=45). SH_{SST} showed the highest state space closeness (r=1.6). **(b)** SH_{SST} also showed the highest state temporal closeness (c=0.79) with SH_{RP}. SH_{RP} refers to the high-load dynamic brain state in the RP task. Color bars are the scales for state space closeness and state temporal closeness.

a. State space closeness

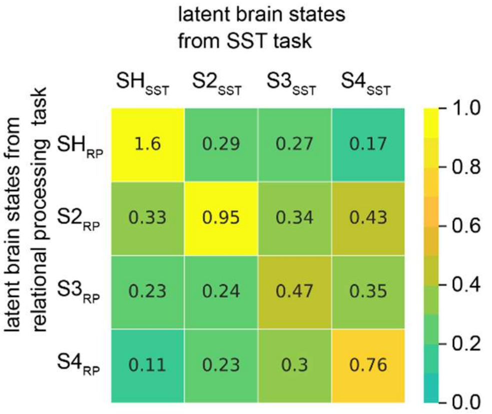

b. State temporal closeness

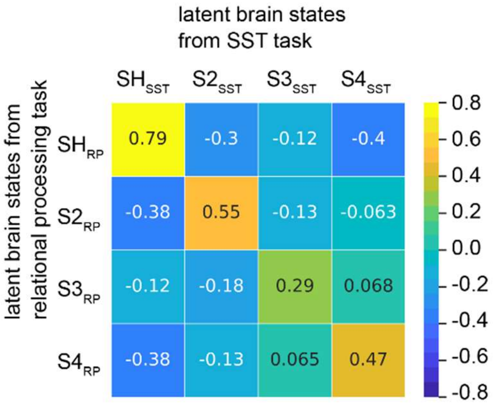

Supplementary Figure S16. **Shared latent brain state across four different dual mode of cognitive control (DMCC) tasks using reference states SH_{SST} from the SST. (a,b)** BSDS uncovered 6 dynamic brain states in the AxCPT (N=50). SH_{AxCPT} showed the highest state space closeness (c=2.3) and highest state temporal closeness (r=0.8) with SH_{SST}. BSDS uncovered 6 dynamic brain states in the CuedTS task. SH_{CuedTS} showed the highest state space closeness (c=1.6) and the highest state temporal closeness (r=0.7) with SH_{SST}. BSDS uncovered 4 dynamic brain states in the Sternberg working memory task. SH_{Stern} showed the highest state space closeness (c=1.1) and highest state temporal closeness (r=0.65) with SH_{SST}. BSDS uncovered 5 dynamic brain state in the Stroop task. SH_{Stroop} showed the highest state space closeness (c=2) and the highest state temporal closeness (r=0.81) with SH_{SST}. SH_{SST} refers to the high-load dynamic brain state in the stop-signal task. In each task, the best matched four latent states are illustrated here. Color bars are the scales for state space closeness and state temporal closeness.

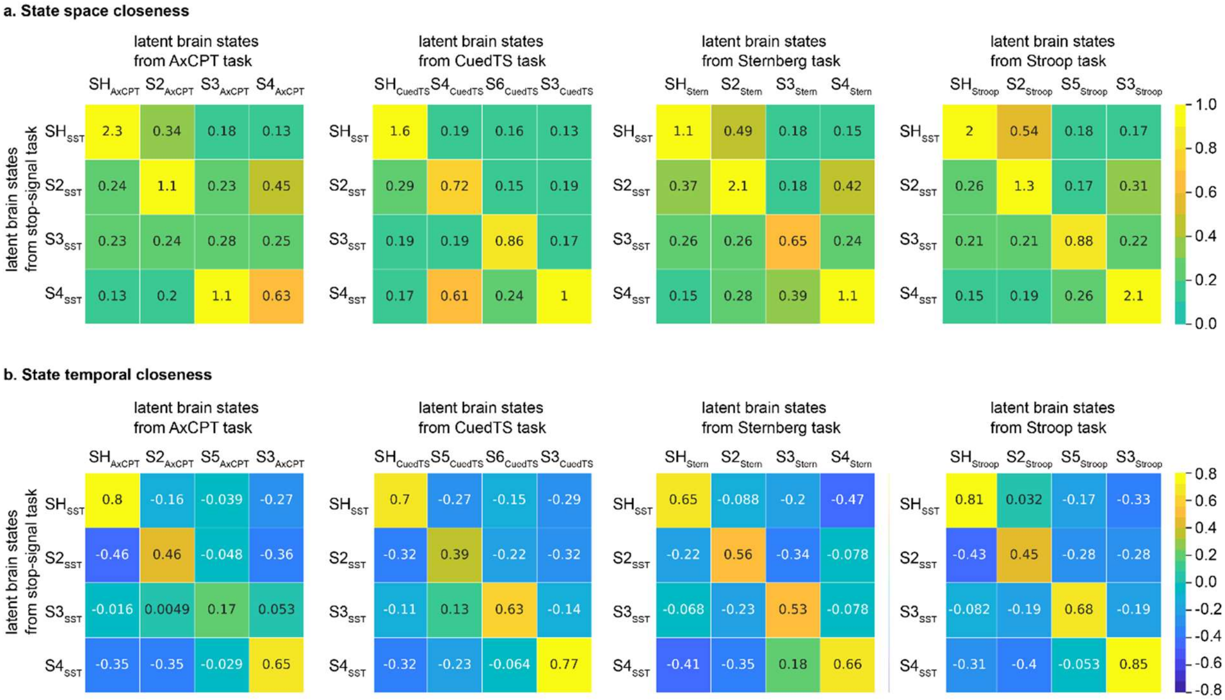

Supplementary Figure S17. **Shared latent brain states in the RP task using reference states SH_{SST} from the SST. (a, b)** BSDS uncovered 4 dynamic brain states in both Sessions 1 and 2 (N=90). SH_{RP1} has the highest state space closeness (c=1.6) and the highest state temporal closeness (r=0.84) with SH_{SST} in Session 1. SH_{RP2} has the highest state space closeness (c=1.3) and the highest state temporal closeness (r=0.82) with SH_{RP} in Session 2. SH_{SST} refers to the high-load dynamic brain state in the SST. Color bars are the scales for state space closeness and state temporal closeness.

a. State space closeness

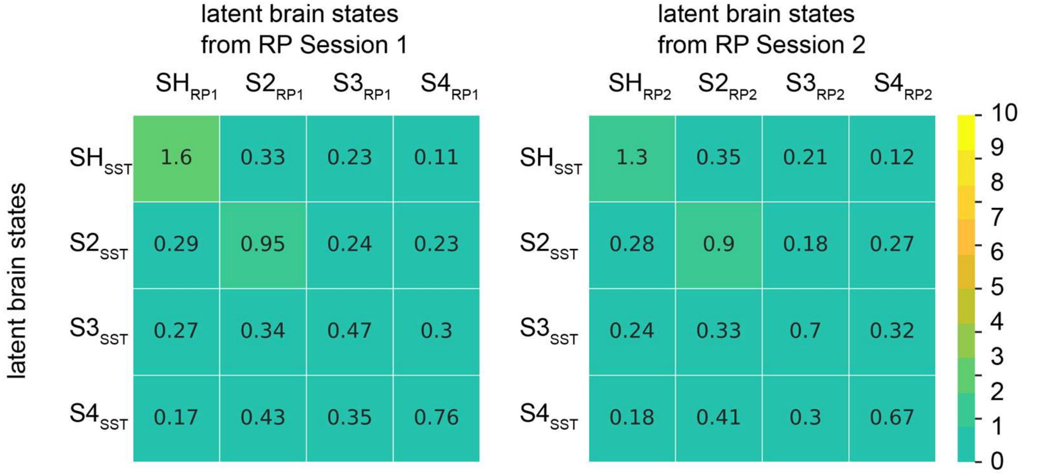

b. State temporal closeness

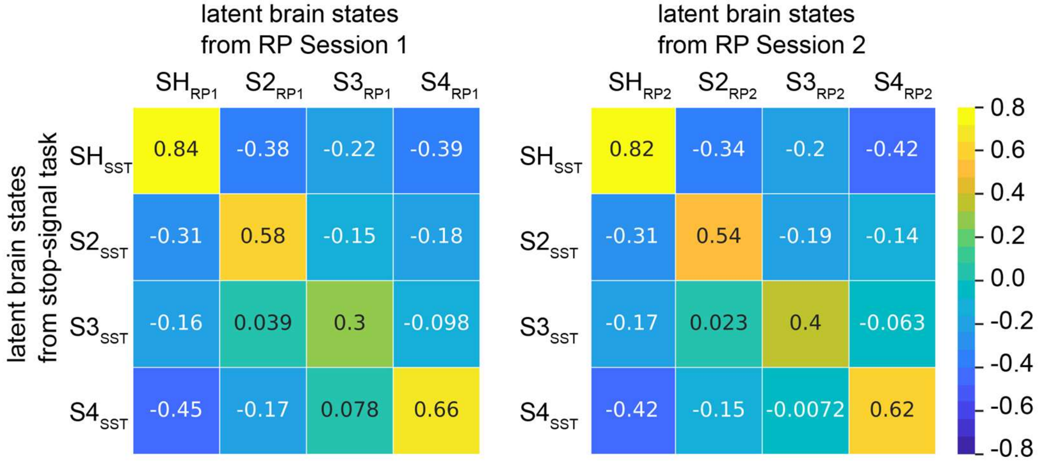

Supplementary Figure S18. **Latent brain states uncovered from the HCP n-back working memory task with ROIs from an independent meta-analysis. (a)** ROIs derived from meta-analysis of working memory in NeuroSynth**.** 1, left anterior insula (lAI); 2, right anterior insula (rAI); 3, dorsomedial prefrontal cortex (DMPFC); 4, left middle frontal gyrus (lMFG); 5, right middle frontal gyrus (rMFG); 6, left frontal eye field (lFEF); 7, right frontal eye field (rFEF); 8, left intraparietal lobule (lIPL); 9, right intraparietal lobule (rIPL); 10, posterior cingulate cortex (PCC) and 11, ventromedial prefrontal cortex (VMPFC). SN: salience network; FPN: frontoparietal network; DMN: default mode network. **(b)** Temporal evolution of the 4 latent brain state identified in each of the 122 subjects. **(c)** Corresponding task waveforms of the three task conditions in the n-back WM task-0-back, 2-back and fixation blocks-are shown in the same layout. **(d)** OR of the optimal latent brain state for the 2-back condition (SH_{META}) was significantly correlated with performance accuracy in the 2-back condition (r=0.27, p<0.001, *Pearson's* correlation). The regression estimate is presented with 95% confidence interval (shaded area). Source data are provided as a Source data file.

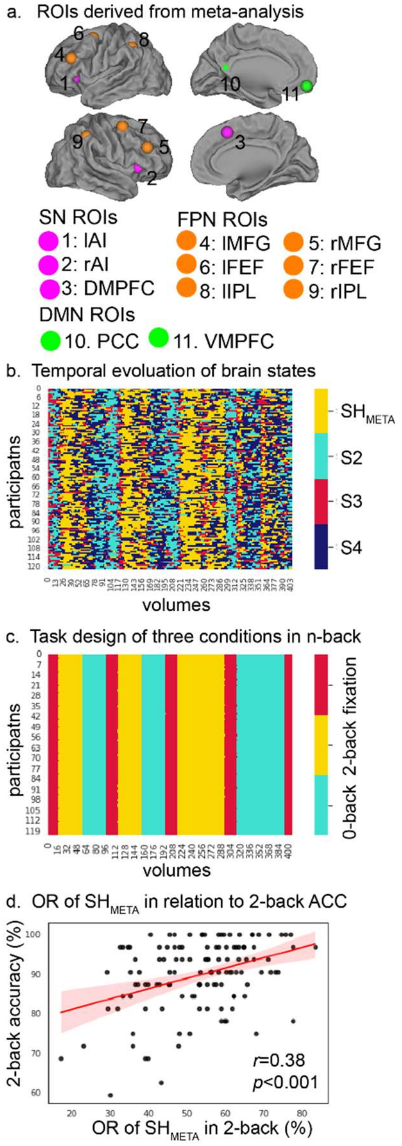

Supplementary Figure S19. **Shared latent brain state across four different dual mode of cognitive control (DMCC) tasks (reference is SH_{META}) with ROIs derived from an independent meta-analysis. (a,b)** BSDS uncovered 5 dynamic brain states in the AxCPT (N=50). SH_{AxCPT} showed the highest state space closeness (c=1) and highest state temporal closeness (r=0.82) with SH_{META}. BSDS uncovered 6 dynamic brain states in the CuedTS task. SH_{CuedTS} showed the moderate state space closeness (c=0.75) and the moderate state temporal closeness (r=0.67) with SH_{META}. BSDS uncovered 5 dynamic brain states in the Sternberg working memory task. SH_{Stern} showed the highest state space closeness (c=0.96) and the highest state temporal closeness (r=0.6) with SH_{META}. BSDS uncovered 6 dynamic brain state in the Stroop task. SH_{Stroop} showed the highest state space closeness (c=0.76) and the highest state temporal closeness (r=0.68) with SH_{META}. SH_{META} refers to the high-load dynamic brain state in the n-back working memory task with ROIs derived from an independent meta-analysis. In each task, the best matched four latent states are illustrated here. Color bars are the scales for state space closeness and state temporal closeness.

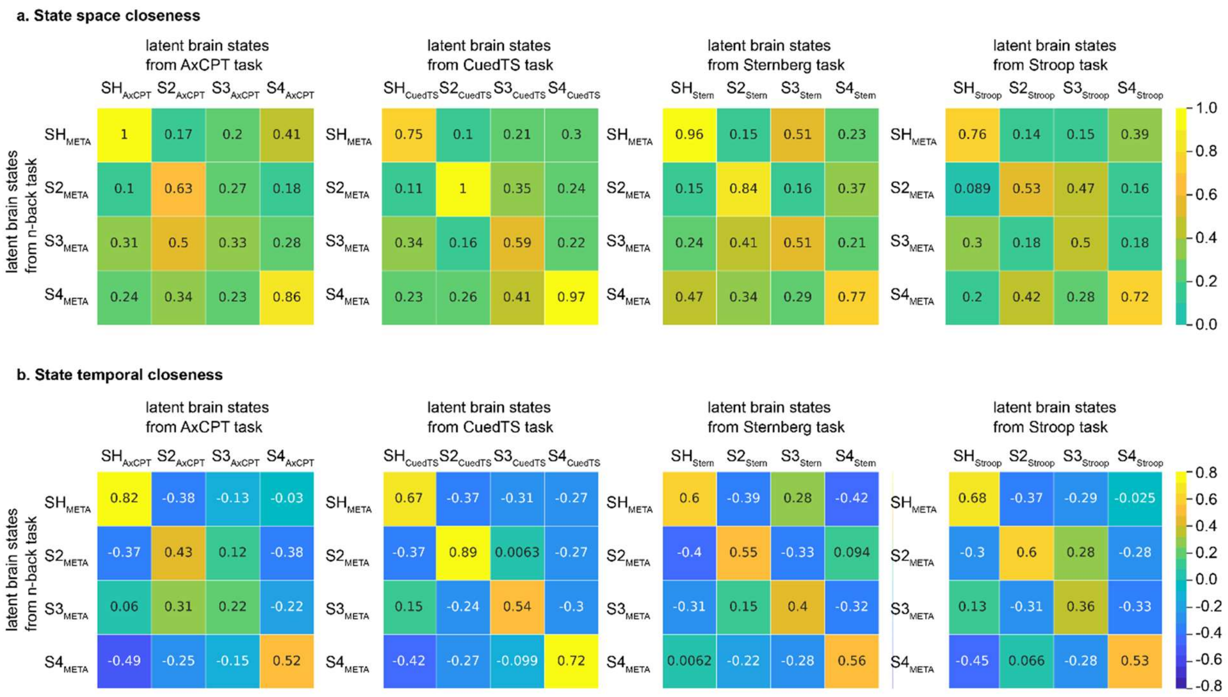

Supplementary Figure S20. **Latent brain states are associated with task performance in all four dual mode of cognitive control (DMCC) tasks with ROIs derived from an independent meta-analysis.** Multivariate CCA revealed significant correlations between occupancy rates of latent brain states and behavioral variables in all the DMCC tasks, including (**a**) AxCPT, (**b**) CuedTS, (**c**) Sternberg and (**d**) Stroop (N=50). In each task, the component in which linear combination of behavioral variables that best represents general cognitive control was selected to investigate the relationship between latent brain state and behavioral performance. Univariate *Pearson*'s correlation revealed significant correlation between occupancy rate of the multi-demand brain state (e.g. SH_{AxCPT}) and cognitive control index in all the DMCC tasks, including (**e**) AxCPT, (**f**) CuedTS, (**g**) Sternberg task, and (**h**) Stroop. SH_{AxCPT} , SH_{CuedTS} , SH_{Stern} and SH_{Stroop} refers to the dynamic brain state that matches to SH_{WM} in the AxCPT, CuedTS, Sternberg and Stroop tasks, respectively. AxCPT = Ax Continuous performance task; CuedTS = Cued task switching task; ACC = Accuracy; RT = Reaction time; LL = Low load; HL = High load; Con = Congruent; Incon = Incongruent; OR = Occupancy rate. The regression estimate is presented with 95% confidence interval (shaded area). P values were not adjusted for multiple comparisons. Source data are provided as a Source data file.

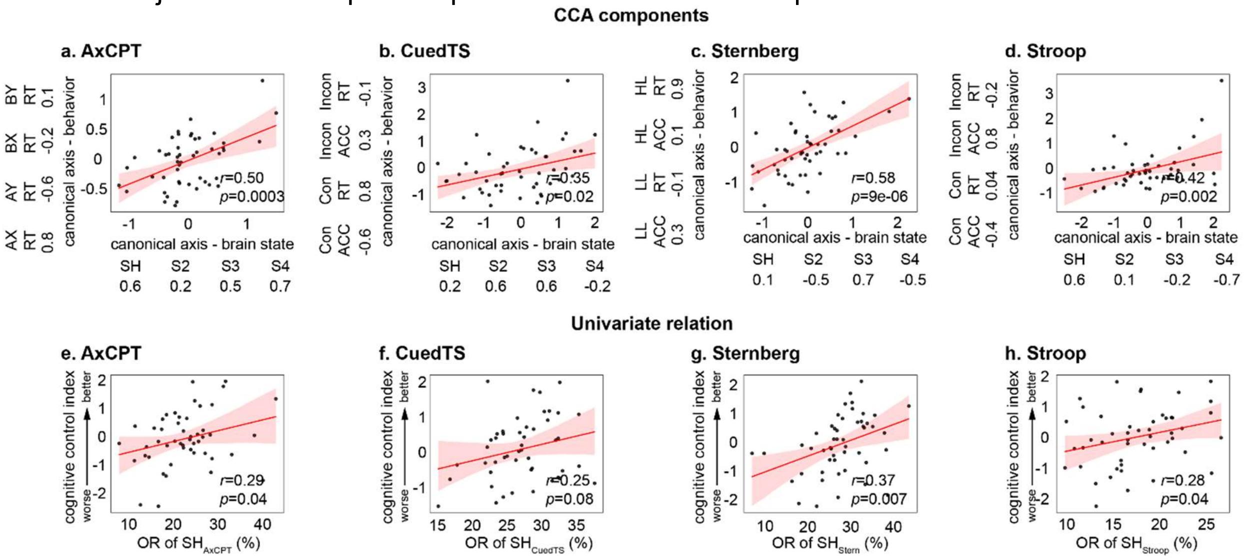

Supplementary Figure S21. **Shared latent brain state in the SST (reference is SH_{META}) with ROIs derived from an independent meta-analysis.** BSDS uncovered 4 dynamic brain states in the SST (N=45). SH_{SST} showed **(a)** the highest state space closeness (c=1.2) and **(b)** highest state temporal closeness (r=0.78) with SH_{META}. SH_{META} refers to the high-load dynamic brain state in the n-back working memory task with ROIs derived from an independent meta-analysis. Color bars are the scales for state space closeness and state temporal closeness.

a. State space closeness

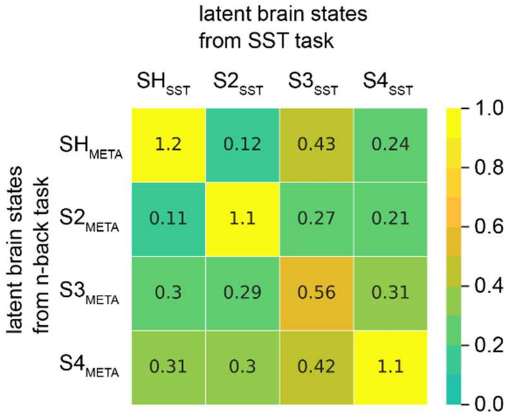

b. State temporal closeness

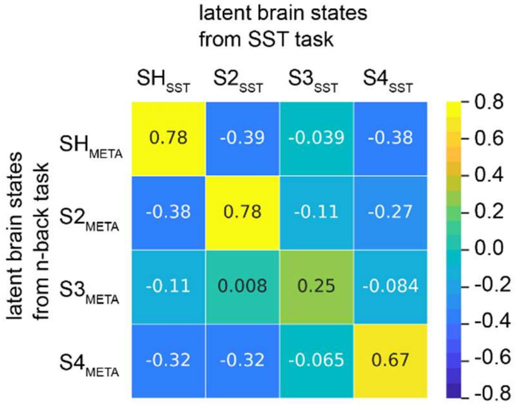

Supplementary Figure S22. **Latent brain states are associated with task performance in the SST with ROIs derived from an independent meta-analysis.** (**a**) Multivariate CCA revealed significant canonical correlations between occupancy rates of latent brain states and behavioral variables in SST (N=45). (**b**) Univariate *Pearson's* correlation analysis revealed that OR of SH_{SST} is significantly correlated with cognitive control index in the SST. SH_{SST} refers to the dynamic brain state that matches to SH_{META} in the SST. ACC = Accuracy; RT = Reaction Time; US = Unsuccessful Stopping; SSD = Stop Signal Delay; SSRT = Stop Signal Reaction Time; OR = Occupancy rate. The regression estimate is presented with 95% confidence interval (shaded area). Source data are provided as a Source data file.

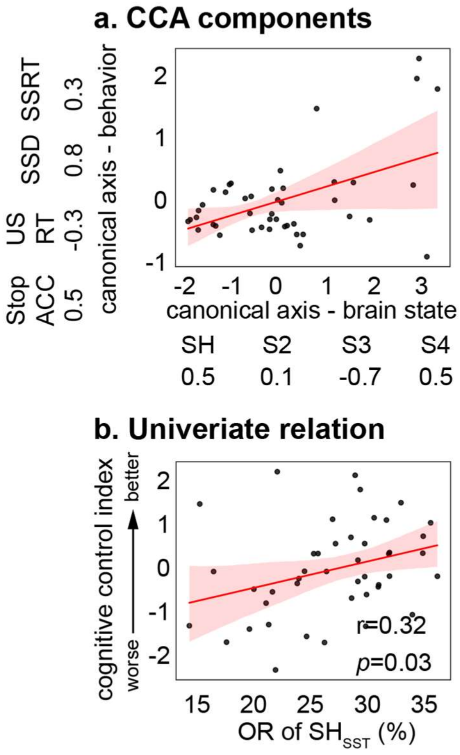

Supplementary Figure S23. **Shared latent brain state across two sessions of the RP task (reference is SH_{META}) with ROIs derived from an independent meta-analysis. (a, b)** BSDS uncovered 4 dynamic brain states in both Sessions 1 and 2 (N=90). SH_{RP1} has the highest state space closeness (c=16) and high state temporal closeness (r=0.97) with SH_{META} in Session 1. SH_{RP2} has the highest state space closeness (c=7.2) and high state temporal closeness (r=0.9) with SH_{META} in Session 2. SH_{META} refers to the high-load dynamic brain state in the n-back working memory task with ROIs derived from an independent meta-analysis. SH_{RP1} and SH_{RP2} refers to the dynamic brain states that matches to SH_{META} in the Relational task session 1 and 2, respectively. Color bars are the scales for state space closeness and state temporal closeness.

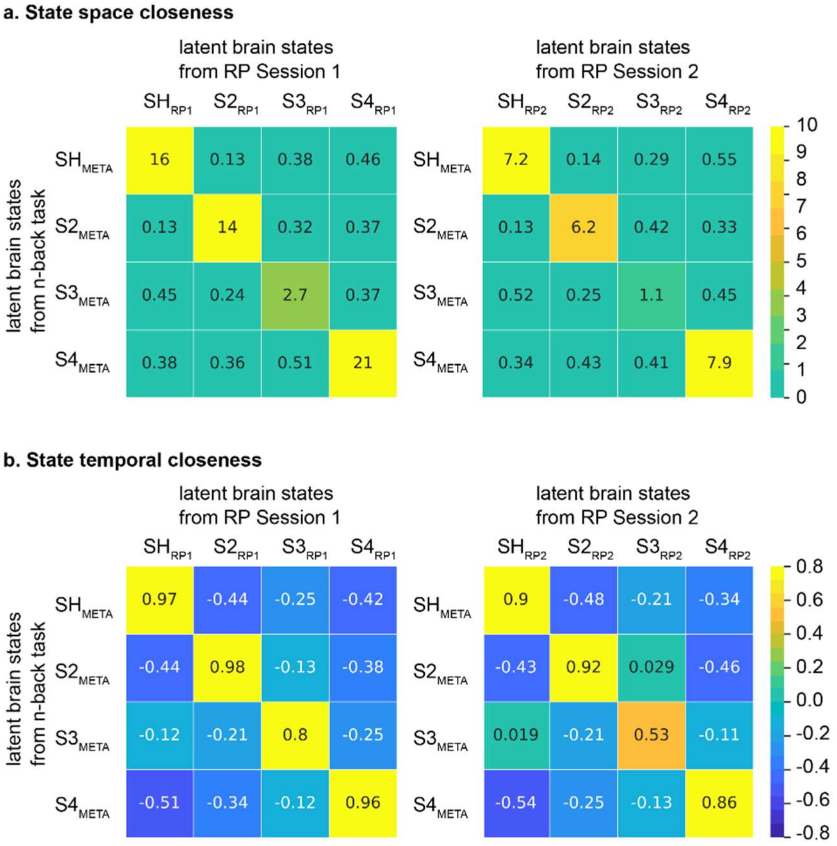

Supplementary Figure S24. **Latent brain states are associated with task performance in each session of the RP task with ROIs derived from an independent meta-analysis.** Multivariate CCA revealed significant correlations between occupancy rates of latent brain states and behavioral variables in the HCP RP task session 1 (**a**) and 2 (**b**) (N=90). Univariate *Pearson's* correlation analysis revealed that OR of SH_{SST} is significantly correlated with cognitive control index in the HCP RP session 1 (**c**) and 2 (**d**). SH_{RP1} and SH_{RP2} refers to the dynamic brain states that matches to SH_{WM} in the Relational task session 1 and 2, respectively. HCP = Human Connectome Project; RP = Relational Processing; MC = Matching Control; ACC = Accuracy; RT = Reaction Time; OR = Occupancy rate. The regression estimate is presented with 95% confidence interval (shaded area). Color bars are the scales for state space closeness and state temporal closeness. P values were not adjusted for multiple comparisons. Source data are provided as a Source data file.

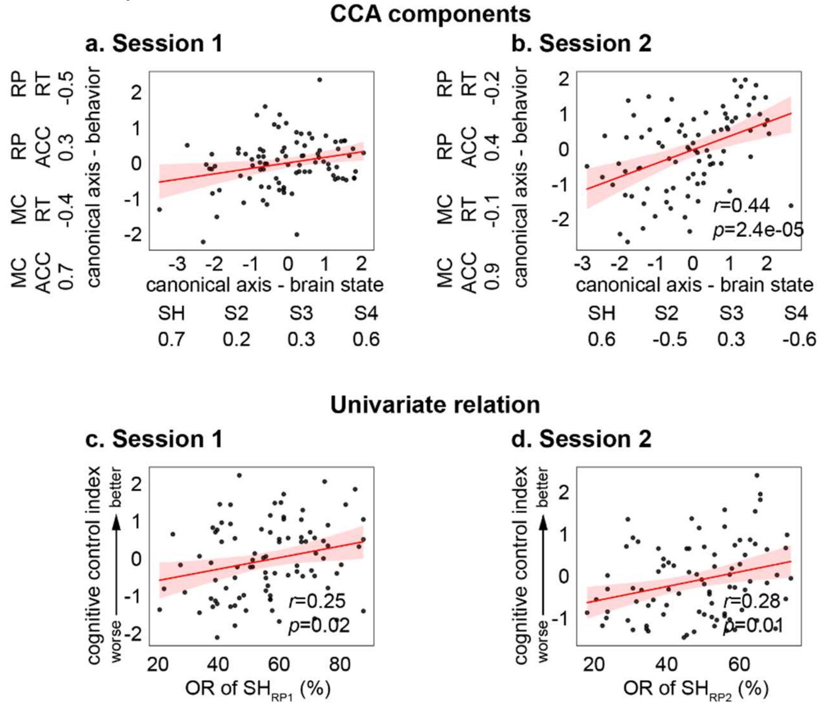

IV. Supplementary Tables

Table S1. **Seven cognitive tasks across four datasets used in the present study.** Tasks are listed in the order analyzed. HCP = Human Connectome Project; DMCC = Dual Mechanisms of Cognitive Control; N-back = n-back working memory task; AxCPT = Ax continuous performance task; CuedTS = Cued task switching task; Sternberg = Sternberg working memory task; Stroop = Stroop interference task; SST = Stop signal task; RP = Relational processing; TR = Time Repetition.

Dataset	Cognitive Tasks	Sample Size	Age	Gender (F/M)	TR (ms)
**HCP**	N-back	90	22-36	53/37	720
**DMCC**	AxCPT<br>CuedTS<br>Sternberg<br>Stroop	50	19-42	31/19	1200
**Stanford**	SST	45	9-12	22/23	490
**HCP**	RP	90	22-36	53/37	720

Supplementary Table S2. Behavioral results for AxCPT, CuedTS, Sternberg and Stroop tasks in the DMCC study. Con: congruent; Incon = Incongruent. P values were not adjusted for multiple comparisons. Source data are provided as a Source data file.

Task/Condition	*Accuracy (%)*	*RT (ms)*	*Task Comparisons*	*t-value*	*Cohen's d*	*p-value*
**AxCPT**						
AX	95±7	437±100	AY - AX Acc	0.04	0.011	0.097
AY	95±8	583±93	AY - AX RT	17.44	4.933	<0.001
BX	86±13	614±170	BX - BY Acc	4.61	1.303	<0.001
BY	85±8	480±106	BX - BY RT	8.02	2.268	<0.001
**CuedTS**						
Con	96±6	1113±224	Incon - Con Acc	4.7	1.517	<0.001
Incon	93±7	1150±216	Incon - Con RT	3.2	0.533	0.002
**Stern**						
LL	88±10	989±153	HL -LL Acc	4.02	1.138	0.0002
HL	84±10	1014±169	HL - LL RT	2.69	0.761	0.01
**Stroop**						
Con	99±1	862±121	Incon - Con Acc	4.45	1.259	<0.001
Incon	97±5	987±123	Incon - Con RT	13.76	3.892	<0.001

Supplementary Table S3. **Canonical correlation analysis of brain-behavior relations in the four dual model of cognitive control (DMCC) tasks.** Canonical correlation weights for brain state and behavioral performance measures are shown. OR = Occupancy Rate; Con: congruent; Incon = Incongruent.

**AxCPT**		**CuedTS**		**Stern**		**Stroop**	
Brain State OR		*Brain State OR*		*Brain State OR*		*Brain State OR*	
SH_{AxCPT}	0.52	SH_{CuedTS}	0.78	SH_{Stern}	0.5	SH_{Stroop}	0.13
$S2_{AxCPT}$	0.48	$S2_{CuedTS}$	-0.13	$S2_{Stern}$	0.19	$S2_{Stroop}$	-0.37
$S3_{AxCPT}$	0.3	$S3_{CuedTS}$	0.43	$S3_{Stern}$	-0.77	$S3_{Stroop}$	-0.84
$S4_{AxCPT}$	-0.63	$S4_{CuedTS}$	0.43	$S4_{Stern}$	-0.33	$S4_{Stroop}$	-0.37
Behavior		*Behavior*		*Behavior*		*Behavior*	
AX RT	0.21	Con ACC	-0.44	LL ACC	0.65	Con ACC	-0.63
AY RT	0.59	Con RT	0.85	LL RT	-0.36	Con RT	0.63
BX RT	-0.35	Incon ACC	0.31	HL ACC	0.24	Incon ACC	-0.33
BY RT	-0.7	Incon RT	-0.03	HL RT	0.62	Incon RT	-0.3

Supplementary Table S4. **Behavioral results for the SST task.** ACC = Accuracy; RT = Reaction Time; US = Unsuccessful Stopping. Source data are provided as a Source data file.

	Mean ± Std
Go ACC (%)	94±5
Go RT (ms)	521±86
Stop ACC (%)	51±6
US RT (ms)	464±72
SSRT (ms)	292±59

Supplementary Table S5. **Canonical correlation analysis of brain-behavior relations in the SST.** Canonical correlation weights for brain states and behavioral performance measures are shown. OR = Occupancy Rate.

**SST**	
Brain State OR	
SH_{SST}	0.62
$S2_{SST}$	0.34
$S3_{SST}$	-0.62
$S4_{SST}$	-0.33
Behavior	
Stop ACC	0.45
US RT	-0.51
SSD	0.71
SSRT	0.18

Supplementary Table S6. Behavioral results for the RP task. MC = Matching Control; ACC = Accuracy; RT = Reaction Time. P values were not adjusted for multiple comparisons. Source data are provided as a Source data file.

	ACC (%)	*RT (ms)*	*Contrast*	*t-value*	*Cohen's d*	*p-value*
Session 1						
RP	73±20	1938±344	RP – MC ACC	9.19	1.937	<0.001
MC	93±10	1450±209	RP – MC RT	16.71	3.523	<0.001
Session 2						
RP	64±19	2001±368	RP – MC ACC	11.7	2.467	<0.001
MC	88±10	1473±212	RP – MC RT	14.8	3.121	<0.001

Supplementary Table S7. **Canonical correlation analysis of brain-behavior relations in the RP task.** Canonical correlation weights for latent brain state and task performance measures are shown. MC = Matching Control; ACC = Accuracy; RT = Reaction Time.

**RP session 1**		**RP session 2**	
Brain State OR		*Brain State OR*	
SH_{RP1}	0.2	SH_{RP2}	0.61
$S2_{RP1}$	0.16	$S2_{RP2}$	-0.66
$S3_{RP1}$	-0.9	$S3_{RP2}$	0.3
$S4_{RP1}$	-0.35	$S4_{RP2}$	-0.31
Behavior		*Behavior*	
MC ACC	-0.16	MC ACC	0.85
MC RT	-0.95	MC RT	0.03
RP ACC	-0.09	RP ACC	0.44
RP RT	0.23	RP RT	-0.29

Supplementary Table S8. **Summary of brain-behavior relations across tasks.** Multivariate relation analysis showed significant canonical correlation between occupancy rates of latent brain states and behavioral performance in each of the seven cognitive control tasks. Univariate relation analysis showed significant *Pearson's* correlation between occupancy rate of the multi-demand latent brain state (e.g. SH_{AxCPT}) and cognitive control index in each cognitive control task. AxCPT: A-x continued performance task; SST: Stop-signal task; RP1/2: Relational Processing session 1/2. P values were not adjusted for multiple comparisons.

**Dataset/Task**	**Canonical Correlation Analysis**		**Univariate Analysis**	
DMCC				
	r	*p*	*r*	*p*
AxCPT	0.51	0.0001	0.34	0.02
CuedTS	0.59	8.3E-06	0.28	0.05
Sternberg	0.65	3.0E-07	0.37	0.01
Stroop	0.59	6.9E-06	0.32	0.02
Stanford				
SST	0.61	1.2E-05	0.33	0.03
HCP				
RP1	0.35	0.0007	0.32	0.002
RP2	0.44	1.3E-05	0.28	0.01

Supplementary Table S9. **Cognitive control measures used to assess univariate brain-behavior relations in each cognitive task.** AxCPT: A-x continued performance task; SST: Stop-signal task; RP1 and RP2: Relational Processing tasks in sessions 1 and 2; SSRT: Stop-signal reaction time; RT = Reaction Time.

**Task**	**Cognitive Control Measure**
AxCPT	(AY RT -BX RT)/(AY RT+BX RT)
CuedTS	Congruent RT - Incongruent RT
Sternberg	High-load Accuracy/High-load RT
Stroop	Congruent RT - Incongruent RT
SST	1/SSRT
RP1	Relational Accuracy/Relational RT
RP2	Relational Accuracy/Relational RT

Supplementary Table S10. **OR of latent brain states in relation to indices of cognitive control in each task.** In each task, the state that matched SH_{WM} was the only state with significant and positive contribution to the cognitive control index. P values were not adjusted for multiple comparisons.

	r	p
AxCPT		
SH_{AxCPT}	0.34	0.02
$S2_{AxCPT}$	-0.01	0.9
$S3_{AxCPT}$	0.1	0.5
$S4_{AxCPT}$	-0.3	0.03
CuedTS		
SH_{CuedTS}	0.28	0.05
$S2_{CuedTS}$	-0.1	0.5
$S3_{CuedTS}$	-0.03	0.8
$S4_{CuedTS}$	0.1	0.5
Stern		
SH_{Stern}	0.36	0.009
$S2_{Stern}$	0.07	0.6
$S3_{Stern}$	-0.06	0.7
$S4_{Stern}$	-0.26	0.06
Stroop		
SH_{Stroop}	0.33	0.02
$S2_{Stroop}$	0.09	0.5
$S3_{Stroop}$	0.08	0.6
$S4_{Stroop}$	0.07	0.6
SST		
SH_{SST}	0.33	0.03
$S2_{SST}$	-0.21	0.16
$S3_{SST}$	-0.4	0.006
$S4_{SST}$	0.13	0.4
RP1		
SH_{RP1}	0.28	0.009
$S2_{RP1}$	-0.2	0.06
$S3_{RP1}$	-0.04	0.7
$S4_{RP1}$	-0.33	0.002
RP2		
SH_{RP2}	0.32	0.002
$S2_{RP2}$	-0.22	0.04
$S3_{RP2}$	-0.05	0.6
$S4_{RP2}$	-0.21	0.05

Supplementary Table S11. OR of latent brain states in SST in relation to the clinical measures of inattention. The SH_{SST} state that matched SH_{WM} was the only state with significant and positive contribution to clinical attention scores. P values were not adjusted for multiple comparisons.

	r	***p***
SH_{SST}	0.39	0.008
$S2_{SST}$	-0.09	0.6
$S3_{SST}$	-0.31	0.04
$S4_{SST}$	0.14	0.4

Supplementary Table S12. **MNI coordinates of the brain network nodes.** Nodes were determined based on activation and deactivation peaks in the contrast of 2-back versus 0-back in the HCP n-back working memory task.

**Node**	***x***	***y***	***z***
lAI	-32	24	2
rAI	36	22	0
lMFG	-42	24	30
rMFG	40	36	34
lFEF	-26	2	58
rFEF	30	10	56
lIPL	-46	-44	44
rIPL	52	-40	50
PCC	-12	-56	16
VMPFC	-2	48	-8
DMPFC	4	16	50

Supplementary Table S13. **State matching to SH_{WM} in each cognitive task.** Space and temporal closeness of states that matched SH_{WM} were statistically significant. P values were not adjusted for multiple comparisons.

Task	space closeness		temporal closeness	
	d	*p*	*r*	*p*
AxCPT				
SH_{AxCPT}	1.2	0.01	0.83	0.01
$S2_{AxCPT}$	0.27	0.38	-0.3	0.81
$S3_{AxCPT}$	0.19	0.6	-0.3	0.81
$S4_{AxCPT}$	0.12	0.7	-0.37	0.96
CuedTS				
SH_{CuedTS}	0.88	0.01	0.72	0.01
$S2_{CuedTS}$	0.51	0.05	0.22	0.26
$S3_{CuedTS}$	0.14	0.91	-0.29	0.8
$S4_{CuedTS}$	0.18	0.71	-0.37	0.9
Stern				
SH_{Stern}	0.86	0.05	0.7	0.01
$S2_{Stern}$	0.34	0.59	-0.16	0.38
$S3_{Stern}$	0.19	0.84	-0.2	0.44
$S4_{Stern}$	0.14	0.93	-0.46	0.91
Stroop				
SH_{Stroop}	1	0.01	0.82	0.01
$S2_{Stroop}$	0.39	0.15	-0.047	0.34
$S3_{Stroop}$	0.17	0.78	-0.31	0.73
$S4_{Stroop}$	0.16	0.87	-0.43	0.92
SST				
SH_{SST}	1.4	0.01	0.77	0.05
$S2_{SST}$	0.27	0.56	-0.3	0.61
$S3_{SST}$	0.26	0.61	-0.12	0.34
$S4_{SST}$	0.17	0.83	-0.38	0.79
RP1				
SH_{RP1}	13	0.1	0.97	0.01
$S2_{RP1}$	0.4	0.33	-0.46	0.87
$S3_{RP1}$	0.26	0.81	-0.29	0.51
$S4_{RP1}$	0.12	0.96	-0.4	0.75
RP2				
SH_{RP2}	7.7	0.04	0.95	0.01
$S2_{RP2}$	0.42	0.2	-0.47	0.96
$S3_{RP2}$	0.27	0.75	-0.19	0.43
$S4_{RP2}$	0.13	0.98	-0.44	0.77

V. Supplementary references

1. Van Essen DC, *et al.* The Human Connectome Project: a data acquisition perspective. *NeuroImage* **62**, 2222-2231 (2012).

2. Braver TS, Kizhner A, Tang R, Freund MC, Etzel JA. The dual mechanisms of cognitive control (DMCC) project. *Journal of Cognitive Neuroscience* **33**, 1990-2015 (2021).

3. Taghia J, *et al.* Uncovering hidden brain state dynamics that regulate performance and decision-making during cognition. *Nat Commun* **9**, (2018).

4. Bishop CM. *Pattern Recognition and Machine Learning*. Springer (2006).

5. Everitt BS. *An introduction to latent variable models*. Chapman & Hall (1984).

6. Ghahramani Z, Beal MJ. Variational inference for Bayesian mixtures of factor analysers. *Adv Neur In* **12**, 449-455 (2000).

7. Fox E, Sudderth E, Jordan M, Willsky A. Nonparametric Bayesian learning of switching dynamical systems. *Advances in Neural Information Processing Systems* **21**, 457-464 (2009).

8. Hutchison RM, *et al.* Dynamic functional connectivity: promise, issues, and interpretations. *NeuroImage* **80**, 360-378 (2013).

9. Friston KJ, Holmes AP, Worseley KJ, Poline CD, Frith CD, Frackowiak RS. Statistical parametric maps in functional imaging: A general linear approach. *Human brain mapping* **2**, 189-210 (1995).

10. McLaren DG, Ries ML, Xu G, Johnson SC. A generalized form of context-dependent psychophysiological interactions (gPPI): a comparison to standard approaches. *NeuroImage* **61**, 1277-1286 (2012).

11. Hotelling H. *Relations between two sets of variables*. Biometrika (1936).

12. Smith SM, *et al.* A positive-negative mode of population covariation links brain connectivity, demographics and behavior. *Nature neuroscience* **18**, 1565-1567 (2015).
