# Supplementary material for: A multi-demand operating system underlying diverse cognitive tasks
Source: Nat Commun. 2024 Mar 11;15:2185. doi: 10.1038/s41467-024-46511-5 (PMC10928152; doi:10.1038/s41467-024-46511-5)
Supplement: Supplementary file 2 — Reporting Summary [file 41467_2024_46511_MOESM2_ESM.pdf]

nature portfolio

Corresponding author(s): Cai

Last updated by author(s): Feb 15, 2024

Reporting Summary

Nature Portfolio wishes to improve the reproducibility of the work that we publish. This form provides structure for consistency and transparency in reporting. For further information on Nature Portfolio policies, see our Editorial Policies and the Editorial Policy Checklist.

Statistics

For all statistical analyses, confirm that the following items are present in the figure legend, table legend, main text, or Methods section.

n/a	Confirmed	
☐	☒	The exact sample size (n) for each experimental group/condition, given as a discrete number and unit of measurement
☐	☒	A statement on whether measurements were taken from distinct samples or whether the same sample was measured repeatedly
☐	☒	The statistical test(s) used AND whether they are one- or two-sided<br>*Only common tests should be described solely by name; describe more complex techniques in the Methods section.*
☐	☒	A description of all covariates tested
☐	☒	A description of any assumptions or corrections, such as tests of normality and adjustment for multiple comparisons
☐	☒	A full description of the statistical parameters including central tendency (e.g. means) or other basic estimates (e.g. regression coefficient) AND variation (e.g. standard deviation) or associated estimates of uncertainty (e.g. confidence intervals)
☐	☒	For null hypothesis testing, the test statistic (e.g. F, t, r) with confidence intervals, effect sizes, degrees of freedom and P value noted<br>*Give P values as exact values whenever suitable.*
☒	☐	For Bayesian analysis, information on the choice of priors and Markov chain Monte Carlo settings
☒	☐	For hierarchical and complex designs, identification of the appropriate level for tests and full reporting of outcomes
☐	☒	Estimates of effect sizes (e.g. Cohen's d, Pearson's r), indicating how they were calculated

Our web collection on statistics for biologists contains articles on many of the points above.

Software and code

Policy information about availability of computer code

Data collection	Eprime was used to collect behavioral data in the Stanford dataset.
Data analysis	Functional MRI data preprocessing and statistical analyses were performed on the SPM12 and FSL 6, and Matlab 2020. BSDS and State Matching Code can be accessed at Github (https://github.com/scsnl/Cai_Multiple_Demand_System_2023)

For manuscripts utilizing custom algorithms or software that are central to the research but not yet described in published literature, software must be made available to editors and reviewers. We strongly encourage code deposition in a community repository (e.g. GitHub). See the Nature Portfolio guidelines for submitting code & software for further information.

Data

Policy information about availability of data

All manuscripts must include a data availability statement. This statement should provide the following information, where applicable:

- Accession codes, unique identifiers, or web links for publicly available datasets
- A description of any restrictions on data availability
- For clinical datasets or third party data, please ensure that the statement adheres to our policy

All original data reported in this study are publicly available on Zenodo: https://zenodo.org/records/10702914. Source data are provided in this paper.

Research involving human participants, their data, or biological material

Policy information about studies with human participants or human data. See also policy information about sex, gender (identity/presentation), and sexual orientation and race, ethnicity and racism.

Reporting on sex and gender	Gender differences were not analyzed in the study due to the absence of any prior hypotheses regarding gender-specific effects. The findings are applicable to individuals of all genders. Gender information was collected on self-report.
Reporting on race, ethnicity, or other socially relevant groupings	We do not have access to race, ethnicity or other socially relevant groupings information in the data.
Population characteristics	We do not use these information in our study.
Recruitment	We used the public Human Connectome Project and Dual Mechanism of Cognitive Control datasets. For Stanford dataset, participants were recruited in a broad geographic region around the San Francisco Bay Area through flyers.
Ethics oversight	Human Connectome Project and Dual Mechanism of Cognitive Control studies were approved by the Institutional Review Board of the Washington University in St. Louis. The Stanford study was approved by the Institutional Review Board of Stanford University.

Note that full information on the approval of the study protocol must also be provided in the manuscript.

Field-specific reporting

Please select the one below that is the best fit for your research. If you are not sure, read the appropriate sections before making your selection.

☒ Life sciences ☐ Behavioural & social sciences ☐ Ecological, evolutionary & environmental sciences

For a reference copy of the document with all sections, see nature.com/documents/nr-reporting-summary-flat.pdf

Life sciences study design

All studies must disclose on these points even when the disclosure is negative.

Sample size	We performed a power analysis based on the state-behavior relationship identified from our previous study. If we set alpha at p=0.05, a sample size of 39 will provide power of 0.8 to detect the effect of interest.
Data exclusions	For the HCP dataset, the following criteria were used: (1) complete behavioral and brain imaging data in two different acquisition sessions; (2) range of head motion in any translational and rotational direction less than 1 voxel; (3) average scan-to-scan head motion less than 0.25 mm. For the DMCC dataset: the following criteria were used: (1) complete behavioral and brain imaging data; (2) range of head motion in any translational and rotational direction was less than 1 voxel in all the tasks; (3) average scan-to-scan head motion was less than 0.25 mm in all the tasks.
Replication	State-behavior relation was replicated across 7 different cognitive tasks tested in the study.
Randomization	no group allocation in this study
Blinding	no group allocation in this study

Reporting for specific materials, systems and methods

We require information from authors about some types of materials, experimental systems and methods used in many studies. Here, indicate whether each material, system or method listed is relevant to your study. If you are not sure if a list item applies to your research, read the appropriate section before selecting a response.

Materials & experimental systems

n/a	Involved in the study	
☒	☐	Antibodies
☒	☐	Eukaryotic cell lines
☒	☐	Palaeontology and archaeology
☒	☐	Animals and other organisms
☒	☐	Clinical data
☒	☐	Dual use research of concern
☒	☐	Plants

Methods

n/a	Involved in the study	
☒	☐	ChIP-seq
☒	☐	Flow cytometry
☐	☒	MRI-based neuroimaging

Magnetic resonance imaging

Experimental design

Design type: task state, block and event-related design

Design specifications: For Dual control tasks, each participant completes two runs of each task. In the AxCPT, the full condition includes 216 trials (72 AX, 18 AY, 18 BX, 72 BY, 18 A-No-Go, 18 B-No-Go). In the CuedTS, 3 task blocks alternated with 4 resting fixation blocks (30 sec duration) within each run. The Sternberg working memory task included 4 different memory set list-lengths (5, 6, 7 and 8 items). The full condition included 90 trials. In the Stroop task, the full condition includes 72 congruent and 144 incongruent trials.
For stop-signal task, each participant completes two runs and each run includes 96 trials.
For relational processing task, each participants completes two runs. Each condition had 3 blocks. There were 4 trials in each relational processing block and 5 trials in each control matching block.

Behavioral performance measures: Reaction time and accuracy

Acquisition

Imaging type(s): functional MRI

Field strength: 3T

Sequence & imaging parameters: Multi-band sequence was used for fMRI data acquisition in the Dual Mechanism of Cognitive Control study TR=1200ms, TE=33ms, flip angle=45°, in-plan resolution=2.4mm and multiband factor=4
Multi-band sequence was used for fMRI data acquisition in the Human Connectome Project study, TR=720 ms, TE=33.1 ms, flip angle=52°; in-plane resolution=2 mm and multiband factor=8.

Area of acquisition: Whole-brain acquisition

Diffusion MRI: ☐ Used ☒ Not used

Preprocessing

Preprocessing software: Minimal processed fMRI data were obtained from the Human Connectome Project and Dual Mechanism of Cognitive Control and underwent additional spatial smoothing with a Gaussian kernel of 6mm FWHM using SPM12.

Normalization: fMRI data was normalized using standard nonlinear transformation steps implemented in SPM12.

Normalization template: MNI152 2mm template was used.

Noise and artifact removal: 6 head motion regression is regressed out

Volume censoring: No volume censoring was done because a stringent head motion criterion was used for data inclusion

Statistical modeling & inference

Model type and settings: We used Bayesian Switching Dynamic Systems algorithm to model latent brain state during task performance. Canonical correlation and pearson's correlation were used to test state-behavior association

Effect(s) tested: We tested whether latent brain state is correlated with cognitive control functions across tasks.

Specify type of analysis: ☐ Whole brain ☒ ROI-based ☐ Both

Anatomical location(s) | ROIs are determined in a previous study.

Statistic type for inference (See Eklund et al. 2016) | N/A

Correction | FDR corrected.

Models & analysis

n/a	Involved in the study
☐	☒ Functional and/or effective connectivity
☒	☐ Graph analysis
☒	☐ Multivariate modeling or predictive analysis

Functional and/or effective connectivity | Functional connectivity was modeled as latent features in brain state dynamic modeling
